# Update of the European Society of Anaesthesiology and Intensive Care Medicine evidence-based and consensus-based guideline on postoperative delirium in adult patients

**DOI:** 10.1097/EJA.0000000000001876

**Published:** 2023-08-30

**Authors:** César Aldecoa, Gabriella Bettelli, Federico Bilotta, Robert D. Sanders, Paola Aceto, Riccardo Audisio, Antonio Cherubini, Colm Cunningham, Wojciech Dabrowski, Ali Forookhi, Nicola Gitti, Kaisa Immonen, Henrik Kehlet, Susanne Koch, Katarzyna Kotfis, Nicola Latronico, Alasdair M.J. MacLullich, Lior Mevorach, Anika Mueller, Bruno Neuner, Simone Piva, Finn Radtke, Annika Reintam Blaser, Stefania Renzi, Stefano Romagnoli, Maria Schubert, Arjen J.C. Slooter, Concezione Tommasino, Lisa Vasiljewa, Bjoern Weiss, Fatima Yuerek, Claudia D. Spies

**Affiliations:** From the Department of Anaesthesia and Postoperative Critical Care, Hospital Universitario Rio Hortega, Valladolid, Spain (CA), Department of Biomedical Studies, University of the Republic of San Marino, San Marino (GB), Department of Anesthesiology, Critical Care and Pain Medicine, 'Sapienza’ University of Rome, Rome, Italy (FB, AF, LM), Specialty of Anaesthetics & NHMRC Clinical Trials Centre, University of Sydney & Department of Anaesthetics and Institute of Academic Surgery, Royal Prince Alfred Hospital (RDS), Department of Anesthesiology and Intensive Care Medicine, Charité-Universitätsmedizin Berlin, Corporate Member of Freie Universität Berlin, and Humboldt Universität zu Berlin, Campus Charité Mitte, and Campus Virchow Klinikum (CDS, SK, AM, BN, LV, BW, FY), Dipartimento di Scienze dell’Emergenza, Anestesiologiche e della Rianimazione, Fondazione Policlinico Universitario A. Gemelli IRCCS, Rome, Italy (PA), Dipartimento di Scienze Biotecnologiche di Base, Cliniche Intensivologiche e Perioperatorie, Università Cattolica del Sacro Cuore, Rome, Italy (PA), Department of Surgery, Institute of Clinical Sciences, Sahlgrenska University Hospital, Göteborg, Sweden (RA), Geriatria, Accettazione Geriatrica e Centro di ricerca per l’invecchiamento, IRCCS INRCA, Ancona, Italy (AC), School of Biochemistry and Immunology and Trinity College Institute of Neuroscience, Trinity College, Dublin, Ireland (CC), First Department of Anaesthesiology and Intensive Care Medical University of Lublin, Poland (WD), Research Unit of Nursing Science and Health Management, University of Oulu, Oulu, Finland (KI), Section of Surgical Pathophysiology, Copenhagen University Hospital, Rigshospitalet, Copenhagen, Denmark (HK), Department of Anesthesiology, Intensive Therapy and Acute Intoxications, Pomeranian Medical University in Szczecin, Poland (KK), Department of Medical and Surgical Specialties, Radiological Sciences and Public Health, University of Brescia (NG, NL, SP, SR), Department of Anesthesia, Critical Care and Emergency, Spedali Civili University Hospital, Brescia, Italy (NL, SP), Edinburgh Delirium Research Group, Ageing and Health, Usher Institute, University of Edinburgh, Edinburgh, United Kingdom (AMJM), Department of Anaesthesia and Intensive Care, Nykoebing Hospital; University of Southern Denmark, SDU (SK, FR), Department of Anaesthesiology and Intensive Care, University of Tartu, Tartu, Estonia (ARB), Center for Intensive Care Medicine, Luzerner Kantonsspital, Lucerne, Switzerland (ARB), Department of Health Science, Section of Anesthesiology, University of Florence (SR), Department of Anaesthesia and Critical Care, Azienda Ospedaliero-Universitaria Careggi, Florence, Italy (SR), School of Health Sciences, Institute of Nursing, ZHAW Zurich University of Applied Science, Winterthur, Switzerland (MS), Departments of Psychiatry and Intensive Care Medicine, UMC Utrecht Brain Center, University Medical Center Utrecht, Utrecht University, Utrecht, the Netherlands (AJCS), Department of Neurology, UZ Brussel and Vrije Universiteit Brussel, Brussels, Belgium (AJCS) and Dental Anesthesia and Intensive Care Unit, Polo Universitario Ospedale San Paolo, Department of Biomedical, Surgical and Odontoiatric Sciences, University of Milano, Milan, Italy (CT)

## Abstract

Postoperative delirium (POD) remains a common, dangerous and resource-consuming adverse event but is often preventable. The whole peri-operative team can play a key role in its management. This update to the 2017 ESAIC Guideline on the prevention of POD is evidence-based and consensus-based and considers the literature between 01 April 2015, and 28 February 2022. The search terms of the broad literature search were identical to those used in the first version of the guideline published in 2017. POD was defined in accordance with the DSM-5 criteria. POD had to be measured with a validated POD screening tool, at least once per day for at least 3 days starting in the recovery room or postanaesthesia care unit on the day of surgery or, at latest, on postoperative day 1. Recent literature confirmed the pathogenic role of surgery-induced inflammation, and this concept reinforces the positive role of multicomponent strategies aimed to reduce the surgical stress response. Although some putative precipitating risk factors are not modifiable (length of surgery, surgical site), others (such as depth of anaesthesia, appropriate analgesia and haemodynamic stability) are under the control of the anaesthesiologists. Multicomponent preoperative, intra-operative and postoperative preventive measures showed potential to reduce the incidence and duration of POD, confirming the pivotal role of a comprehensive and team-based approach to improve patients’ clinical and functional status.

## Introduction

Postoperative delirium (POD) is widely accepted as a topic of important medical and public health relevance. It not only impacts on the health, the well being and the life perspective of those who experience this adverse postoperative complication, but it often has severe consequences for the families, the healthcare system and the society as a whole. In recent years, awareness of its pathophysiological pathways, its clinical manifestations and its prevention has increased. POD develops when anaesthesia-related and surgery-related precipitating factors interact with a patient's predisposing vulnerability to delirium. Because of this, assessing the preoperative physical, cognitive, mental and social status of a patient scheduled for surgery is essential to quantify a patient's overall risk for POD and to tailor the optimal preoperative, intra-operative and postoperative treatment.

The aim of this updated guideline on the prevention and management of POD was to summarise the evidence in adults published since the end of the last literature search in March 2015.^1^ The suggestions and recommendations on the prevention and treatment and – if required – aftercare of POD are based on those of the first version of the guideline and on the new evidence.

Because of the large amount of new literature, the working group split up into six subgroups.

## General methods

### General approach for all working groups

The current broad literature search followed the same search strategy as outlined in the previous version of the guideline.^1^ The new literature search encompassed the periods from 01 April 2015, until 30 November 2020 (search 1) and from 01 December 2020 until 28 February 2022 (search 2).

Identical search terms as for the previous version of this guideline were used^1^:

(delirium OR confusion OR confusion∗ OR disorientation OR bewilderment) AND (postoperative OR postoperative period OR postoperative period∗ OR postsurgical OR postsurgical OR anesthesia recovery period OR anesthesia recovery period∗ OR post anesthesia).

The 2291 (search 1) and 979 (search 2) references retrieved were screened using the following exclusion criteria (‘screening step 1’):

case reports, case series reports, comments, letters to the editor, editorials, errata, replies, study protocols, non-English publications, studies in paediatric patients or in patients less than 18 years, POD outcome not clearly defined, nonsurgical patients, or mixed surgical and nonsurgical patients with no separate presentation of surgical patients results, POD summarised among other postoperative complications such as ‘neurological complications’ or combinations of POD with postoperative cognitive dysfunction (POCD).

**Fig. 1:**
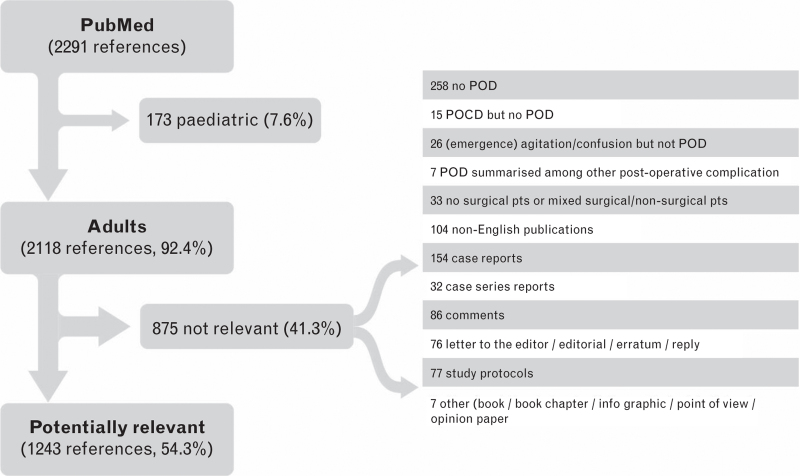
STEP 1: flow chart of the study selection process from April 2015 until November 2020 (search 1).

**Fig. 2:**
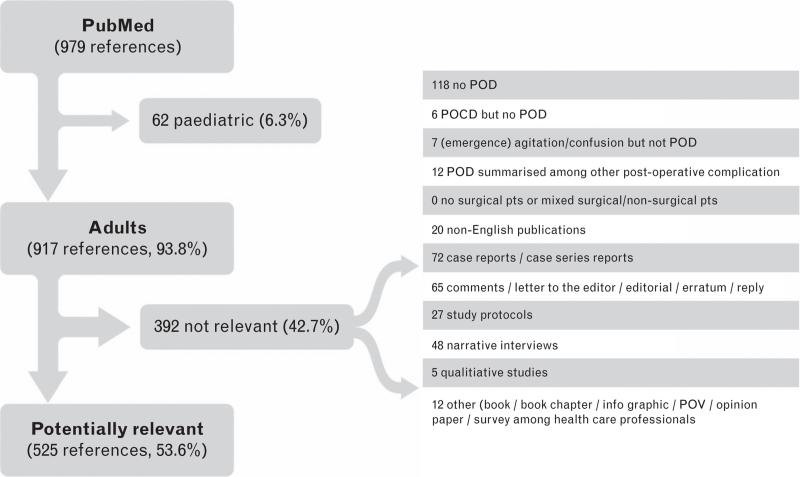
STEP 2: flow chart of the study selection process December 2020 until February 2022 (search 2).

Altogether 1243 + 525 references were assigned to the six working groups (see Figs. 1 and 2 and the algorithm for the assignment in Supplement Table S1 and Supplement Figure S1).

*Definition of POD*: For recommendations and suggestions, the Task Force and the Advisory Board agreed to include as underlying evidence, solely studies which used a validated POD screening tool (see Supplement Table S2), at least once per day (preferably two or three times per day) for at least 3 days, starting in the recovery room or in the PACU on the day of surgery or latest on postoperative day 1. It was further agreed that even when systematic reviews and meta-analyses existed, single studies in these already published systematic reviews and meta-analyses had to be screened again for the above-mentioned POD inclusion criteria. Single studies fulfilling the above-mentioned POD definition were used for the update of this guideline. This criterion was required for all kinds of studies including observational studies.

The detailed literature reviews and eventual specific literature searches of the six working groups are listed in the Supplement.

The presentation of recommendation follows GRADE (Grading of Recommendations, Assessment, Development and Evaluations) methodology.^2^ The GRADE approach often involves numbers and letters being used to express the quality of evidence and strength of a recommendation. These approaches may lead to semantic confusion.^3^ Therefore, we decided to use the full-text description of quality of evidence and strength of recommendation according to the GRADE handbook.^4^

## Results and recommendations

### Chapter 1: Basic Science

#### Authors: Colm Cunningham, Robert D. Sanders, Bjoern Weiss

The Basic Science working group provided – based on the results of the above-mentioned broad literature search – a narrative review of the research carried out in this field since 2015. After extensive discussion, the task force unanimously agreed that no explicit recommendations should be made in this section of the updated guideline. Rather, the areas that need further investigation and the weaknesses of the existing evidence should be highlighted to encourage more thorough research into the mechanisms underlying the emergence, existing risk factors, development and treatment of POD. Research on the pathophysiology of POD using animal models has been dominated by a dual focus on the deleterious effects of anaesthesia and those of inflammation. Although there are studies suggesting deleterious impacts of anaesthesia alone on hyperphosphorylation of Tau and other pathological features in animals, it is also the case that normal working memory and attention return within 1 to 2 h after general anaesthesia in humans.^5^ Although some complex interactions between anaesthetics and inflammation may occur, research published in the last 7 years (i.e. this review period) has shown an increasing focus on inflammation. The main animal models employed by researchers in this research period are laparotomy and tibial fracture (with occasional use of hepatolobectomy and cardiopulmonary bypass). These surgical interventions produce acute tissue injury and, therefore, cause the production/release of damage-associated molecular patterns (DAMPs) such as S100 proteins, nucleic acids and High Mobility Group Box-1 (HMGB1), which are analogous to, and activate the same receptors as, pathogen-associated molecular pattern (PAMPs), such as bacterial lipopolysaccharide (LPS) – also known as ‘endotoxin’ – and pathogen nucleic acids that arise during infection. These DAMPs activate various inflammatory receptors to induce acute inflammatory responses in the periphery and also trigger some neuroinflammatory changes. The changes most commonly described in models of POD include circulating cytokines, induction of inflammatory transcripts in the brain, blood brain barrier impairments and changes in the morphology or number of microglial cells (brain resident macrophage population) and astrocytes. These inflammatory changes are now quite well established in the POD field, as they have been in the infection-induced delirium field.^6,7^ There have now been studies blocking individual cytokines,^8,9^ depleting microglia^10^ or inhibiting microglia/macrophages/monocytes^11^ or blocking inflammatory pathways by exploiting natural anti-inflammatory pathways, such as restoring levels of acetylcholine^12^ and increasing resolvins,^13^ which are endogenous lipid mediators of inflammatory resolution. Other lipid-derived mediators, prostaglandins, blocked by cyclooxygenase inhibitors, have also been shown to contribute to neurophysiological and behavioural features of delirium, albeit in LPS models.^14,15^ Most of these anti-inflammatory treatments reduce the intensity or duration of inflammation not only in the brain but also in the periphery, although increased blood–CSF barrier permeability may facilitate closer interactions between peripheral and central processes.^16^ Most of the studies above report positive impacts on ‘POD-like’ changes in these animals.

However, these findings come with significant caveats. The nature of cognitive characterisation of animals in the postsurgical phase remains a weakness in this literature. It is essential to establish cognitive and behavioural tasks that inform on dysfunction in cognitive domains relevant to clinical delirium, but many studies rely on measures for which the level of disruption may be disproportionately weighted by typical responses to surgical trauma or illness, like anxiety, inactivity and suppression of appetite, which are significant confounders of many of the cognitive tasks used in this field. Inattention is a core feature of delirium and several studies, using tasks for memory consolidation, locomotor activity/exploration, food retrieval and novel object recognition, have inappropriately used the terms attention/inattention to describe tasks despite not measuring attention. There has been a tendency to re-label the behavioural measure actually recorded using terms that allow it to conform with criteria for delirium. Thus, there is a strong imperative to measure specific cognitive features, using tasks specifically designed for that feature and to report data using precise terminology appropriate to the task actually undertaken.

Independent of behavioural assessment, measures of cellular and molecular changes that occur in the postsurgical period provide relevant information, although it remains important to distinguish between studies that demonstrate causality with respect to POD measures and those that merely show associations. Dexmedetomidine has shown some pro-autophagic and anti-inflammatory effects, which may be relevant to protection^17,18^ whereas sevoflurane has shown both pro-inflammatory and anti-inflammatory effects in different studies.^19–21^ Growth factors including brain-derived neurotrophic factor (BDNF), netrin1 and mesencephalic astrocyte-derived neurotrophic factor (MANF),^22–25^ as well as disrupted energy metabolism/regulation,^19,26,27^ mitochondrial dysfunction and oxidative stress^23,28^ have all been shown to occur with surgery. Many of these are associations only but interventions targeting some of these dysregulated pathways have been reported to alter behavioural outcomes.

To summarise, the field is still in its infancy and further research is urgently required, but the best current information would support the idea that acute inflammation is a better predictor of postoperative cognitive changes than is anaesthesia. More precise cognitive testing will be required to assess whether the observed cognitive changes represent a delirium-like syndrome. As most studies have used young healthy animals, it is not intuitive that the changes observed in those studies with young healthy animals would reach the severity of delirium, which is more often associated with patients who are older, frailer or suffer from underlying dementia. Studies using older animals or with models of underlying degenerative disease have more often been performed in animals receiving bacterial LPS rather than surgery,^29,30^ but similar studies are beginning to emerge in post-operative studies: with larger effects of tibial fracture described in animals with prior amyloid pathology^31^ and significant effects of laparotomy in older animals.^32^ It will remain important to employ behavioural tasks that interrogate specific cognitive domains and to control for injury-associated or illness-associated confounds. Finally, molecular findings from animal model studies will need to be validated in the relevant patient cohorts to examine the extent to which inflammatory^33^ and brain injury markers^34^ are associated with delirium and long-term outcomes.

#### Outlook

There is now reasonable evidence that peripheral inflammation and, in turn, neuroinflammation contribute to acute deficits resembling delirium. The data that anaesthetics do likewise are significantly weaker. The alpha-2 agonist sedative drug dexmedetomidine has shown promise, and basic evidence is emerging that it also acts in an anti-inflammatory manner. This requires confirmation in human studies. There remains relatively little exploration of the idea that anti-inflammatory approaches more broadly might be helpful in patients. Given the caution around use of NSAIDs in older patients, evidence for beneficial effects will have to be pursued in experimental medicine or clinical trials. One recent randomised controlled trial demonstrated that postoperative intravenous acetaminophen was effective in the prevention of delirium^35^; however, it is already typically prescribed for its analgesic effects. Detailed collection of data informing on inflammatory, metabolic and hypoxic changes is required in the peri-operative setting to support adoption of new clinical strategies to mitigate POD.

### Chapter 2: Risk Factors

#### Authors: Federico Bilotta, Ali Forookhi, Henrik Kehlet, Lior Mevorach, Stefano Romagnoli

From the 1243 articles of the initial broad literature search (see Supplement Figure S2 for details), 484 articles identified POD risk factors.^36^ The Risk Factor working group adopted a broader POD definition than the above-mentioned POD definition (see Definition of POD in the general methods section). The minimum required screening duration was 24 h (and not 72 h) and all studies were included as long as they screened for POD, using a validated screening tool at least once during the 24 h following surgery. When applying these criteria, 196 articles remained. Sixty-eight out of the 196 articles were included in a quantitative synthesis (meta-analysis) based on the following criteria: standardised methodology for measurement; more than five studies conducted on the variables (Fig. S2).

#### Meta-analyses

Based on a recent publication,^36^ the following recommendations can be given:

**Table:** 

Recommendation 2.1	Quality of the evidence	Strength of recommendation
We recommend evaluating the following preoperative risk factors for POD: (1) older age, (2) American Society of Anesthesiology Physical status score > 2, (3) Charlson Comorbidity Index >2 and (4) Mini Mental State Examination score lower than 25 points	*Moderate*	**Strong**

Of note, for individual POD prevention planning and prehabilitation, a geriatric assessment evaluating frailty, sensory impairment, malnutrition, polypharmacy, anaemia and other risk factors, including social risk factors is warranted (see the current evidence on geriatric assessment and multicomponent interventions in Chapter 4). Although the Mini-mental state Examination (MMSE) is often used in clinical research, its use in clinical practice is limited by copyright restrictions. Freely available alternatives are the Montreal Cognitive Assessment (MOCA),^37^ the Mini-Cog test^38^ and the Addenbrooke's Cognitive Examination – Revised (ACE-R)^39^ or the Addenbrooke's Cognitive Examination III (ACE-III).^40^

Although the cut-offs presented in Recommendation 2.1 imply risk escalates significantly at certain thresholds, it is important to recognise that risk entails a continuum of disease and that the risk factors above similarly represent a continuous risk factor. This means that as the risk factors scale (e.g. age increases), it is reasonable to estimate that, on average, risk of delirium also increases. Furthermore, risk factors may interact and even synergise. For example, the available evidence suggests increasing delirium risk after the age of 60 years old, however impaired cognitive function likely interacts, increasing delirium risk for a given age.

### Chapter 3: Preventive Measures I: Effects of Drugs on POD prevention

#### Authors: Katarzyna Kotfis, Annika Reintam Blaser, Antonio Cherubini, Wojciech Dabrowski, Nicola Gitti, Nicola Latronico, Bruno Neuner, Simone Piva, Stefania Renzi

The Preventive Measures-1 working group performed a systematic review assessing the effect of dexmedetomidine on POD, with an a priori-defined aim to include only randomised controlled trials (RCTs). In addition to the original search (described before), we assessed existing systematic reviews (SR) to identify additional studies that were not retrieved by our search (see Supplement Figure S3). None of the existing systematic reviews used specific criteria regarding validated screening tools and repeated assessment for POD as defined for this guideline update. For further details, see the Supplementary Material.

### PICO 1 (preventive use of dexmedetomidine preoperatively, intra-operatively or postoperatively vs. non-dexmedetomidine/placebo)

#### PICO 1A: Dexmedetomidine vs. placebo

**Fig. 3:**
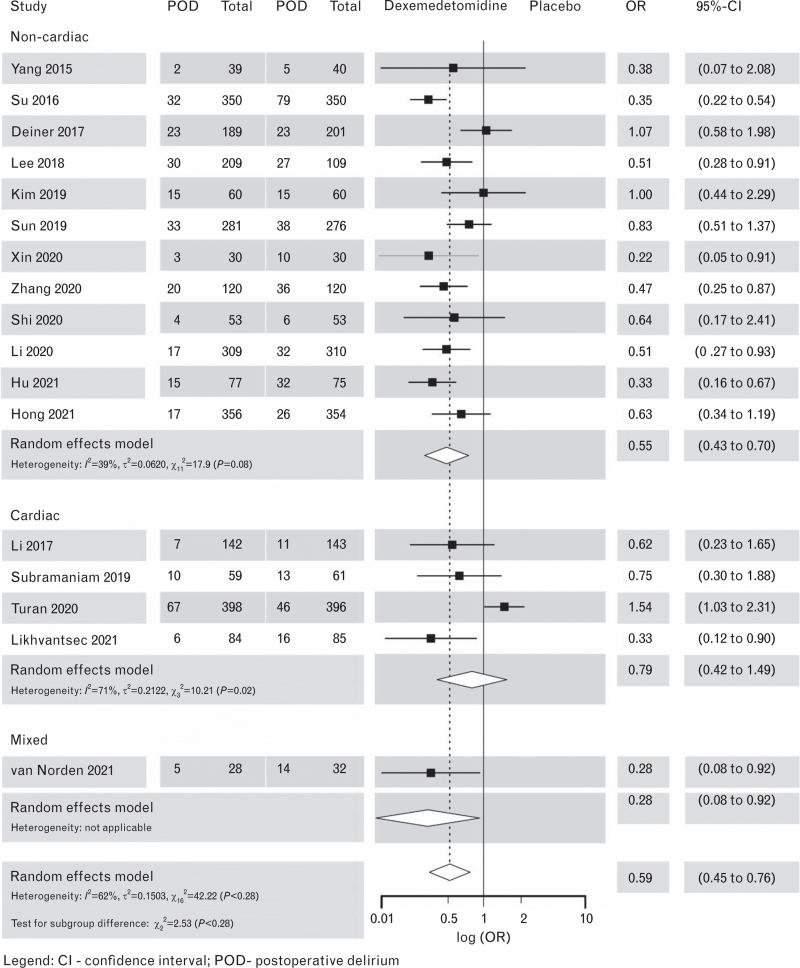
Forest plot for postoperative delirium outcomes in dexmedetomidine vs. placebo.

CI, confidence interval; POD, postoperative delirium.

When compared with placebo, dexmedetomidine was associated with a lower incidence of POD in noncardiac surgery patients, but not in cardiac surgery patients, while pooling the two subgroups resulted in a significant effect on reduction of POD but with high heterogeneity (Fig. 3).^35,41–56^

The level of certainty of the evidence was initially high, originating exclusively from RCTs. However, the evidence was downgraded by two levels due to high heterogeneity and indirectness (Supplement Table S3).

#### PICO 1B: dexmedetomidine vs. other drugs

**Fig. 4:**
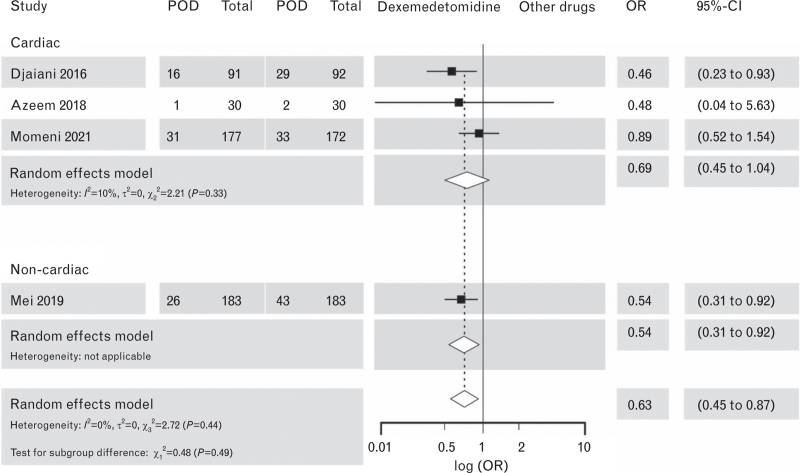
Forest plot for postoperative delirium outcomes in dexmedetomidine vs. other drugs.

CI, confidence interval; POD, postoperative delirium. The other drugs were propofol in all the studies except Azeem^58^ (2018) using midazolam + morphine in the control group.

Dexmedetomidine, when compared with other drugs, was associated with a reduction of POD in patients both after noncardiac and cardiac surgery (Fig. 4).^57–60^ After excluding the study using clonidine as the comparator,^61^ the effect in the cardiac surgery subgroup was no longer significant (Fig. 4, upper part).

The level of certainty of the evidence was initially high originating exclusively from RCTs. However, the evidence was downgraded by two levels due to inconsistency and indirectness (Supplement Table S4).

#### Adverse effects

We analysed bradycardia and hypotension as side effects of dexmedetomidine with all the studies pooled because of an insufficient number of studies for the analysis in subgroups. Dexmedetomidine was associated with bradycardia [odds ratio (OR) 1.60; 95% confidence interval (CI), 1.30 to 1.96], see Supplement Figure S4) and hypotension (OR 1.23, 95% CI, 1.04 to 1.45), see Supplement Figure S5).

**Table:** 

Recommendation 3.1	Quality of the evidence	Strength of recommendation
In patients undergoing surgery, we do **not** suggest the use of any drug as a prophylactic measure to reduce the incidence of POD.	*Low* Mei *et al.*, 2018^60^ Yang *et al.*, 2015^41^ Su *et al.*, 2016^42^ Deiner *et al.*, 2017^43^ Lee *et al.*, 2018^44^ Kim *et al.,* 2019^45^ Sun *et al.*, 2019^46^ Xin *et al.*, 2021^55^ Zhang *et al.*, 2020^55^ Shi *et al.*, 2020^47^ Li *et al.*, 2020^48^ Hu *et al.,* 2021^49^ Hong *et al.*, 2021^50^ Li *et al.*, 2017^51^ Subramaniam *et al.*, 2019^35^ Turan *et al.*, 2020^52^ Likhvantsev *et al.*, 2021^53^ Van Norden *et al.*, 2021^54^ Djaiani *et al.*, 2016^57^ Azeem *et al.*, 2018^58^ Momeni *et al.*, 2021^59^	**Weak**

Recommendation 3.2	Quality of the evidence	**Strength of recommendation**
When dexmedetomidine is used intra-operatively or postoperatively with the aim to prevent POD, we recommend balancing the expected benefits against the most important side effects (bradycardia and hypotension).	*Moderate* Djaiani *et al.*, 2016^57^ Su *et al.*, 2016^42^ Li *et al.*, 2017^51^ Deiner *et al.*, 2017^43^ Subramaniam *et al.* 2019^35^ Sun *et al.*, 2019^46^ Xin *et al.*, 2021^55^ Zhang *et al.*, 2020^56^ Turan *et al.*, 2020^52^ Shi *et al.*, 2020^47^ Li *et al.*, 2020^48^ Hu *et al.*, *2021*^49^ Hong *et al.*, 2021^50^ Van Norden *et al.*, 2021^52^	**Strong**

Our rationale not to suggest dexmedetomidine for the prevention of POD in general, despite its apparent positive effects in some of the studies, is based on these main reasons: the concern about cardiovascular side effects, the selectiveness of the study populations and the heterogeneity of treatment effect in available studies. Additionally, the aspect of a prophylactic use was considered important and the principle of ‘first do no harm’ followed. Our Evidence-to-Decision process is presented in Supplement Tables S5 to S8.

In summary, there is a possibility that there is a patient group that may benefit from intra-operative and/or postoperative dexmedetomidine, but this specific group and details of intervention (timing and dosage) remain to be defined. As age is a risk factor for delirium, the desired effect is probably more likely to occur in older patients; however, the results of recent systematic reviews^62,63^ assessing subgroups based on age are conflicting.

### PICO 2 (preventive use of neuroleptics preoperatively, intra-operatively, or postoperatively vs. nonneuroleptics/placebo)

There was one RCT that met the inclusion criteria. Khan *et al.*^64^ included 135 patients undergoing thoracic surgery and infused a low dose of haloperidol (0.5 mg three times daily for a total of 11 doses) postoperatively. They found that low-dose haloperidol given postoperatively did not reduce the incidence of POD.

### PICO 3 (preventive use of sleep medications preoperatively or postoperatively vs. no sleep medications/placebo)

Three studies (two RCTs and one observational study) evaluated the effects of sleep medications, such as melatonin or ramelteon (a strong agonist of melatonin receptors) on POD prevention. Shi^65^ administered melatonin (3 mg for 7 days, starting on the day of surgery) and compared it with a placebo, in a good-quality pilot RCT, in 288 patients who underwent percutaneous transluminal coronary intervention (PCI). The incidence of POD was significantly lower in the melatonin group than in the placebo group (27.0 vs. 39.6%, respectively, *P* = 0.02). In a second RCT (also of good quality),^66^ ramelteon (8 mg) or placebo was administered starting from the night prior to the surgery up to 8 days, in 120 patients who underwent elective pulmonary thromboendarterectomy, with no statistically significant differences in the two study arms (36% placebo vs. 32.2% ramelteon; relative risk (RR) 0.9, 95% CI, 0.5 to 1.4, *P* = 0.656). Finally, Artemiou *et al.*^67^ carried out an observational study in a group of 250 patients (good quality), administering 5 mg of melatonin from the day before surgery to postoperative day 3. The incidence of delirium was 8.4% in the melatonin group vs. 20.8% in the control group (*P* = 0.001).

### PICO 4 (preventive use of cholinesterase inhibitors preoperatively or postoperatively vs. no use of cholinesterase inhibitors)

One good-quality RCT^68^ evaluated the effects of physostigmine (a bolus of 0.02 mg kg^−1^ body weight followed by 0.01 mg kg^−1^ body weight h^−1^ vs. placebo) for the prevention of POD in 261 patients who underwent elective liver surgery. The incidence of POD did not differ significantly between the physostigmine and placebo groups (20 vs. 15%; *P* = 0.334).

### PICO 5 (other drugs: application of a drug to reduce POD vs. no application of any specific drug to reduce POD)

Eighteen RCTs^35,69–85^ evaluated the effects of different drugs on the prevention of POD, but no conclusive effects could be drawn because of the high heterogeneity of the intervention and the variable quality of studies.

### PICO 6 (anaesthetic drugs: intravenous anaesthetics vs. inhalation anaesthetics)

Only one study met the inclusion criteria. Mei *et al.*^86^carried out a single-centre pilot RCT, including 209 patients aged at least 60 years old undergoing total hip/knee replacement, who were randomised to either a propofol or sevoflurane group. Days of POD per person were higher in the propofol (0.5 ± 0.8) anaesthesia group compared with the sevoflurane anaesthesia group (0.3 ± 0.5, *P* = 0.049).

### PICO 7 (anaesthetic drugs: ketamine intra-operatively or postoperatively vs. no ketamine)

One study met the inclusion criteria. Avidan *et al.*^87^ carried out a good-quality RCT that enrolled 672 patients older than 60 years undergoing major cardiac or noncardiac surgery under general anaesthesia. Patients were randomised to one of the three groups: placebo (0.9% saline), low-dose ketamine (0.5 mg kg^−1^) or high-dose ketamine (1.0 mg kg^−1^) after induction of anaesthesia. There was no difference in delirium incidence between patients in the combined ketamine groups and the placebo group (19.45 vs. 19.82%, respectively; absolute difference of 0.36%, 95% CI, −6.07 to 7.38, *P* = 0.92), but there were more postoperative hallucinations (*P* = 0.01) and nightmares (*P* = 0.03) with increasing ketamine doses compared with placebo.

### PICO 8 (type of anaesthesia: regional anaesthesia vs. general anaesthesia)

Seven studies met the inclusion criteria (six RCTs and one observational) with six out of seven studies showing no difference in the incidence of POD between regional anaesthesia and general anaesthesia. A RCT by Tang *et al.*^88^ that aimed to compare the combined lumbar-sacral plexus block (CLSB) plus general anaesthesia with the unilateral spinal anaesthesia in 124 elderly patients undergoing hip fracture surgery, showed no significant differences in the incidence of POD (5.5 vs. 7.3%, *P* = 0.57). The RCT by Brown *et al.*^89^ (217 patients aged ≥65 years) found that spinal anaesthesia with targeted sedation based on BIS values compared with general anaesthesia with masked BIS values did not reduce the incidence of delirium after lumbar fusion (25.2 vs. 18.9%; *P* = 0.26). In a large RCT by Li *et al.*^90^ (950 patients aged ≥65 years undergoing hip fracture surgery), regional anaesthesia without sedation did not significantly reduce the incidence of POD as compared with general anaesthesia (unadjusted risk difference, 1.1%; 95% CI, −1.7 to 3.8%; *P* = 0.48). In a large RCT by Neuman *et al.*^91^ (1600 patients aged ≥50 years) to evaluate spinal anaesthesia compared with general anaesthesia for hip fracture, the incidence of POD was similar with both types of anaesthesia (20.5 vs. 19.7%, RR, 1.04; 95% CI, 0.84 to 1.30). In a RCT by Strike *et al.*^92^ (*n* = 44) regarding a transapical transcatheter aortic valve replacement (TAVR) procedure, the patients were assigned to either the paravertebral group (perioperative continuous thoracic paravertebral block with a local anaesthetic) or the patient-controlled analgesia group (systemically administered opioids), with no difference in the rate of POD (23 vs. 27%, *P* = 0.73). In an observational study by Vlisides *et al.*^93^ in a group of 263 surgical patients, postoperative epidural use was not associated with a reduced overall incidence of delirium (adjusted OR, 0.65; 95% CI, 0.32 to 1.35; *P* = 0.25). In a large RCT by Li *et al.*,^94^ 1720 patients aged 60 to 90 years were scheduled for major noncardiac thoracic or abdominal surgery and POD was less common in the combined epidural-general anaesthesia group (1.8%) than in the general anaesthesia group (5.0%); with RR, 0.351; 95% CI [0.197 to 0.627]; *P* < 0.001; number needed to treat (NNT) = 31.

### PICO 9 (surgery: minimally invasive surgery [except laparoscopy] vs. more invasive surgery [except laparotomy])

There were no studies on the effects of minimally invasive surgery that met the inclusion criteria for POD assessment.

### PICO 10 (surgery: laparoscopy vs. laparotomy)

One study met the inclusion criteria. In an observational study, Shin *et al.*^95^ compared POD in elderly patients following laparoscopic gastrectomy vs. open gastrectomy in 130 patients aged at least 65 years with gastric cancer. In both groups, the overall incidence of POD was not significantly different: 31.6% (19/60) in the laparoscopic gastrectomy group and 41.2% (26/63) in the open gastrectomy group (*P* = 0.359).

### PICO 11 (cardiac surgery: off-pump in cardiac surgery vs. on-pump in cardiac surgery)

Only one study met the inclusion criteria. Szwed *et al.*^96^ carried out a good-quality RCT on 192 patients scheduled for elective isolated off-pump coronary bypass (OPCAB) and randomised patients to three parallel arms: 1. The first study arm underwent anaortic OPCAB (ANA) with total arterial revascularisation. 2. The second study arm underwent OPCAB with vein grafts using carbon dioxide surgical field flooding (CO_2_FF). 3. The control arm underwent ‘conventional’ OPCAB with vein grafts. The incidence of POD was the lowest in the ANA group [12.5% in the ANA group vs. 32.8% in the CO_2_FF arm, and 35.9% in the control (OPCAB) arm, *P* = 0.006].

**Table:** 

Recommendation 3.3	Quality of the evidence	Strength of recommendation
In patients undergoing surgery, we do **not** suggest any specific type of surgery or type of anaesthesia to reduce the incidence of POD.	*Low* Mei *et al.*, 2018^60^ Avidan *et al.*, 2017^87^ Brown *et al.*, 2021^89^ Li *et al.*, 2022^90^ Neuman *et al.*, 2021^91^ Vlisides *et al.*, 2019^93^ Li *et al.*, 2021^94^ Shin *et al.*, 2015^95^ Szwed *et al.*, 2021^96^	**Weak**

### PICO 12 (biomarkers: abnormal value of a biomarker preoperatively, intra-operatively, or postoperatively vs. normal level of a biomarker)

Overall 39 single studies were identified evaluating different biomarkers for POD (see Supplement Table S9). Studied biomarkers can be categorised as follows: oxidative stress markers, markers of nerve cell alteration, markers of neurogenesis and synaptic plasticity, markers of axonal damage, markers of neuroglia injury (blood-brain barrier disruption), inflammation markers, systemic noninflammation markers and genetic markers. None of the studies showed specific biomarkers with sufficiently high sensitivity and specificity in predicting and/or confirming POD. Hence, no recommendation can be made regarding the practical use of any biomarker in preventing POD or in the early identification of patients at risk of POD.

**Table:** 

Recommendation 3.4	Quality of the evidence	Strength of recommendation
We do **not** suggest using biomarkers to identify patients at risk of POD.	*Low* (References in the Supplement Table S9)	**Weak**

### Chapter 4: Preventive Measures II: Nonpharmacological Interventions

#### Authors: Gabriella Bettelli, Paola Aceto, Riccardo Audisio, Antonio Cherubini, Bruno Neuner, Maria Schubert, Fatima Yuerek

The Preventive Measures II working group aimed to answer the following PICO question.

### PICO 13: Which nonpharmacological multicomponent or single interventions can be recommended to prevent POD?

P patients undergoing surgery

I multicomponent or single nonpharmacological intervention/s

C usual care

O POD (according to the definition outlined in the ‘General methods’ section).

To answer the question on single component or multicomponent nonpharmacological interventions to prevent POD, PM-2 used the results of the broad literature search described in the general approach for all working groups. Out of 1243 + 525 potentially relevant studies (see Figs. 1 and 2 in the main text), 250 studies were potentially relevant as they were on single component or multicomponent nonpharmacological interventions. We further intended to address issues of team management during the implementation process. The detailed flow chart (Supplement Figure S6) and the screening process are shown in the supplement. Of the 19 studies selected, 8 RCTs were multicomponent interventions,^97–102^ and 11 RCTs were single interventions.^105–115^

As stated in the chapter on risk factors, the risk model for POD is composed of patients’ underlying clinical and functional vulnerabilities (the so-called patient-related ‘predisposing’ risk factors) and the surgery-related and anaesthesia-related (‘precipitating’) factors, such as induced inflammatory reaction, anatomical site, length and invasiveness of the procedure. Depending on the local organisation, the preoperative risk assessment is done by anaesthesiologists – independently if they have the legal responsibility, or other specialists might be involved, such as geriatricians or neurologists.

**Table:** 

Recommendation 4.1	Quality of the evidence	Strength of recommendation
We recommend that preoperative anaesthesia consultation in older adults includes the screening for risk factors for POD and addresses patients’ needs to optimise their preoperative status.	*Low* Dalton and Zafirova, 2018^116^ Lim and Lee, 2020^117^ Carli and Baldini, 2021^118^ Carli *et al.*, 2021^119^	**Strong**

Various predisposing and precipitating factors are nonmodifiable (such as age, ASA physical status and surgical site). Interventions in single modifiable risk factors such as anaemia^120^ or nutritional and hydration deficits^121^ through targeted optimisation strategies showed mixed results in reducing the incidence of POD. Bundles of multicomponent interventions seem more effective in reducing the incidence of POD, both in surgical and nonsurgical hospitalised patients.^122^ This requires a valid picture of a patient's individual risk profile. Consequently, potentially modifiable predisposing vulnerabilities should be identified preoperatively, as a first step toward their evidence-based correction.^116^ The screening for risk factors may integrate routine data from the preoperative anaesthesia consultation (clinical history, medication intake, ASA physical status classification, etc.) and data deriving from more specific screenings such as Comprehensive Geriatric Assessment (CGA) tools (evaluation of cognitive, emotional, sensorial, nutritional and functional deficits, together with the need for family or social support^117^). The effectiveness of this approach depends on the time interval available to implement prehabilitation measures and the urgency of the surgical treatment.^119^ Older adults with frailty and a low functional reserve because of chronic medical conditions particularly benefit from such efforts.^118^

**Table:** 

Recommendation 4.2	Quality of the evidence	Strength of recommendation
We recommend that the results of the screening for POD risk factors are shared among the care team and the preventive strategies discussed and registered in the medical records.	Low Berian *et al.*, 2018^123^ Oh and Park, 2019^124^ Enomoto *et al.*, 2021^125^ Sockalingam *et al*., 2015^126^	**Strong**

The recommendation to share the information inside the team is based on the principle of good clinical practice that there should be a ‘team-based approach’. In fact, many multicomponent interventions require close co-operation among the different team members, which is essentially linked to both a preoperative team-based discussion and agreement among the professionals involved in the realisation of such multicomponent measures.

Both the process of decision-making about surgery and patients’ preoperative optimisation should be planned and managed at a multidisciplinary team level as suggested by the US Coalition for Quality in Geriatric Surgery.^123^ This requires, apart from risk factor screening and preoperative medication management, multidisciplinary conferences for high-risk patients.^123^ Staff training aimed at increasing the understanding of POD, and good communication skills among different medical professions seem prerequisites for the implementation of successful multicomponent interventions to reduce the incidence of POD.^124^ Retrospective data suggest that a combination of staff education, protocols for implementation, preprinted orders and systematic screening for POD reduces its incidence.^125^ Although POD is considered relevant for patients’ outcomes, not all teams communicate delirium care plans during handover and not all professionals are confident about their role in its prevention.^126^

**Table:** 

Recommendation 4.3	Quality of the evidence	Strength of recommendation
We recommend multicomponent nonpharmacological interventions in all patients at risk of POD.	Moderate Olotu *et al*., 2022^101^ Deeken *et al*., 2022^97^ Marcantonio *et al*., 2001^100^ Vidan *et al*., 2005^103^ Guo and Fan, 2016^98^ Partridge *et al*., 2017^102^ Hempenius *et al*., 2013^99^ Wang *et al.*, 2019^79^	**Strong**

The details on the eight RCTs on multicomponent interventions are displayed in the Supplement Tables S10 and S11.

There were two major approaches of multicomponent interventions (see Supplement Table S11): Three-quarters (6/8) of the studies^97,99,100,102–104^ combined a preoperative or early postoperative geriatric assessment with individualised tailored interventions from a set of possible interventions. The dimensions of the geriatric assessments (described in detail only in three studies^97,99,102^) slightly varied between studies. However, all three studies assessed frailty, cognition, comorbidities and functional status/functional impairment. Based on these screenings, a set of ‘best-practice delirium prevention modules’^97^ or a ‘tailored HELP protocol’^102^ was available. Other studies used ‘recommendations based on a structured protocol’.^100^

These elements of geriatric assessment and tailored treatments were brought into the existing teams by an ‘independent delirium study prevention team’,^97^ a ‘geriatric team, which supervised an individual treatment plan with specific attention to patient-related risk factors’,^99^ a ‘proactive geriatrics consultation within 24 h after surgery’^100^ or a ‘geriatric team’ composed of a geriatrician, a rehabilitation specialist and a specific social worker.^101^ This team implemented a ‘complete geriatric evaluation to identify and quantify medical and psychosocial problems and functional capability to elaborate a comprehensive therapeutic plan’.^103^ In another study, the whole procedure took place in an ‘outpatient clinic setting’.^102^

An alternative approach (see again Supplement Table 11) was to train the existing staff and to implement a set of measures for all patients undergoing (elective) surgery.^101^ These ‘delirium prevention bundles’ included reorientation measures, sleeping aids (quiet ward, eye masks/earplugs at night, avoiding caffeinated drinks in the afternoons and evenings), and early mobilisation, early catheter removal and early nutrition commencement.^101^ Another study evaluated preop visits to the ICU, the introduction of the medical equipment used in combination with reorientation strategies, sleep hygiene, catheter removal and early mobilisation and nasal feeding by ‘educated staff’.^98^

When applying the revised risk-of-bias assessment tool (RoB-2),^127^ see Supplement Table S12, four studies were graded with low risk of bias,^97,98,101,102^ and another four studies with a high risk of bias.^99,101,102,128^

**Fig. 5:**
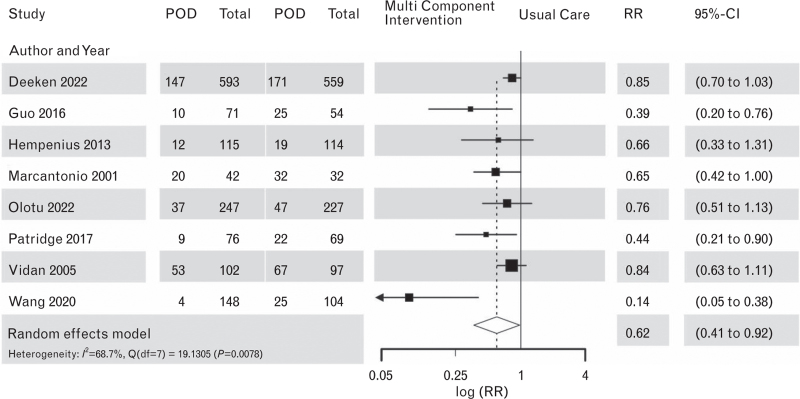
Forest plot for postoperative delirium outcomes in multicomponent interventions vs. usual care, *n* = 8 studies.

Figure 5 displays the results of meta-analyses for all eight RCTs and for the subgroups of the six studies using a combination of geriatric assessment and individualised treatments. The pooled evidence from all eight RCTs indicated a positive effect of multicomponent interventions with a pooled RR = 0.62; 95% CI [0.41 to 0.92]; *P* = 0.0248.

There was clear evidence of publication bias, indicated by an asymmetric funnel plot, see Supplement Figure S7 and a significant regression test for funnel plot symmetry (H_0_ = funnel plot symmetry, i.e., no evidence for publication bias) with *P* = 0.0034.

**Fig. 6:**
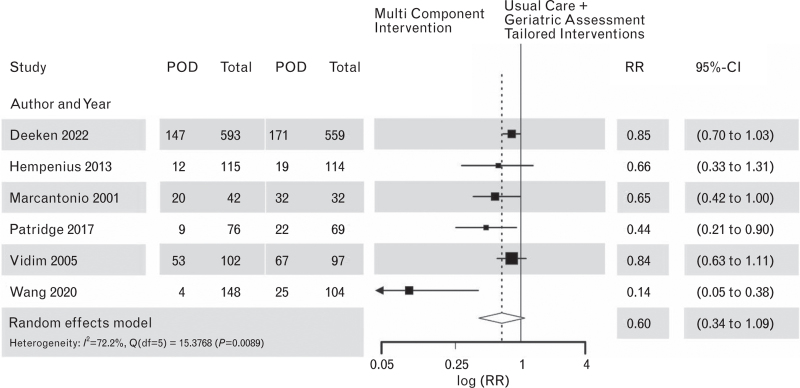
Forest plot for postoperative delirium outcomes in multicomponent interventions vs. usual care after (comprehensive) geriatric assessment plus tailored interventions, *n* = 6 studies.

In the subgroup of the six studies, which evaluated a combination of CGA plus tailored interventions based on the individualised risk profile, there was no overall significant effect of the interventions with RR = 0.60; 95% CI [0.34 to 1.09]; *P* = 0.0797 (Fig. 6).

There was again clear evidence for a publication bias, indicated by an asymmetric funnel plot, see Supplement Figure S8 and a significant regression test for funnel plot symmetry (H_0_ = funnel plot symmetry, i.e. no evidence for publication bias) with *P* = 0.0211.

The level of certainty of the evidence was initially high, only RCTs were pooled. However, the evidence was downgraded due to high heterogeneity (>70%) and indirectness and the results of the risk-of-bias assessment (Supplement Table S12).

#### Rationale

As reported above, POD has a multifactorial genesis, and patients have their own risk profiles for POD. Therefore, it is unlikely that any single intervention is sufficient to reduce POD in all patients (although some will benefit). It is further unlikely that multicomponent interventions (heterogeneously composed because of many factors such as team knowledge and culture, resource availability and internal organisation) will reduce POD in all patients (although again some will benefit). Therefore, interventions should be individualised based on predisposing risk factors and precipitating factors and be preceded by thorough team-based discussion.

Regarding the single interventions, both the study population (see Supplement Table S13) and the interventions (see Supplement Table S14) were so heterogeneous that pooling of the results by meta-analyses was not feasible. Of note, several studies are of major importance for patients’ safety, not only concerning the prevention of POD. This particularly holds for fast-track surgery in hip fracture surgery,^107^ the management of heavy smokers in the peri-operative setting^109^ and the management of patients with severe depression and antidepressant treatment undergoing surgery.^110^ Although guidance in such situations would be desirable, current evidence does not allow confident suggestions or recommendations. Other single interventions such as patient education,^105,113^ music interventions^111^ or cognitive training/enhancement^112,113^ were often integrated in the multicomponent interventions that we summarised in recommendation 3. Of note, fast-track approaches, as in the study by Jia *et al.*,^108^ are usually accompanied by a series of further treatment modifications (see Supplement Table S14). Single studies of rather uncommon interventions for the prevention of POD exist, such as foot reflexology massage^114^ or the use of a structured mirror intervention to ‘positively impact on mental status and attention, thereby enhancing factual encoding’ ‘as opposed to delusion memories after ICU discharge’.^106^ Their usefulness and benefit for the patient have to be proven in further studies.

### Chapter 5: Neuromonitoring

#### Authors: Susanne Koch, Nicola Latronico, Alasdair MacLullich, Simone Piva, Finn Radtke, Robert Sanders, Concezione Tommasino

The detailed flow chart and the screening process are presented in Supplement Figure S9.

Based on a detailed review of the included studies and discussions within our neuromonitoring working group we give the following recommendations:

### PICO 14: Is processed EEG monitoring during anaesthesia able to reduce POD?

**Table:** 

Recommendation 5.1	Quality of the evidence	Strength of recommendation
We suggest Index-based EEG monitoring depth of anaesthesia guidance to decrease the risk of POD.	Low Chan *et al.*, 2013^129^ Radtke *et al.*, 2013^130^ Whitlock *et al*., 2014^131^ Zhou *et al*., 2018^132^ Wildes *et al.*, 2019^133^ Tang *et al.*, 2020^134^ Evered *et al.*, 2021^22^ Wang *et al.*, 2022^135^	**Weak**

**Fig. 7:**
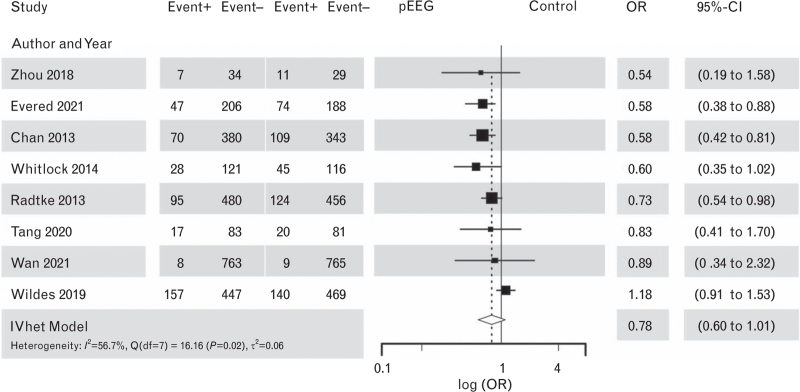
Forest plot using inverse variance heterogeneity model analysis for postoperative delirium outcomes on intraoperative processed EEG Neuromonitoring guidance vs. usual intraoperative care or comparing deep anaesthesia vs. light anaesthesia^22^ in older patients, *n* = 8 studies.

We performed a systematic review and meta-analysis on the effect of processed EEG monitoring on POD, with an a priori-defined aim to include only RCTs. Of the 12 RCTs included, 4 RCTs were not considered in the final systematic review and meta-analysis. Cotae *et al.*^136^ was not explicit in the number of days of POD evaluation, Kunst *et al.*^137^ evaluated POD only on days 3 and 5 and Sponholz *et al.*^138^ did not state how POD was assessed. Finally, the RCT from Xu *et al.*^139^ compared Index-guided anaesthesia vs. multiparameter-guided anaesthesia (Index, burst suppression activity and density spectral array) and was, therefore, excluded from our meta-analysis. Finally, eight studies were included in the meta-analysis.^22,129–135^ The meta-analysis using inverse variance heterogeneity model analysis suggested no significant benefit of processed EEG monitoring in reducing the risk of POD (OR 0.78; 95% CI, 0.60 to 1.01) (Fig. 7).

Although these data may suggest that further trials are needed, a significant limitation is a concentration on index values in the literature. Index values may be less reliable in older people with reduced cortical electrical activity.

Although the borderline significance of the meta-analysis, high heterogeneity and risk of bias (see Supplement Table S15) were all seen, the clinical experts decided to upgrade recommendation 5.1. This is due to balance of benefits and harms, patients’ preferences and feasibility. Given the additional limitations of processed EEG in older persons undergoing anaesthesia – vulnerable patients (with neurodegeneration) are the hardest to monitor (due to low EEG power) – novel approaches such as displaying the density spectral array are required to optimise the care of older patients. Based on previous considerations, we suggest training anaesthesiologists to learn how to interpret raw EEG and density spectral array patterns during intraoperative EEG monitoring to decrease the risk of POD.

### PICO 15: Does multiparameter intraoperative EEG monitoring, focusing on burst suppression activity and including the density spectral array, improve guiding depth of anaesthesia and decrease the risk of POD?

**Table:** 

Recommendation 5.2	Quality of the evidence	Strength of recommendation
We suggest multiparameter, intraoperative EEG monitoring (burst suppression, density spectral array, DSA) during anaesthesia to decrease the risk of POD.	Low Soehle *et al.*, 2015^140^ Fritz *et al.*, 2016^141^ Fritz *et al.*, 2018^142^ Pedemonte *et al.*, 2020^143^ Fritz *et al.*, 2020^144^ Cooter Wright *et al.*, 2022^145^ Acker *et al.*, 2021^146^ Koch *et al.*, 2021^147^ Tanabe *et al.*, 2020^148^ Gutierrez *et al.*, 2019^149^	**Weak**

Ten observational studies were evaluated, five on burst suppression and five on raw EEG (See recommendation 5.2).

#### Burst suppression

Soehle *et al.*^140^ included 81 patients undergoing cardiac surgery and found that delirious patients remained significantly (*P* = 0.018) longer in a burst suppression state intraoperatively (107 min, IQR [47 to 170] vs. 44 min, IQR [11 to 120]) than nondelirious patients. Fritz *et al.*^141^ included 727 adult patients who received general anaesthesia with planned ICU admission and found that patients with prolonged periods of burst suppression were more likely to experience POD (*P* < 0.0001). Another study from the same group included 618 elective surgery patients who underwent anaesthesia with a volatile anaesthetic; patients who experienced electroencephalogram suppression at lower volatile anaesthetic concentrations had a higher incidence of POD (35 vs. 17%) (OR 2.63; 95%CI [1.81 to 3.84]; *P* < 0.001).^142^ Pedemonte *et al.*^143^ carried out a retrospective analysis including 159 patients aged more than 60 years undergoing cardiac surgery and found that burst suppression activity duration was positively related to an increased POD risk (odds ratio, 3.79; 95%CI [1.5 to 9.6]; *P* = 0.005), along with age (OR, 1.09; 95%CI [1.02 to 1.16]; *P* = 0.009), abbreviated Montreal Cognitive Assessment (OR, 0.80; 95%CI [0.66 to 0.97]; *P* = 0.024), and alpha power (OR, 0.75; 95%CI [0.59 to 0.96]; *P* = 0.025). In a secondary analysis from Fritz *et al.*^144^ of the data from the ENGAGES trial, which enrolled 1113 patients aged 60 or older undergoing surgery with general anaesthesia patients were randomised to electroencephalogram-guided anaesthesia or usual care. Four hundred and thirty patients had evidence of preoperative abnormal cognition. Of these 151/430 (35%) patients had POD. Of the total effect size, 2.4%; 95%CI [0.6 to 4.8%] was an indirect effect mediated by electroencephalogram suppression. The author's concluded that a small portion of the total effect of preoperative abnormal cognition on POD was mediated by electroencephalogram suppression.

#### Raw EEG

Cooter Wright *et al.*^145^ included 139 older surgical patients (age ≥65) and analysed the Duke Anesthesia Resistance Scale: the average Bispectral index (BIS) divided by the quantity 2.5 minus the average age-adjusted end-tidal minimum alveolar concentration (aaMAC) inhaled anaesthetic fraction. The relationship between Duke Anesthesia Resistance Scale and delirium risk was nonlinear, with higher delirium risk at lower Duke Anesthesia Resistance Scale scores. Acker *et al.*^146^ applied the multiscale entropy (MSE) in 50 adult patients (≥60 years) before and during surgery and found that MSE was not associated with delirium or attention. Koch *et al.*^147^ included 237 patients aged at least 65 years in an observational study and performed the raw EEG analysis calculating the perioperative spectral edge frequency (SEF). The authors showed that lower preoperative SEF, absence of slowing in EEG while transitioning from preoperative state to unconscious state, and lower EEG power in relevant frequency bands in both these states are related to POD development. Tanabe *et al.*^148^ recruited 70 surgical patients included in an ongoing cohort study (Interventions for POD: Biomarker-3) and performed an EEG slow-wave activity (SWA) analysis on preoperative and postoperative days. They found that changes in occipitoparietal cortical SWA correlated with worsening delirium severity. Gutierrez *et al.*^149^ conducted an exploratory observational study in 30 patients older than 60 years, scheduled for elective major abdominal surgery. The authors found that patients with POD or subsyndromal POD in comparison with the control group had a lower intraoperative absolute alpha-band power during anaesthesia (4.4 ± 3.8 vs. 9.6 ± 3.2 dB, *P* = 0.0004) and a lower relative alpha power (0.09 ± 0.06 vs. 0.21 ± 0.08, *P* < 0.0001), independently of the anaesthetic dose.

#### Discussion

Anaesthesiologists should be trained not only to observe the Index number given by the processed EEG monitors (e.g. PSI or BIS) but also understand and interpret the raw EEG and the density spectral array.^139^

In older patients, deep anaesthesia frequently causes burst suppression activity in the raw EEG,^150^ which has been identified as a risk factor for POD.^129,130,140–143^ This EEG pattern – an isoelectric line with intermittent bursts – can easily be identified in the raw EEG if presented on the monitors. Thus, anaesthesiologists should ensure that the monitors they use provide raw EEG traces.

Additionally, it has been shown that reduced intraoperative alpha-band power is related to an increased risk of POD.^147,149^ Frontal coherent alpha-band and slow-delta-band power physiologically are triggered by a thalamocortical feedback mechanism induced by GABAergic activation.^151^ These frontal coherent alpha and slow-delta bands can be identified in the density spectral array given on the screen of EEG monitors. However, these EEG patterns are not only related to the dose of the anaesthetic agent given but also to age,^150,152^ to the preexisting cognitive function of the patients,^153,154^ cerebral perfusion^155^ and premedication with a benzodiazepine.^156^ Also, it should be noted that this alpha–delta pattern has been associated with connected consciousness intraoperatively^157^ and hence, while it likely represents a state of adequate anaesthesia in the majority, some patients may not be unconscious.

In older patients, the EEG power is generally very low, so the alpha-band power might not be visible on the monitor when using the default power scale, and the operator should modify the sensitivity scale to better see the alpha power in the density spectral array (DSA) screen. This is related to the fact that the power range of fast oscillations such as beta and alpha activity has a lower power than slow oscillations such as theta and delta activity. In general, faster oscillations are associated with concious, cognitive active states and slow oscillations are associated with sleepiness and unconsciousness. Hence, there is a risk that older patients get overdoses of anaesthetic agents, because of false high-level Index parameters. This problem might be overcome if anaesthesiologists can interpret the density spectral array pattern. In older patients, it could be of help to increase the power sensitivity of the monitor, to make alpha-bands more clearly visible, and hence avoid overdosage of anaesthetic agents in these vulnerable patients.

### Chapter 6: Pharmacological Treatment of POD and POD Outcomes

#### Authors: Claudia Spies, Nicola Latronico, Alasdair MacLullich, Anika Müller, Finn Radtke, Lisa Vasiljewa

**Fig. 8:**
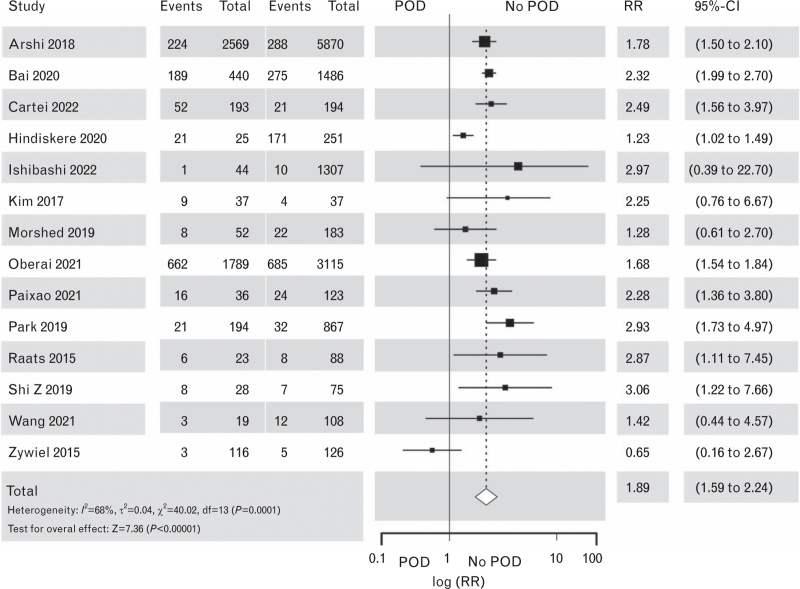
Forest plot for mortality in patients with postoperative delirium after noncardiac surgery vs. patients with no postoperative delirium. The corresponding Funnel plot is shown in Supplement Figure S11.

**Fig. 9:**
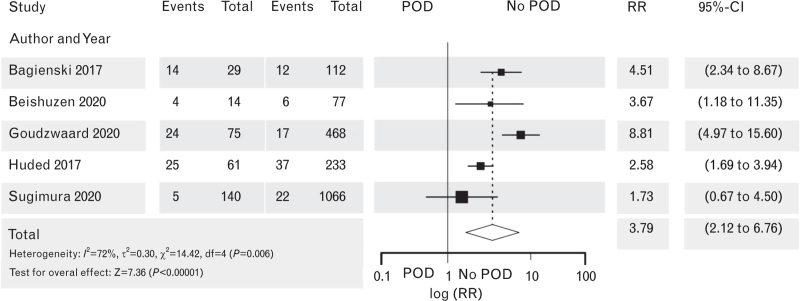
Forest plot for overall mortality in patients with postoperative delirium after cardiac surgery vs. patients with no postoperative delirium. The corresponding Funnel plot is shown in Supplement Figure S12.

**Fig. 10:**
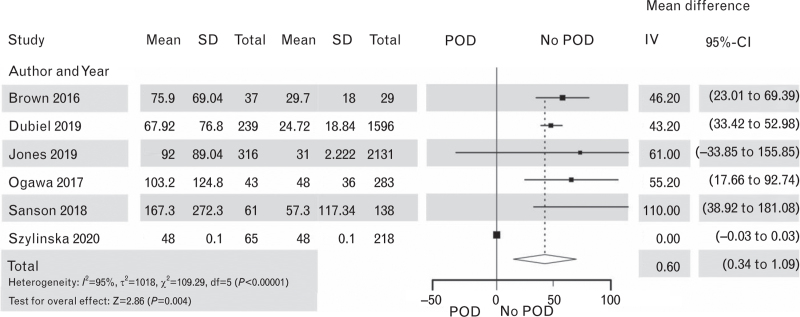
Forest plot for length of ICU stay in hours in patients with postoperative delirium after cardiac surgery vs. patients with no postoperative delirium. The corresponding Funnel plot is shown in Supplement Figure S13.

**Fig. 11:**
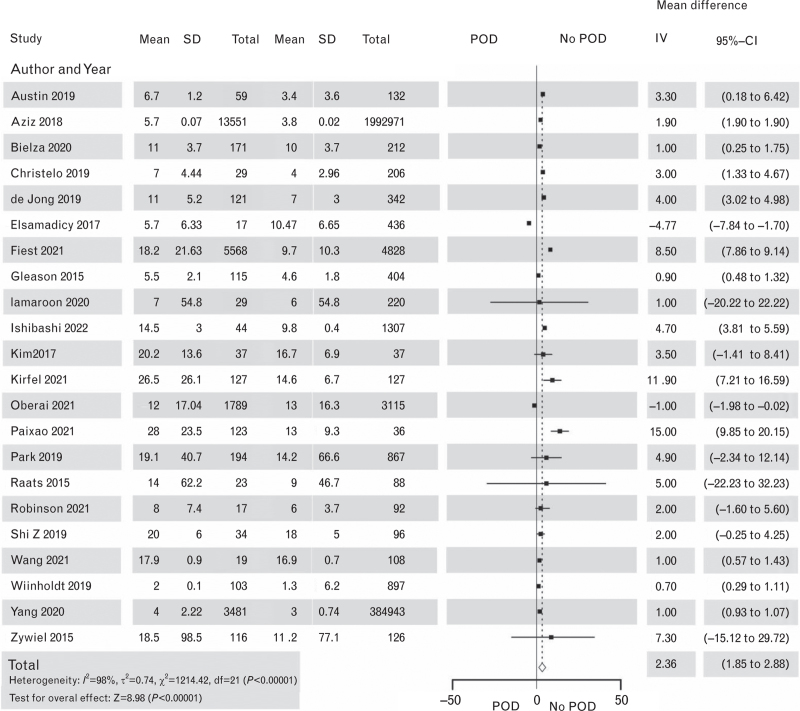
Forest plot for hospital length of stay in days in patients with postoperative delirium after noncardiac surgery vs. patients with no postoperative delirium. The corresponding Funnel plot is shown in Supplement Figure S14.

**Fig. 12:**
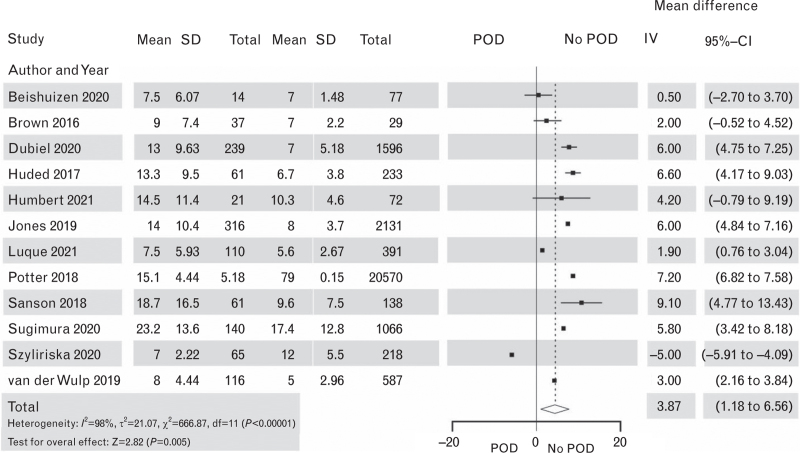
Forest plot for hospital length of stay in days in patients with postoperative delirium after cardiac surgery vs. patients with no postoperative delirium. The corresponding Funnel plot is shown in Supplement Figure S15.

Our systematic review of the studies on the treatment of POD and POD outcomes extracted from the broad search results (for details, see Supplement Figure S10) confirmed the findings of the first guideline version:^1^ patients suffering from POD have worse outcomes compared with patients who do not. POD had a significant impact on long-term mortality in noncardiac and cardiac surgery^158–173^ (Figs. 8 and 9, the corresponding Funnel plots are Supplement Figures S11 and S12). Surprisingly two of sixteen studies showed no decline in mortality associated with POD.^159,174^ POD further impacted on ICU length of stay (LoS) in cardiac surgery patients^175–179^ (Fig. 10 the corresponding Funnel plots is Supplement Figure S13) and hospital LoS for both cardiac and noncardiac surgical patients^167,168,174,180–189^ (Figs. 11 and 12, the corresponding Funnel plots are Supplement Figure S14 and S15). Overall, 12 vs. 9 RCTs showed a significant negative effect of POD on hospital LOS.

**Fig. 13:**
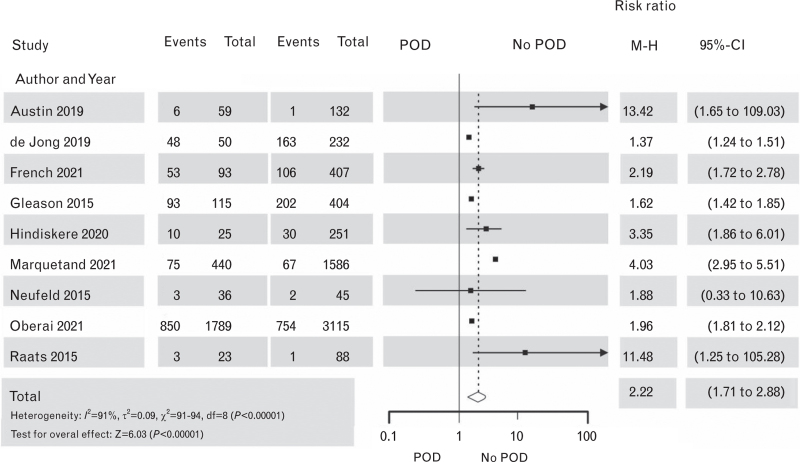
Forest plot of need for nursing care in patients with postoperative delirium after noncardiac surgery vs. patients with no postoperative delirium. The corresponding Funnel plot is shown in Supplement Figure S16.

POD led to higher costs.^163,175,189,190^ Meta-analysis of underlying health economics studies was not possible because of different currency and billing systems. The need for nursing care after hospital stay is more frequently required in POD patients compared with patients without POD^161,163,168,172,184,191–195^ (Fig. 13).

The objective of our working group was to find evidence-based information on different options of pharmacological interventions for POD, and to evaluate their efficacy as well as their harms and benefits. The first-line measures for prevention and treatment of POD are nonpharmacological. Only when there is severe and intractable distress should medications be used. For pharmacological therapy of POD, there are different treatment options available for the different delirium symptoms. For example, hallucinations can occur in patients with POD even when they are oriented, and if distressing they could be treated with antipsychotics, if nonpharmacological measures have failed.

When nonpharmacological measures have failed, a pharmacological treatment should be considered. Although there is little-to-no evidence from RCTs on the treatment of specific single symptoms, the following **symptom-oriented** treatment options are possible suggestions and should be carefully considered:

(1) Psychotic symptoms/hallucinations (using a validated monitoring such as the Questionnaire for Psychotic Experiences, QPE^196^): Neuroleptics [haloperidol, e.g. starting with 0.125 to 0.25 mg single dose, maximum dose per day less than 3 mg (mortality increased ≥ 6 mg day^−1^!), risperidone (starting with 0.125 mg single dose), olanzapine, quetiapine]
(2) Pain (using a validated monitoring such as NRS/VAS^197,198^ or BPS-NI/PAINAD^199^): opioid-based analgesia
(3) Day/night rhythm disorders (Richards–Campbell Sleep Questionnaire ^200^): melatonin
(4) Anxiety (FAS^201^): short-acting benzodiazepines with bolus-wise applications of low doses
(5) Agitation (Richmond Agitation and Sedation Scale, RASS):^202^ in ICU settings, alpha-2 agonists, for fluctuating symptoms, drugs with a short context-sensitive half-life (e.g. propofol)
(6) Vegetative symptoms (clinical appearance): in ICU settings, alpha-2 agonists, if necessary, beta-blockers to treat sinus tachycardia
(7) Delirium in the presence of alcohol withdrawal syndrome (diagnosis of exclusion, after considering all other diagnoses^203^): long-acting benzodiazepines (e.g. diazepam, lorazepam)

If POD is detected, patients should not be discharged from the recovery room to the ward without having started cause-based and symptom-based treatment. The longer the delirium lasts and the later the treatment starts, the more likely cognitive decline and worse clinical outcomes may be expected.^204^

Delirium in the presence of alcohol withdrawal syndrome is a special sub-form of delirium, which can occur in the perioperative setting as well, and it is challenging to differentiate the overlap with other forms of delirium.^205^ The diagnosis of delirium in the presence of alcohol withdrawal syndrome is a clinical diagnosis of exclusion, after considering all other diagnoses.^203^ Therapy of alcohol withdrawal delirium is based on long-acting benzodiazepines (e.g. diazepam, lorazepam).^203^

### PICO 16: Should antipsychotics be used for the treatment of POD?

**Table:** 

Recommendation 6.1	Quality of the Evidence	Strength of recommendation
We suggest using low-dose haloperidol for the treatment of POD if nonpharmacological measures fail. We advise a short-term, symptom-oriented therapy. The application should be bolus-wise and with the lowest dose possible. Use antipsychotic drugs with caution or not at all for people with preexisting neurologic conditions, such as Parkinson's disease or Lewy bodies dementia.	Very low RCT: Fukata et al., 2017 ^204^/SR: Shen *et al.*, 2018^207^	**Weak**

The evidence in support of the use of antipsychotics for POD treatment remains inconsistent and controversial. Postsurgical administration of haloperidol or atypical antipsychotics seems to alleviate POD and treat hallucinations as part of psychotic symptoms.^206,208–210^ It also showed several adverse effects, such as hypotension, sedation,^211^ extrapyramidal symptoms,^212^ and QT-prolongation.^206,209,210^ Most analysed randomised-controlled studies on the use of antipsychotics for POD treatment showed no positive effects on delirium overall. There was one systematic review with moderate certainty of the evidence, which included 12 RCTs on the treatment of POD with haloperidol and atypical antipsychotics and revealed no significant effect on delirium severity, delirium duration, mortality, hospital and ICU lengths of stay.^210^ It also concluded that low-dose haloperidol therapy has comparable efficacy and side effect rates to atypical neuroleptics.

We consider it necessary to emphasise that the studies included in the narrative synthesis were very heterogeneous, both in terms of the timing of drug application and the dosage of antipsychotics administered. They were often used for sedation and not strictly symptom-oriented, so neither harm nor benefit could be clearly proven.

Our analysis of five RCTs and two systematic reviews suggests that intravenous administration of haloperidol for the treatment of POD is still inconsistent but may slightly reduce the worsening of POD^206,209^ as well as the ICU LoS.^213^ Low-dose haloperidol demonstrated comparable efficacy and side effect rates to atypical neuroleptics,^207,210^ whereby the atypical neuroleptics showed a slightly lower incidence of adverse effects.^210^

In a systematic review from Neufeld *et al.*,^210^ antipsychotics for the treatment of delirium were analysed. They had no subgroup for POD, so that we could not adopt this review to our guideline because of a high proportion of nonsurgical patients suffering from delirium. In the included patients, collectively, no benefits of antipsychotic treatment for delirium was found.

Regarding the benefits and harms of haloperidol or other antipsychotics for delirium therapy, it is uncertain if there are clear benefits, and the risk of undesired side effects remains. Delirium is a complex disorder, and there is no single drug intervention that plausibly could treat all cases of delirium.^214^ Therefore, the concept of a drug treatment for delirium as a whole is flawed. However, in some circumstances, the use of antipsychotics might provide some benefit. There is no clear evidence for this, but expert consensus from published guidelines and standards supports limited use of antipsychotics only for severe distress particularly in the context of psychosis, and/or if hyperactivity and/or agitation is causing significant safety concerns when nonpharmacological interventions have been insufficiently effective or if the indication is urgent. We advise a short-term, symptom-oriented therapy. The application should be bolus-wise and with the lowest dose (suggestion on the initial dose in the elderly: 0.125 to 0.25 mg haloperidol): especially in elderly patients, who are considered being at particular risk of developing delirium after surgery and in which the sedative effect of antipsychotics predominates.

Antipsychotic drugs should be used with caution or not at all in patients with certain preexisting neurologic conditions, such as Parkinson's disease or Lewy bodies dementia.

### PICO 17: Should benzodiazepines be used for the treatment of POD?

**Table:** 

Recommendation 6.2	Quality of the evidence	Strength of recommendation
The use of benzodiazepines for the treatment of delirium in postoperative patients is **not** suggested. The evidence for the benefits of benzodiazepine therapy for treating POD symptoms or the underlying causes is very low to nonexistent. This recommendation is not to be confused with delirium in the context of alcohol withdrawal, where benzodiazepines are recommended symptom orientated as the first-line medication (in a bolus-titrated dosage, lowest as possible).	Very low Yapici *et al.*, 2011^215^	**Weak**

Benzodiazepines act at the γ-aminobutyric acid receptor (GABA-A) and mediate via anxiolytic, amnesic, sedative and anticonvulsant effects via various subunits. In some hospitals, benzodiazepines are still an integral part of sedation in ICUs and are used routinely to treat agitation and other symptoms. Dependence can occur even at therapeutic doses and carries the risk of developing withdrawal symptoms. Benzodiazepines often have active metabolites with a greater half-life than the basic substance itself, which increases the risk of accumulation, especially in patients with organ dysfunction requiring intensive care. Midazolam, in particular, is associated with a risk of accumulation because of its poor controllability. The certainty of the evidence for the use of benzodiazepines in treatment strategies for POD was very low to nonexistent. In the studies analysed here, there was only one RCT using benzodiazepines for the treatment of POD. Its efficacy was lower compared with alpha-2 agonists.^215^

Therefore, a symptom-orientated medication is essential in a bolus-titrated dosage, as low as possible.

### PICO 18: Should alpha-2 agonists be used for the treatment of POD?

**Table:** 

Recommendation 6.3	Quality of the evidence	Strength of recommendation
We suggest using dexmedetomidine for the treatment of postoperative delirium in cardiac surgery.	Very low RCT: Yapici *et al.*, 2011^215^/SR: Pieri *et al.*, 2019^216^ (based on Yapici 2011) RCT: Shokri and Ali, 2019^61^	**Weak**

The importance of alpha 2-adrenoceptor agonists has increased in recent years, both clinically and economically. There is evolving evidence of the beneficial use of alpha 2-adrenoceptor agonists, particularly in elderly patients with delirium, although the underlying pathological mechanisms are still uncertain. Clinically, these agents are primarily characterised by analgesic, sedative, anxiolytic and antihypertensive effects. They also lower sympathetic tone. In Europe, dexmedetomidine is approved by the EMA for mild-to-moderate sedation levels from Richmond Agitation and Sedation Scale (RASS) 0 to RASS -3. Deeper sedation with dexmedetomidine in younger adults is currently being discussed as being harmful in these patients, while it may be beneficial in older surgical patients.^217^

Regarding POD treatment studies with alpha 2-adrenoceptor agonists, there is little evidence. We analysed eight studies using dexmedetomidine in the perioperative context. Seven of the eight studies were in cardiac surgery patients, including one systematic review. Therefore, no recommendation on alpha 2-adrenoceptor agonists for delirium treatment in noncardiac surgical patients is possible. Some of the studies included in our analysis overlapped with the studies included in the chapter on pharmacologic prevention of POD. In some study protocols, the investigated drug continued to be used even after the onset of POD. Thus, like the authors of the section on pharmacologic prevention of POD, the authors of the present section made the judgement that it was reasonable to draw some conclusions about the therapeutic effectiveness from these studies.^61^ We chose delirium duration as our outcome for therapeutic effectiveness, and we rated delirium incidence as a preventive effect, when the investigational drug was started before delirium occurred.

Certainty in reduction of severity of POD was rated from very low to low because of serious risk of bias: not all studies fulfilling POD assessment criteria included in the meta-analysis distinguished between POD and ICU delirium in postoperative patients, nor between preventive and treatment effects of dexmedetomidine. Two of the eight analysed studies lacked blinding of the participating physicians.

There is moderate certainty of evidence that postoperative patients receiving dexmedetomidine may lower delirium duration^57,59,61^ and also mortality rates compared with patients receiving placebo, propofol or clonidine infusion.^42,61^ The heterogeneity of the studies made meta-analysis inappropriate (Supplement Figure S17). There is low-to-moderate certainty of evidence that postoperative administration of dexmedetomidine is likely to reduce time to extubation,^42,53,215^ hospital LoS,^42,57,61^ ICU LoS^42,61^ and time to onset of delirium.^57^ The main limitations in all studies analysed were considerable imprecision (small sample sizes) and significant indirectness (i.e. mixed ICU and surgical patients, different timing or dosage of dexmedetomidine administration). As only one RCT used another alpha 2-adrenoceptor agonist (clonidine) as a comparator to dexmedetomidine,^61^ the panel decided to use ‘dexmedetomidine’ in the suggestion.

Another concern with the use of alpha 2 agonists for POD treatment is their dependence on availability in different hospitals. Dexmedetomidine appears to be more expensive, but it is associated with significant cost reduction because of shorter ICU/hospital stays.^218^ The trials were all conducted in a hospital setting. The use of dexmedetomidine for POD treatment was only recommended for cardiac surgery patients, as most of the studies included in the analyses focused on this patient population.

#### Melatonin (no recommendation due to *severe bias*)

Three RCTs from 2021 were included in the final analysis. All studies investigated the effect of melatonin on POD.^63,219,220^ Due to the small number of studies, their serious indirectness and imprecision, and resulting low to very low grade of evidence, no recommendations could be made for the use of melatonin in the treatment of POD. The panel discussed the matter and voted unanimously.

#### Other medications (no recommendation due to *insufficient data*)

No recommendations could be made for the use of other drugs (namely pregabalin, gabapentin, rosuvastatin and morphine sulphate)^68,211,221^ for POD therapy in a hospital setting due to the lack of adequate studies (only one study of each drug fulfilling our POD criteria).

## Discussion

Despite the enormous amount of newly published research results on POD, significant changes in recommendations regarding either prevention or treatment are absent. Healthcare resources are almost exclusively absorbed by curative strategies. The prevention of POD requires multicomponent strategies delivered by multidisciplinary teams and ideally starting weeks before an elective surgery. No two older patients with planned (or unplanned) surgery will have the same risk profile and will, thus, equally profit from a standardised prevention plan. However, such standardised strategies are necessary to allow the common evidence-based pathways from RCTs to evidence-to-decision-based recommendations and suggestions. This seems appropriate in RCTs on anaesthetic drugs where randomisation and (even triple) blinding is easily feasible.

However, in studies on neuromonitoring or in studies on nonpharmacologic multicomponent interventions, blinding the study personnel is much more challenging. Additionally, multicomponent strategies usually offer a set of different treatment options, which are tailored to the specific risk profile of the single patient. Thus, within a single study, not all participants in the intervention group receive the same set of interventions, and not all participants even receive all the interventions they were allocated.^98^ This makes it difficult to correctly judge the efficacy of certain approaches. It makes it even more difficult to pool evidence from different studies on multicomponent interventions. It is, however, obvious that there is no ‘one-size-fits-it-all’ solution, which can be recommended. Pre-post study designs could overcome some of these problems. However, when examining the many clinical or registry-based studies using a pre-post study design, it turned out that often participants in the intervention period of the study were prospectively evaluated for POD, while diagnoses of POD in control patients were derived from retrospective chart reviews or discharge letters. Even the prospective assessment of POD substantially varied between studies, which hampered pooling of study results.

Substantial improvements in study design and measurement methodologies are warranted. For the future, it is highly recommended that assessments focus on either the reference standard DSM-5-based definition of POD, or on tools that are validated against a reference standard. This means using a validated reference standard method or tool suitable for the setting and the patient population.^214^ Considering the fluctuating course of POD during the day, the Task Force and Advisory board of this guideline further recommends for all future POD studies to start screening for POD in the recovery room. The screening for POD should be continued at least until day 3 after surgery and at least twice a day. In addition, the suspected underlying medical reason for POD is important to describe and should additionally be documented for each POD assessment.

Apart from study data, it is also desirable to rely more on structured routine data annotated in a register-based format with an internationally accepted and agreed terminology, such as the Systematized Nomenclature of Medicine (SNOMED)^222^ from the International Health Terminology Standards Development Organisation (IHTSDO). Then artificial intelligence-based algorithms could be developed to evaluate the effectiveness of certain interventions in routine clinical practice more reliably. As a minimum, it requires an identical assessment of POD before and after an (newly implemented) intervention and a set of standard variables to characterise the patient before surgery and agreement on how to measure (in addition to POD) other important clinically and patient-related outcomes.

For precipitating factors associated with anaesthesia and surgery, it is relevant to monitor cerebral effects of anaesthesia through neuromonitoring on a level that considers drug-specific and patient-specific patterns as is already done during monitoring of circulation parameters. Based on adequate staff training, the anaesthesiologists should likely avoid burst suppression in their patients. In addition, other causes relevant to surgery such as inflammation, circulation-based hypoxic states, and haemodynamic instabilities require clinical trials to demonstrate effective interventions.

For both ERAS-inspired approaches combining nonpharmacological and pharmacological treatments, future studies should holistically integrate elements of personalised medicine from genetic makeup, molecular mechanisms, clinical phenomenology to subjective feelings and views of patients and their relatives. Patients’ needs should be adequately addressed through a 360° appraisal. Delirium intervention trials have mostly used the blunt approach of considering delirium as a binary outcome; future studies should allow for studying the effects of interventions on particular symptoms of delirium such as distress and psychosis to better enable targeting of treatments. Finally, it will also be beneficial to better understand positive trajectories, when POD does not occur and also when patients not only recover to their presurgical status but also clearly benefit from their surgical and anaesthesiological treatment. Further advances in anaesthesia and surgery techniques as well as in geriatric peri-operative care will decrease or even eliminate the risk of negative cognitive trajectories after geriatric surgery.

## Executive summary

Life expectancy is still increasing and a 65-year-old European has around 20 more years to live.^223^ The need for surgery (and the accompanying need for anaesthesia) rises with increasing age.^224,225^ Consequently, over the next decades, surgery-related and anaesthesia-related complications such as POD will increase in absolute numbers. To reduce both the individual and the societal burden of POD and its long-term sequelae, preventive strategies are necessary. This requires knowledge, understanding, training and intention on the part of the entire peri-operative and postoperative team. Implementation of dedicated pathways for the prevention, screening and eventual treatment of POD in the clinical routine is urgently warranted. Such implementation concerns the evaluation of preoperative risk factors for POD (recommendation 2.1), the use of prophylactic drugs, biomarkers, type of surgery or anaesthesia (recommendations 3.1 to 3.4) and the implementation of nonpharmacological interventions (recommendations 4.1 to 4.3). Neuromonitoring (recommendations 5.1 and 5.2) plays a crucial role in the prevention of POD. If POD is not avoidable and nonpharmacological measures fail, pharmacological treatment options exist (recommendations 6.1 to 6.3). Research on POD has strongly intensified within the last decade, and findings on basic pathophysiological mechanisms will continue to emerge over the next decade, thus crystallising or revising current understanding. As with all rapidly developing fields, current conclusions will be subject to revision in years to come.

**Table:** 

Recommendation 2.1	Quality of the evidence	Strength of recommendation
We recommend evaluating the following preoperative risk factors for POD: (1) older age, (2) American Society of Anesthesiology Physical status score > 2, (3) Charlson Comorbidity Index ≥2 and (4) Mini Mental State Examination score lower than 25 points	Moderate	**Strong**

**Table:** 

Recommendation 3.1	Quality of the evidence	Strength of recommendation
In patients undergoing surgery, we do **not** suggest the use of any drug as a prophylactic measure to reduce the incidence of POD.	Low	**Weak**

**Table:** 

Recommendation 3.2	Quality of the evidence	Strength of recommendation
When dexmedetomidine is used intra-operatively or postoperatively with the aim to prevent POD, we recommend balancing the expected benefits against the most important side effects (bradycardia and hypotension).	Moderate	**Strong**

**Table:** 

Recommendation 3.3	Quality of the evidence	Strength of recommendation
In patients undergoing surgery, we do not suggest any specific type of surgery or type of anaesthesia to reduce the incidence of POD.	Low	**Weak**

**Table:** 

Recommendation 3.4	Quality of the evidence	Strength of recommendation
We do not suggest using biomarkers to identify patients at risk of POD.	Low	**Weak**

**Table:** 

Recommendation 4.1	Quality of the evidence	Strength of recommendation
We recommend that preoperative anaesthesia consultation in older adults includes the screening for risk factors for POD and addresses patients’ needs to optimise their preoperative status.	Low	**Strong**

**Table:** 

Recommendation 4.2	Quality of the evidence	Strength of recommendation
We recommend that the results of the screening for POD risk factors are shared among the care team and the preventive strategies discussed and registered in the medical records.	Low	**Strong**

**Table:** 

Recommendation 4.3	Quality of the evidence	Strength of recommendation
We recommend multicomponent nonpharmacological interventions in all patients at risk of POD.	Moderate	**Strong**

**Table:** 

Recommendation 5.1	Quality of the evidence	Strength of recommendation
We suggest Index-based EEG-monitoring depth of anaesthesia guidance to decrease the risk of POD.	Low	**Weak**

**Table:** 

Recommendation 5.2	Quality of the evidence	Strength of recommendation
We suggest multiparameter, intraoperative EEG monitoring (burst suppression, density spectral array, DSA) during anaesthesia to decrease the risk of POD.	Low	**Weak**

**Table:** 

Recommendation 6.1	Quality of the evidence	Strength of recommendation
We suggest using low-dose haloperidol for the treatment of POD if nonpharmacological measures fail. We advise a short-term, symptom-oriented therapy. The application should be bolus-wise and with the lowest dose possible. Use antipsychotic drugs with caution or not at all for people with preexisting neurologic conditions, such as Parkinson's disease or Lewy bodies dementia.	Very low	**Weak**

**Table:** 

Recommendation 6.2	Quality of the evidence	Strength of recommendation
The use of benzodiazepines for the treatment of delirium in postoperative patients is not suggested. The evidence for the benefits of benzodiazepine therapy for treating POD symptoms or the underlying causes is very low to nonexistent. This recommendation is not to be confused with delirium in the context of alcohol withdrawal, where benzodiazepines are recommended symptom orientated as the first-line medication (in a bolus-titrated dose, lowest as possible).	Very low	**Weak**

**Table:** 

Recommendation 6.3	Quality of the evidence	Strength of recommendation
We suggest using dexmedetomidine for the treatment of POD in cardiac surgery.	Very low	**Weak**

## Supplementary Material

Supplemental Digital Content
