# Supplementary material for: Update of the European Society of Anaesthesiology and Intensive Care Medicine evidence-based and consensus-based guideline on postoperative delirium in adult patients
Source: Eur J Anaesthesiol. 2023 Aug 30;41(2):81–108. doi: 10.1097/EJA.0000000000001876 (PMC10763721; doi:10.1097/EJA.0000000000001876)
Supplement: Supplemental Digital Content [file ejanet-41-081-s001.docx]

Supplementary Materials for

**Update of the European Society of Anaesthesiology and Intensive Care Medicine (ESAIC) evidence-based and consensus-based guideline on postoperative delirium in adult patients**

Aldecoa C, Bettelli G, Bilotta F, Sanders RD, Spies CD* et al.

*Corresponding author. Email: claudia.spies[@charite.de](mailto:xxxxx@xxxx.xxx)

**General approach for all working groups:**

| Basic science | Publications with animal models or cell cultures mentioning the word ‘delir’ or ‘delirium’ within title or abstract |
| --- | --- |
| Risk factors | Publications presenting an univariable or multivariable model on 'factors associated with POD', or 'risk factors for POD' or 'variables explaining POD' etc. If a study group was stratified in e.g. ‘elderly’ and ‘super-elderly’ and the abstract mentioned differences in the POD incidence between these two age-groups, this study was regarded as potentially relevant for the RF working group too. If intervention studies evaluated risk factors for POD incidence, these studies were regarded as potentially relevant for the RF working group too. |
| Preventive Measures 1 | Publications evaluating anesthesiologic or surgical procedures and interventions regarding POD incidence. Additionally, studies evaluating biomarkers or markers of neuro-imaging, and studies evaluating diagnostic tests were selected for this working group. |
| Preventive Measures 2 | Publications evaluating complex geriatric +/- surgical non-pharmacologic interventions in patients, including add-on CGA [+ risk factor modification], interventions in caregivers, interventions on hospital level were included here. Likewise, surveys among health care providers or qualitative research in patients and relatives on attitudes towards and experiences with POD were included here. |
| Neuromonitoring | Publications evaluating processed EEG / EEG parameter before or after surgery (in combination with POD as the outcome) were included here. |
| Treatment & Outcomes | Publications evaluating any kind of outcomes (in pts with or without POD) were included here. This was typically length of (ICU or overall hospital) stay, post-op complications, mortality, but likewise quality of life, institutionalization, costs, post-op health care utilization etc. We additionally included studies, when POD was ‘only’ regarded as a risk factor for further complications or any ‘general outcome’. |

Supplement Table S1: Algorithm for the assignment to the working groups


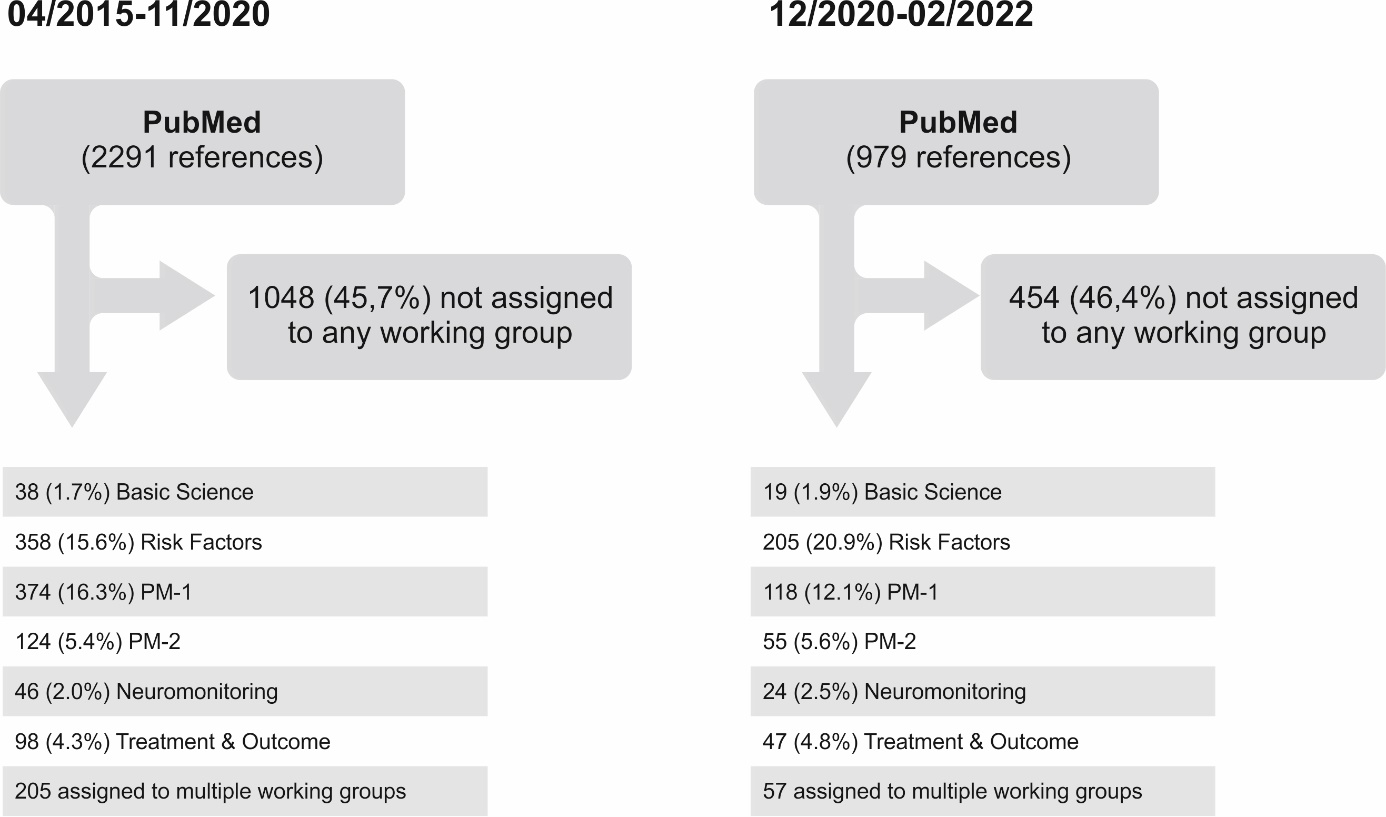
Supplement Figure S1: Assignment of potentially relevant studies to the six working groups, April 2015 until February 2022 Supplement

| Screening tool | Validation studies in postsurgical patients |
| --- | --- |
| 3D-CAM | Olbert M et al. (2019) Validation of 3-minute diagnostic interview for CAM-defined delirium to detect postoperative delirium in the recovery room: a prospective diagnostic study. Eur J Anaesthesiol 36:683–687 |
| 4AT | Saller T et al. (2019) Screening for delirium after surgery: validation of the 4 A’s test (4AT) in the post-anaesthesia care unit. Anaesthesia 74:1260–1266 |
| CAM | Inouye SK et al. (1990) Clarifying confusion: the confusion assessment method. Ann Intern Med. 1990; 113: 941-948.  Radtke FM et al (2008) Comparison of three scores to screen for delirium in the recovery room. Br J Anaesth; 101:338–343.  Smulter N et al (2015). Validation of the Confusion Assessment Method in Detecting Postoperative Delirium in Cardiac Surgery Patients. Am J Crit Care; 24: 480–487. |
| CAM-ICU | Ely EW et al. (2001) Delirium in mechanically ventilated patients: validity and reliability of the confusion assessment method for the intensive care unit (CAM-ICU). JAMA; 286:2703-10  Neufeld KJ et al (2013) Evaluation of two delirium screening tools for detecting post-operative delirium in the elderly. Br J Anaesth; 111:612–618 |
| CAM-S^§^ | Inouye SK et al. (2014) The CAM-S: development and validation of a new scoring system for delirium severity in 2 cohorts. Ann Intern Med; 160:526-53 |
| Confusional State Examination^§^ | Robertsson B et al. (1997) Confusional State Evaluation (CSE): an instrument for measuring severity of delirium in the elderly. Br J Psychiatry; 170:565-570. |
| DDS | Radtke FM et al (2008) Comparison of three scores to screen for delirium in the recovery room. Br J Anaesth; 101:338–343.  Radtke FM et al (2010) A Comparison of Three Scores to Screen for Delirium on the Surgical Ward. World J Surg; 34:487–494. |
| Delirium-O-Meter^§^ | De Jonghe et al. (2005) Delirium-O-Meter: a nurses' rating scale for monitoring delirium severity in geriatric patients. Int J Geriatr Psychiatry; 20:1158-66 |
| DOS^§^ | Schuurmans MJ et al. (2003) The Delirium Observation Screening Scale: a screening instrument for delirium. Res Theory Nurs Pract; 17:31-50.  Koster S et al. (2009) The delirium observation screening scale recognizes delirium early after cardiac surgery. Eur J Cardiovasc Nurs; 8:309-14.  Scheffer AC et al. (2011): Assessing severity of delirium by the Delirium Observation Screening Scale. Int J Geriatr Psychiatry; 26: 284–91.  Park J et al. (2021) The Delirium Observation Screening Scale: A Systematic Review and Meta-Analysis of Diagnostic Test Accuracy. Clin Nurs Res; 30:464-473. |
| DRS-R-98^§^ | Trzepacz P T et al. (2001) Validation of the Delirium Rating Scale-revised-98: comparison with the delirium rating scale and the cognitive test for delirium. J Neuropsychiatry Clin Neurosci; 13:229-42. |
| ICDSC | Bergeron N et al. (2001) Intensive Care Delirium Screening Checklist: evaluation of a new screening tool. Intensive Care Med; 27:859–64. |
| MDAS^§^ | Breitbart W et al. (1997) The Memorial Delirium Assessment Scale. Journal of Pain Symptom Management; 13:128–137.  Marcantonio E et al. (2002) Delirium severity and psychomotor types: their relationship with outcomes after hip fracture repair. J Am Geriatr Soc; 50:850-7. (hip fracture surgery only)  Kazmierski J et al. (2008) Clinical Utility and Use of DSM–IV and ICD–10 Criteria and The Memorial Delirium Assessment Scale in Establishing a Diagnosis of Delirium After Cardiac Surgery. Psychosomatics; 49: 73-76. |
| NEECHAM | Duppils G & Johansson I. (2011) Predictive value and validation of the NEECHAM Confusion Scale using DSM-IV criteria for delirium as gold standard. Int J Older People Nurs; 6:133-42. |
| NuDesc | Neufeld KJ et al (2013) Evaluation of two delirium screening tools for detecting post-operative delirium in the elderly. Br J Anaesth 111:612–618.  Vasunilashorn SM et al. (2016) Derivation and Validation of a Severity Scoring Method for the 3-Minute Diagnostic Interview for Confusion Assessment Method--Defined Delirium. J Am Geriatr Soc; 64:1684-9. |
| OBS | Eriksson M et al. (2002) Delirium after coronary bypass surgery evaluated by the organic brain syndrome protocol. Scand Cardiovasc J; 36:250-5. |

§ = Tests selected as high-quality instruments assessing delirium severity^1^; 4AT = 4 ‘A’s test; CAM = Confusion Assessment Method; DDS = Delirium Detection Score; DOS = Delirium Observational Scale; DRS-R-98 = Delirium Rating Scale, revised 1998; ICDSC = Intensive Care Delirium Screening Checklist; MDAS = Memorial Delirium Assessment Scale; NuDesc = Nursing Delirium Screening Scale; OBS = Organic Brain Syndrome Scale

Supplement Table S2: List of validated screening tools for postoperative delirium

**Specific approaches of the different working groups:**

**Supplement Chapter 1: Basic Science**

*Authors: Colm Cunningham, Rob Sanders, Bjoern Weiss*

No supplementary material.

**Supplement Chapter 2: Risk Factors**

*Authors:* Federico Bilotta, Ali Forookhi, Henrik Kehlet, Lior Mevorach, Stefano Romagnoli


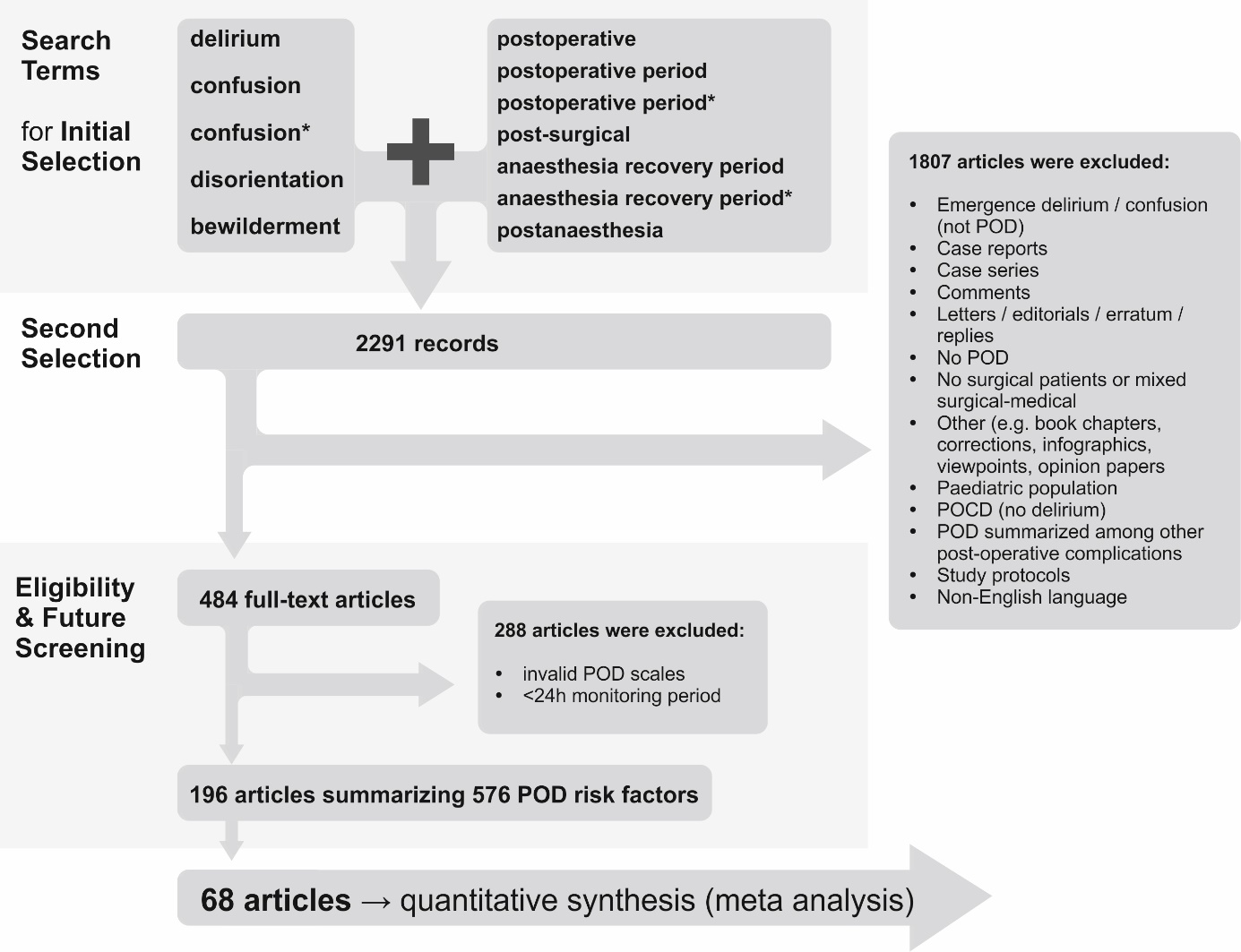


Supplement Figure S2: Flow chart of the Risk Factors (RF) working group literature search procedure from 01-04-2015 to 31-11-2020.

**Supplement Chapter 3: Preventive Measures I: Effects of Drugs on POD prevention**

Authors: Katarzyna Kotfis, Annika Reintam Blaser, Antonio Cherubini, Wojciech Dabrowski, Nicola Latronico, Simone Piva, Nicola Gitti, Bruno Neuner, Stefania Renzi

The Preventive Measures I working group used the potentially relevant studies identified with the broad literature searches (see Figures 1.1 and 1.2 in the main document) as well as results from a specific literature search on Dexmedetomidine. The title and abstract review of the potentially relevant studies was performed independently by two members of the group to identify studies (randomized controlled trials, before/after studies with prospective controls, cross-sectional, case-control, and cohort studies) assessing one of the pre-defined PICO questions.

Identification of papers for full-text assessment was based on inclusion and exclusion criteria as described in the general approach for all working groups. The full-text assessment was performed using the following specific information: a. two subgroups were used - cardiac and non-cardiac; b. studies were divided into categories: systematic reviews, RCTs, observational studies assessing interventions; c. four phases were evaluated: preoperative, intraoperative, postoperative, and perioperative (combination of previous). Existing SR/MA were assessed separately to A) identify available SR/MA on the pre-defined PICO & performed based on the inclusion criteria; B) identify potentially eligible studies that were not identified during our search. A narrative summary of identified evidence includes results from systematic reviews.

Three areas of preventive measures: medications, anesthesia, and surgical procedures, as well as biomarkers served to create 12 PICO questions (all with P - patients and O - outcome as described above and I – intervention, C - control):

1. Dexmedetomidine: I – Preventive use of dexmedetomidine pre-, intra, or postoperatively vs. C – non-dexmedetomidine/placebo.

2. Neuroleptics: I – Preventive use of neuroleptics pre-, intra, or postoperatively vs. C – non-neuroleptics/placebo.

3. Sleep medications (melatonin, ramelteon): I – Preventive use of sleep medications pre- or postoperatively vs. C – no sleep medications/placebo.

4. Cholinesterase inhibitors: I – Preventive use of cholinesterase inhibitors pre- or postoperatively vs. C – no use of cholinesterase inhibitors.

5. Other drugs: I – Application of a drug to reduce POD vs. C – no application of any specific drug to reduce POD.

6. Anesthetic drugs I: I – Intravenous anesthetics vs. C – inhalational anesthetics.

7. Anesthetic drugs II: I – Ketamine intra or postoperatively vs. C – no ketamine.

8. Type of Anesthesia: I – Regional anesthesia vs. C – general anesthesia.

9. Surgery: I – Minimally invasive surgery vs. C – more invasive surgery.

10. Surgery: I – Laparoscopy vs. C – laparotomy.

11. Cardiac Surgery: I – Off-pump cardiac surgery vs. C – on-pump cardiac surgery.

12. Biomarkers: I/E (exposure) – Abnormal value of a biomarker pre-, intra, or postoperatively vs. C – normal level of a biomarker.

**PICO 1 (Preventive use of dexmedetomidine pre-, intra, or postoperatively vs. non-dexmedetomidine/placebo).**

**PICO 1A: Dexmedetomidine vs. placebo**

**PICO 1B: Dexmedetomidine vs. other drug**

We included 22 RCTs in our analyses, 17 of them comparing dexmedetomidine vs. placebo (PICO 1A) and 5 comparing dexmedetomidine vs. another drug. One of the studies used clonidine as comparison, therefore, a sensitivity analysis with the exclusion of this study was performed. For both PICO 1A and PICO 1B subgroup analysis for cardiac and non-cardiac surgery was performed.

**
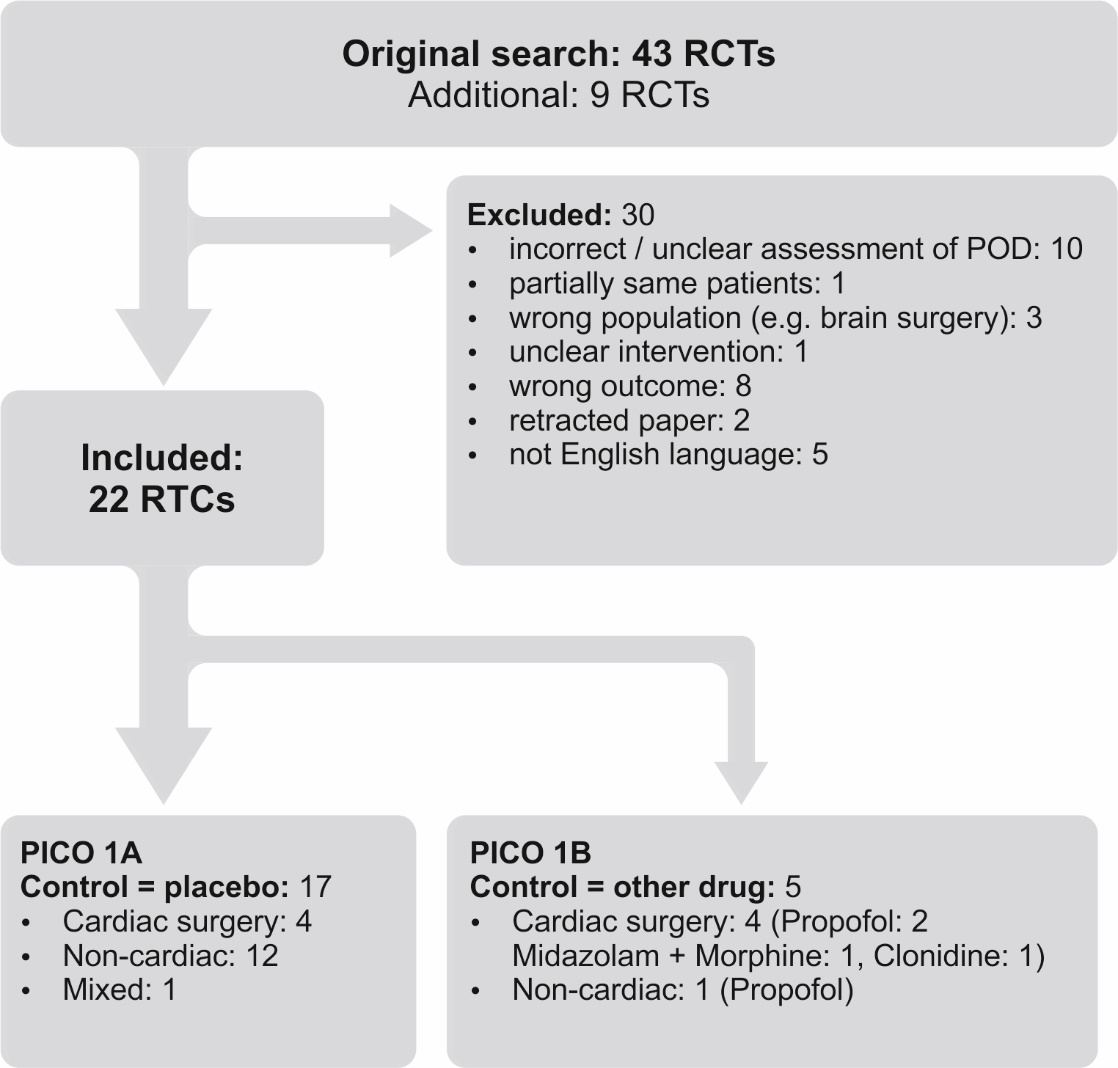
**

POD = postoperative delirium; RCT = randomized controlled trial; * = additional RCTs were identified during assessment of the systematic reviews (n=34) and related articles in PubMed.

Supplement Figure S3: Flow-chart of the Literature Search procedure of the Preventive measures I working group

Most of the studies included patients aged >60 years (18 studies). Common exclusion criteria in the included studies were ASA class >III; bradycardia <50 bpm and/or cardiac insufficiency, and liver and renal dysfunction. Included studies applied dexmedetomidine intra- and/or postoperatively but not preoperatively.

| **PICO 1** | |  | | | | | | | | | | |
| --- | --- | --- | --- | --- | --- | --- | --- | --- | --- | --- | --- | --- |
| **Question 1A** | | **Dexmedetomidine vs. placebo** | | | | | | | | | | |
| **Quality assessment** | | | | | | | **№ of patients** | | **Effect** | | **Quality** | **Importance** |
| **№ of studies** | **Study design** | **Risk of bias** | **Inconsistency** | **Indirectness** | **Imprecision** | **Other considerations** | **DEX** | **Placebo** | **Relative**  **(95% CI)** | **Absolute**  **(95% CI)** |  |  |
| Postoperative delirium | | | | | | | | | | | | |
| 17 | randomised trials | not serious | serious ^1^ | serious ^2^ | not serious ^3^ | none | 306/2784 (11.0%) | 429/2695 (15.9%) | **RR 0.64**  (0.50 to 0.82) | **62 fewer per 1,000**  (from 27 fewer to 96 fewer) | ⨁⨁◯◯  LOW | CRITICAL |
| Bradycardia | | | | | | | | | | | | |
| 9 | randomised trials | not serious | not serious | serious ^2^ | not serious | none | 270/2504 (10.8%) | 187/2514 (7.4%) | **RR 1.43**  (1.20 to 1.69) | **32 more per 1,000**  (from 1 more to 63 more) | ⨁⨁⨁◯  MODERATE | CRITICAL |
| Comments:   1. We downgraded the quality of evidence for inconsistency, I2=62% 2. We downgraded the quality of evidence for indirectness (selected study population) 3. We did not downgrade for imprecision despite the CI in cardiac subgroup included both benefit and harm, because we considered this aspect also included under inconsistency | | | | | | | | | | | | |

Supplement Table S3. Evidence profile for dexmedetomidine vs. placebo.

| **PICO 1** | |  | | | | | | | | | | | |
| --- | --- | --- | --- | --- | --- | --- | --- | --- | --- | --- | --- | --- | --- |
| **Question 1B** | | **Dexmedetomidine vs. other drugs** | | | | | | | | | | | |
| **Quality assessment** | | | | | | | **№ of patients** | | **Effect** | | **Quality** | **Importance** | |
| **№ of studies** | **Study design** | **Risk of bias** | **Inconsistency** | **Indirectness** | **Imprecision** | **Other considerations** | **DEX** | **Placebo** | **Relative**  **(95% CI)** | **Absolute**  **(95% CI)** |  |  |  |
| Postoperative delirium | | | | | | | | | | | | | |
| 4 | randomised trials | not serious | not serious | serious ^1^ | serious ^2^ | none | 86/625 (13.8%) | 130/619 (21.0%) | 0.66  (0.51 to 0.85) | **68 fewer per 1,000**  (from 28 more to 108 fewer) | ⨁⨁◯◯  LOW | CRITICAL |  |
| Bradycardia | | | | | | | | | | | | | |
| 1 | randomised trials | not serious | serious ^3^ | serious ^1^ | not serious | none | 16/84 (19.0%) | 0/85 (0.0%) | 33.4  (2.03 to 547.7) | **191 more per 1,000**  (from 104 more to 277 more) | ⨁⨁◯◯  LOW | CRITICAL |  |
| Comments:   1. We downgraded the quality of evidence for indirectness (selected study population) 2. We downgraded the quality of evidence for imprecision by one level, the CI included significant benefit and minimal harm | | | | | | | | | | | | | |

Supplement Table S4: Evidence profile for dexmedetomodine vs. other drug.

Meta-analyses on adverse effects of Dexmedetomidine:


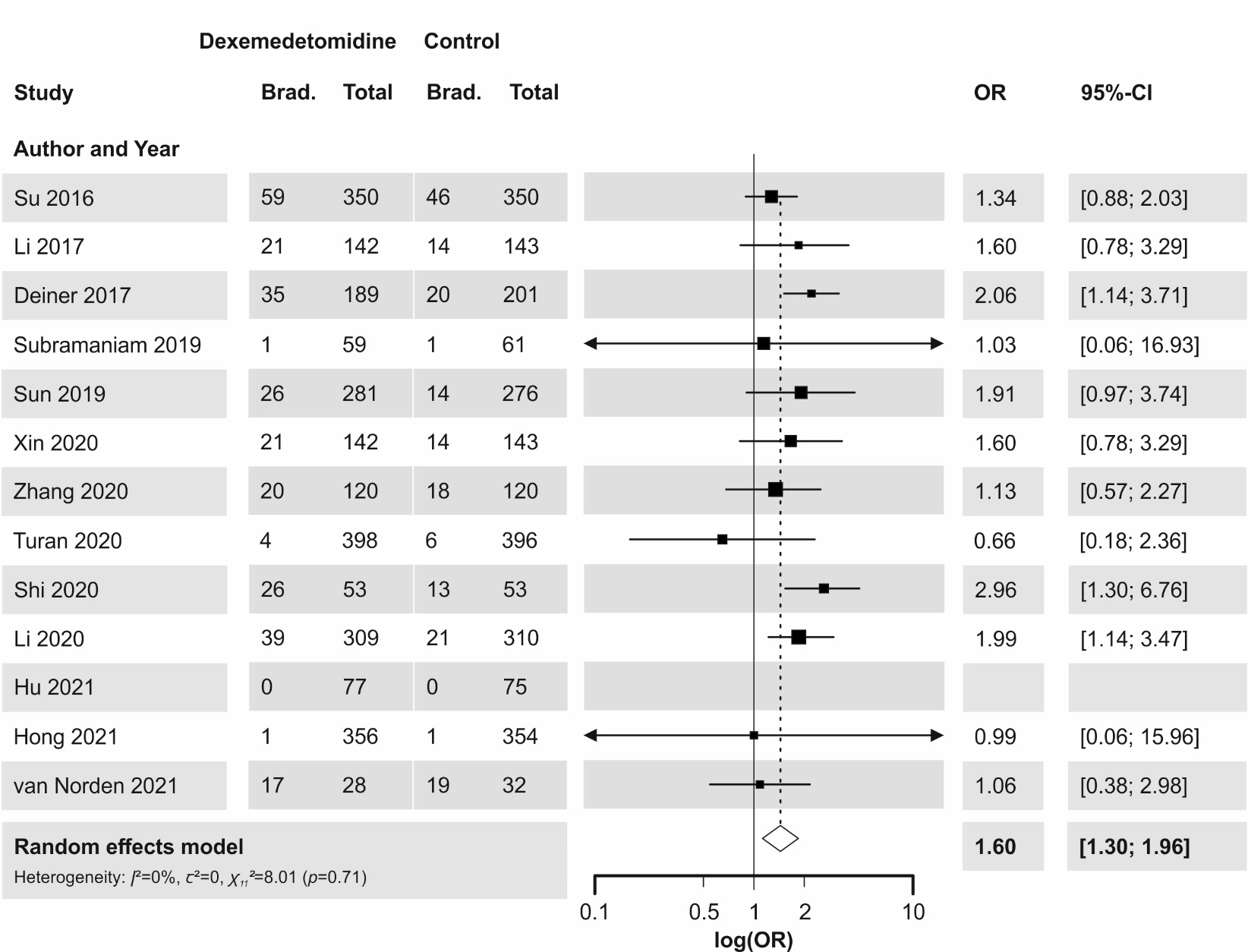


Supplement Figure S4: Forest plot for bradycardia outcome dexmedetomidine vs. any control (placebo or other drug).


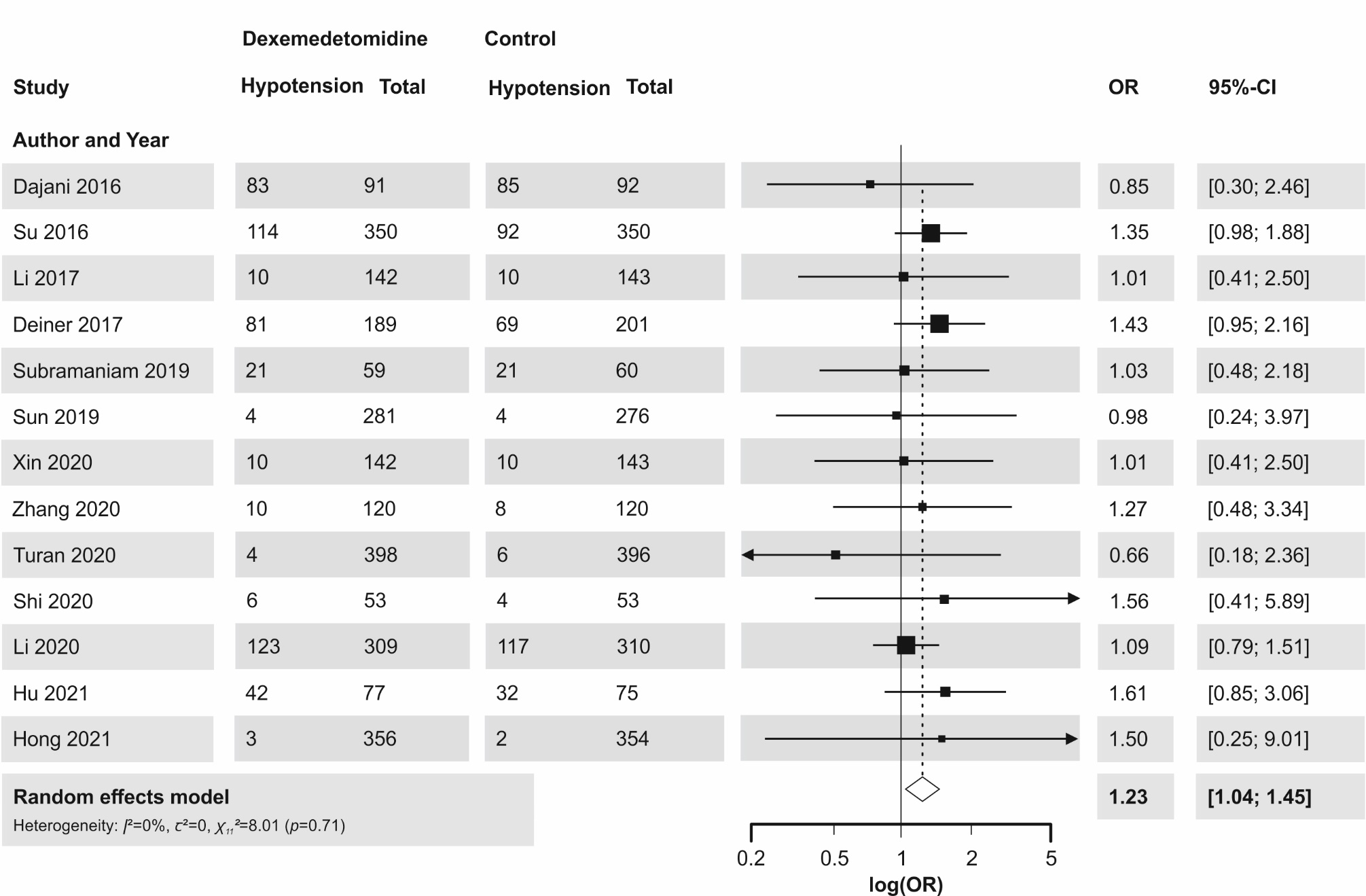


Supplement Figure S5: Forest plot for hypotension outcome dexmedetomidine vs. any control (placebo or other drug).

|  | | | |
| --- | --- | --- | --- |
| Should **Dexmedetomidine be used** (intra- or postoperatively) to reduce delirium in postoperative patients? | | | |
| Population: | Surgical patients | Background: |  |
| Intervention: | Dexmedetomidine (any dosage intra- or postoperatively) |  |  |
| Comparison: | Placebo |  |  |
| Main outcomes: | POD based on our definition |  |  |
| Setting: | Postoperative care |  |  |
| Perspective: |  |  |  |

Supplement Table S5: Evidence-to-Decision based on GRADE.

|  | **JUDGEMENT** | **RESEARCH EVIDENCE** | **ADDITIONAL CONSIDERATIONS** |
| --- | --- | --- | --- |
| PROBLEM | **Is the problem a priority?**  ○ No  ○ Probably no  ○ Probably yes  ● Yes    ○ Varies  ○ Don't know |  |  |
| DESIRABLE EFFECTS | **How substantial are the desirable anticipated effects?**  ○ Trivial  ○ Small  ● Moderate  ○ Large    ○ Varies  ○ Don't know |  |  |
| UNDESIRABLE EFFECTS | **How substantial are the undesirable anticipated effects?**  ○ Large  ● Moderate  ○ Small  ○ Trivial    ○ Varies  ○ Don't know |  |  |
| CERTAINTY OF EVIDENCE | **What is the overall certainty of the evidence of effects?**  ○ Very low  ● Low  ○ Moderate  ○ High    ○ No included studies |  |  |
| VALUES | **Is there important uncertainty about or variability in how much people value the main outcomes?**  ○ Important uncertainty or variability  ● Possibly important uncertainty or variability  ○ Probably no important uncertainty or variability  ○ No important uncertainty or variability    ○ No known undesirable outcomes |  |  |
| BALANCE OF EFFECTS | **Does the balance between desirable and undesirable effects favor the intervention or the comparison?**  ○ Favors the comparison  ○ Probably favors the comparison  ● Does not favor either the intervention or the comparison  ○ Probably favors the intervention  ○ Favors the intervention    ○ Varies  ○ Don't know |  |  |
| RESOURCES REQUIRED | **How large are the resource requirements (costs)?**  ○ Large costs  ● Moderate costs  ○ Negligible costs and savings  ○ Moderate savings  ○ Large savings    ○ Varies  ○ Don't know |  |  |
| CERTAINTY OF EVIDENCE OF REQUIRED RESOURCES | **What is the certainty of the evidence of resource requirements (costs)?**  ○ Very low  ● Low  ○ Moderate  ○ High    ○ No included studies |  |  |
| COST EFFECTIVENESS | **Does the cost-effectiveness of the intervention favor the intervention or the comparison?**  ○ Favors the comparison  ● Probably favors the comparison  ○ Does not favor either the intervention or the comparison  ○ Probably favors the intervention  ○ Favors the intervention    ○ Varies  ○ No included studies | No research evidence was identified. |  |
| EQUITY | **What would be the impact on health equity?**  ○ Reduced  ○ Probably reduced  ○ Probably no impact  ○ Probably increased  ○ Increased    ● Varies  ○ Don't know | No research evidence was identified. |  |
| ACCEPTABILITY | **Is the intervention acceptable to key stakeholders?**  ○ No  ○ Probably no  ○ Probably yes  ● Yes    ○ Varies  ○ Don't know | No research evidence was identified. |  |
| FEASIBILITY | **Is the intervention feasible to implement?**  ○ No  ○ Probably no  ○ Probably yes  ● Yes    ○ Varies  ○ Don't know | No research evidence was identified. |  |

Supplement Table S6: Assessment

|  | **JUDGEMENT** | | | | | | | **IMPLICATIONS** |
| --- | --- | --- | --- | --- | --- | --- | --- | --- |
| **PROBLEM** | No | Probably no | Probably yes | **Yes** |  | Varies | Don't know |  |
| **DESIRABLE EFFECTS** | Trivial | Small | **Moderate** | Large |  | Varies | Don't know |  |
| **UNDESIRABLE EFFECTS** | Large | **Moderate** | Small | Trivial |  | Varies | Don't know |  |
| **CERTAINTY OF EVIDENCE** | Very low | **Low** | Moderate | High |  |  | No included studies |  |
| **VALUES** | Important uncertainty or variability | **Possibly important uncertainty or variability** | Probably no important uncertainty or variability | No important uncertainty or variability |  |  | No known undesirable outcomes |  |
| **BALANCE OF EFFECTS** | Favors the comparison | Probably favors the comparison | **Does not favor either the intervention or the comparison** | Probably favors the intervention | Favors the intervention | Varies | Don't know |  |
| **RESOURCES REQUIRED** | Large costs | **Moderate costs** | Negligible costs and savings | Moderate savings | Large savings | Varies | Don't know |  |
| **CERTAINTY OF EVIDENCE OF REQUIRED RESOURCES** | Very low | **Low** | Moderate | High |  |  | No included studies |  |
| **COST EFFECTIVENESS** | Favors the comparison | **Probably favors the comparison** | Does not favor either the intervention or the comparison | Probably favors the intervention | Favors the intervention | Varies | No included studies |  |
| **EQUITY** | Reduced | Probably reduced | Probably no impact | Probably increased | Increased | **Varies** | Don't know |  |
| **ACCEPTABILITY** | No | Probably no | Probably yes | **Yes** |  | Varies | Don't know |  |
| **FEASIBILITY** | No | Probably no | Probably yes | **Yes** |  | Varies | Don't know |  |

Supplement Table S7: Summary of judgement

| Should DEX be used prophylactically? | |
| --- | --- |
| **TYPE OF RECOMMENDATION** | \| Strong recommendation against the intervention \| Conditional recommendation against the intervention \| Conditional recommendation for either the intervention or the comparison \| Conditional recommendation for the intervention \| Strong recommendation for the intervention \| \| --- \| --- \| --- \| --- \| --- \| \| ○ \| ● \| ○ \| ○ \| ○ \| |
| **RECOMMENDATION** | **IN PATIENTS UNDERGOING SURGERY, WE DO NOT SUGGEST THE USE OF ANY DRUG AS A PROPHYLACTIC MEASURE TO REDUCE THE INCIDENCE OF POD (GRADE 2C)** |
| **JUSTIFICATION** | **SERIOUS ADVERSE EFFECTS OF A DRUG THAT HAS BEEN TESTED IN SELECTED POPULATION AND RESULTED IN HIGH HETEROGEINITY OF THE TREAMTENT EFFET FOR PROPHYLACTIC USE IN WIDE POPULATION OF SURGICAL PATIENTS** |
| **SUBGROUP CONSIDERATIONS** | **THE AVAILABLE EVIDENCE SUGGESTS THAT INTRA- OR POSTOPERATIVE USE OF DEXMEDETOMIDINE AS COMPARED TO PLACEBO OR OTHER SEDATIVE DRUGS MAY REDUCE POSTOPERATIVE DELIRIUM FOR PATIENTS UNDERGOING NON-CARDIAC SURGERY** |
| **IMPLEMENTATION CONSIDERATIONS** | **WHEN DEXMEDETOMIDINE IS USED INTRA- OR POSTOPERATIVELY WITH THE AIM TO PREVENT POST-OPERATIVE DELIRIUM, THE EXPECTED BENEFITS SHOULD BE BALANCED AGAINST THE MOST IMPORTANT SIDE EFFECTS (BRADYCARDIA AND HYPOTENSION)** |
| **MONITORING AND EVALUATION** | **WHEN DEXMEDETOMIDINE IS USED INTRA- OR POSTOPERATIVELY WITH THE AIM TO PREVENT POST-OPERATIVE DELIRIUM, THE EXPECTED BENEFITS SHOULD BE BALANCED AGAINST THE MOST IMPORTANT SIDE EFFECTS (BRADYCARDIA AND HYPOTENSION)** |
| **RESEARCH PRIORITIES** | **IDENTIFY THE GROUP AT HIGH RISK OF DELIRIUM**  **TEST WHETHER THE TREATMENT EFFECT OF DEXMEDETOMIDINE IS CONSTANT AND STONG IN THIS RISK GROUP** |

Supplement Table S8: Conclusions

| **Biomarker** | **Number of studies** | | | |
| --- | --- | --- | --- | --- |
|  | **Studies – PubMed Reference** | **Association with POD yes/no** | | |
|  |  | **Pre-operative** | **Intra-operative** | **Post-operative** |
| **Oxidative stress markers** |  |  |  |  |
| Serum 8-iso-Prostaglandin F2α (8-iso-PGF2α) | <https://pubmed.ncbi.nlm.nih.gov/26874041/> | - | - | + |
| Serum S-methyl cysteine | <https://pubmed.ncbi.nlm.nih.gov/28180239/> | + | - | - |
| Serum Thioredoxin (TRX) | <https://pubmed.ncbi.nlm.nih.gov/28093200/> | - | - | + |
| Serum fatty acids  ω3 and ω6  linolenic acid  Cis-9,12-Octadecadienoic acid  cysteine  fumaric  branched-chain amino acid (BCAA)/aromatic amino acid ratio  further acids | <https://pubmed.ncbi.nlm.nih.gov/31711096/>  <https://pubmed.ncbi.nlm.nih.gov/28180239/>  <https://pubmed.ncbi.nlm.nih.gov/28180239/>  <https://pubmed.ncbi.nlm.nih.gov/28180239/>  <https://pubmed.ncbi.nlm.nih.gov/28180239/>  <https://pubmed.ncbi.nlm.nih.gov/31711096/>  <https://pubmed.ncbi.nlm.nih.gov/28180239/> | +  +  +  +  +  +  - |  | +  + |

Supplement Table S9 (continued)

| **Biomarker** | **Number of studies** | | | |
| --- | --- | --- | --- | --- |
|  | **PubMed Reference** | **Association with POD yes/no** | | |
|  |  | **Pre-operative** | **Intra-operative** | **Post-operative** |
| **Markers of nerve cell alteration** |  |  |  |  |
| Serum Apolipoprotein E (ApoE) | <https://pubmed.ncbi.nlm.nih.gov/28383643/>(ApoE4 genotype) | - | na | na |
|  | <https://pubmed.ncbi.nlm.nih.gov/26238230/>(ApoE2/4 genotype) | - | na | na |
| CSF spermidine | <https://pubmed.ncbi.nlm.nih.gov/30862889/> | + | na | na |
| ß-amyloid (Aβ40 / Aβ42) | <https://pubmed.ncbi.nlm.nih.gov/30862889/>(Aβ42) | + | na | na |
|  | <https://pubmed.ncbi.nlm.nih.gov/31730624/> (CSF) | + | na | na |
|  | <https://pubmed.ncbi.nlm.nih.gov/27822051/> (Aß1-40) | - | - (end of surgery) | + (12 h post op) |
| phosphorylated tau (p-tau) and/or total tau (t-tau) | <https://pubmed.ncbi.nlm.nih.gov/33228978/> | + | na | na |
|  | <https://pubmed.ncbi.nlm.nih.gov/31730624/> | - | na | + (change from pre-op to post-op) |
| Brain-derived neurotrophic factor (BNDF) | <https://pubmed.ncbi.nlm.nih.gov/28854532/> | na | + | na |
| b3GNT3 (beta-1,3-N-acetylglucosaminyltransferase) | <https://pubmed.ncbi.nlm.nih.gov/26675981/> | + (CSF before spinal anesthesia) and – (CSF, validation study) | na | na |
| Autoantibody (aAB) biomarker (44 ADMCI panel biomarker, see supplement Table 1 of <https://pubmed.ncbi.nlm.nih.gov/31730624/>) | <https://pubmed.ncbi.nlm.nih.gov/31730624/> | + (CSF before spinal anesthesia) | na | na |

| **Biomarker** | **Number of studies** | | | |
| --- | --- | --- | --- | --- |
|  | **PubMed Reference** | **Association with POD yes/no** | | |
|  |  | **Pre-operative** | **Intra-operative** | **Post-operative** |
| **Markers of neurogenesis and synaptic plasticity** |  |  |  |  |
| Serum and/or CSF cholinesterase (AChE, BChE) | <https://pubmed.ncbi.nlm.nih.gov/32988381/> | + (serum + CSF) | na | + (24 h post-op) |
|  | <https://pubmed.ncbi.nlm.nih.gov/30431498/> | + (serum) | na | + (24 /48 h post-op) |
|  | <https://pubmed.ncbi.nlm.nih.gov/33225437/> | - (serum) | na | + (on post-operative day 0) and a perioperative drop of CHE activity of more than 50% |
|  | <https://pubmed.ncbi.nlm.nih.gov/28560042/> | na | na | - (24 /48 / 72 h post-op) |
| Choline acetyltransferase (ChAT) | <https://pubmed.ncbi.nlm.nih.gov/32988381/> | + (serum + CSF) | na | + (24 h post-op) |
| Monoamine metabolites:  Thyrosine  Tryptophan  Phenyalanine  Methionine | <https://pubmed.ncbi.nlm.nih.gov/27484129/> (CSF) | -  +  +  + | na  na  na  na | na  na  na  na |

| **Biomarker** | **Number of studies** | | | |
| --- | --- | --- | --- | --- |
|  | **PubMed Reference** | **Association with POD yes/no** | | |
|  |  | **Pre-operative** | **Intra-operative** | **Post-operative** |
| **Markers of axonal damage** |  |  |  |  |
| Neurofilament subunit protein H (NFL) | <https://pubmed.ncbi.nlm.nih.gov/30522125/> (serum) | + | na | + (post-op, not further specified plus pre-post-change) |
|  | <https://pubmed.ncbi.nlm.nih.gov/30522125/> (CSF) | (+) with p=0.074 | na | na |
|  | <https://pubmed.ncbi.nlm.nih.gov/31802104/> | - | na | + (change from baseline to post-op day 1) |
|  | <https://pubmed.ncbi.nlm.nih.gov/28504118/> | na | na | + (POD severity) |
|  | https://pubmed.ncbi.nlm.nih.gov/31863758/ | na | na | -(change from end-of-surgery to post-op day 7) |
| Neuroserpin | <https://pubmed.ncbi.nlm.nih.gov/31863758/> | na | + | + (change from end-of-surgery to post-op day 7) |
| Visinin-like protein | <https://pubmed.ncbi.nlm.nih.gov/31863758/> | na | - | -(change from end-of-surgery to post-op day 7) |

Supplement Table S9 (continued)

| **Biomarker** | **Number of studies** | | | | |
| --- | --- | --- | --- | --- | --- |
|  | **PubMed Reference** | **Association with POD yes/no** | | | |
|  |  | **Pre-operative** | | **Intra-operative** | **Post-operative** |
| **Markers of neuroglia injury (blood-brain barrier disruption)** |  |  | |  |  |
| S100 B protein | <https://pubmed.ncbi.nlm.nih.gov/26943607/> | - | | - | + (post-op day 1) |
| Glial fibrillary acidic protein (GFAP) | <https://pubmed.ncbi.nlm.nih.gov/33228978/> | - | | na | - (change from pre-op to post-op) |
|  | <https://pubmed.ncbi.nlm.nih.gov/31863758/> | na | | - | - (change from end-of-surgery to post-op day 7) |
|  |  |  |  | |  |
| **Inflammation markers** |  |  |  | |  |
| Serum CRP | <https://pubmed.ncbi.nlm.nih.gov/31058343/> | - | na | | + (post-op 2-5 days) |
|  | <https://pubmed.ncbi.nlm.nih.gov/29181397/> | + | na | | - (post-op days 1-3) |
|  | <https://pubmed.ncbi.nlm.nih.gov/28555781/> | + | na | | + (post-op day 2) |
|  | <https://pubmed.ncbi.nlm.nih.gov/27822051/> | - | - (end of surgery) | | + (12h after surgery) |
|  | <https://pubmed.ncbi.nlm.nih.gov/29529166/> | + |  | | + (POD 2) |
|  | <https://pubmed.ncbi.nlm.nih.gov/31684066/> | + | na | | - (POD 1 and 3)  + (POD 5) |
| Serum CRP/albumin ratio | <https://pubmed.ncbi.nlm.nih.gov/31326244/> | + | na | | na |
| Cytokines (IL-x) | <https://pubmed.ncbi.nlm.nih.gov/27822051/>  (IL-6) | - | + (end of surgery) | | + (12 h post-op) |
|  | <https://pubmed.ncbi.nlm.nih.gov/31676521/>  (IL-6) | - | na | | + (6-18 h post-op)  + (change from baseline and IL-6 level >= 583 pg/mL at 18h post-op) |
|  | <https://pubmed.ncbi.nlm.nih.gov/31802104/>  (IL-8, out of a panel of 10 cytokines) |  | na | | + (with delirium severity) |
|  | <https://pubmed.ncbi.nlm.nih.gov/26215633/> (pooled cohort = discovery + replication cohort) | + (Il-2) | na | | + (PACU: IL-2 / IL-6) + (post-op day 2: IL-2 / IL-6 / VEGF^$^)+ (post-op day 30: IL-2 / reduced IL-12) |
|  | <https://pubmed.ncbi.nlm.nih.gov/32988381/>  (IL-6, plasma) | + | na | | + (24 h post-op) |
|  | <https://pubmed.ncbi.nlm.nih.gov/32988381/>  (IL-6, CSF) | + | na | | + (24 h post-op) |
|  | <https://pubmed.ncbi.nlm.nih.gov/29529166/>  (Il-2 + IL-6 + CrP) | - | na | | + (post-op day 2) |
| TNF-α | <https://pubmed.ncbi.nlm.nih.gov/26215633/> (pooled cohort = discovery + replication cohort) | - | na | | + (post-op day 30) |
|  | <https://pubmed.ncbi.nlm.nih.gov/32988381/> | + (serum + CNF) | na | | + (24 h post-op) |
| Zinc-alpha-2-glycoprotein (AZGP1) | <https://pubmed.ncbi.nlm.nih.gov/29529166/> | + (together with CrP) | na | | + (post-op day 2) |
| alpha-1 antichymotrypsin (SERPINA3) protein | <https://pubmed.ncbi.nlm.nih.gov/29529166/> | - | na | | - (post-op day 2) |
| White blood cell count, platelet count | <https://pubmed.ncbi.nlm.nih.gov/31684066/>  (total WBC) | + | na | | - (post-op day 1, 3)  + (post-op day 5) |
|  | <https://pubmed.ncbi.nlm.nih.gov/31684066/>  (lymphocyte count) | - | na | | - (post-op day 1)  + (post-op day 3, 5) |
|  | <https://pubmed.ncbi.nlm.nih.gov/31684066/>  (neutrophil count) | - | na | | + (𝚫 preop-nadir_[post-op day 2]_) |
| Neutrophil-to-Lymphocyte Ratio | https://pubmed.ncbi.nlm.nih.gov/33028273/ | + | na | | na |
| Procalcitonin | <https://pubmed.ncbi.nlm.nih.gov/27822051/> | - | + (end of surgery) | | + (12 h post-op) |
|  | <https://pubmed.ncbi.nlm.nih.gov/33256084/> | - | + (before ICU admiss.) | | + (post-op day 1-3) |
| Cortisol | <https://pubmed.ncbi.nlm.nih.gov/27822051/> | - | + (end of surgery) | | + (12 h after op) |
| Monocyte chemoattractant protein 1 (MCP1), also known as chemokine (C-C motif) ligand 2 (CCL2) | <https://pubmed.ncbi.nlm.nih.gov/25943983/> | - | na | | + change in serum concentration from pre-operative to postoperative stage |
| Translocator protein (TP) serum | <https://pubmed.ncbi.nlm.nih.gov/32529881/> | na | na | | + |

| **Biomarker** | **Number of studies** | | | |
| --- | --- | --- | --- | --- |
|  | **PubMed Reference** | **Association with POD yes/no** | | |
|  |  | **Pre-operative** | **Intra-operative** | **Post-operative** |
| **Systemic non-inflammation markers** |  |  |  |  |
| Metabolism markers (albumin, essential fatty acids, insulin-like growth factor-1, IGF-1) | <https://pubmed.ncbi.nlm.nih.gov/29055835/> (albumin) | + | na | na |
| Electrolytes | <https://pubmed.ncbi.nlm.nih.gov/30149051/> (CSF) | + (sodium) | na | na  (potassium, chlorine on post-op day 1) |
| Serum hemoglobin | <https://pubmed.ncbi.nlm.nih.gov/31537309/> | + | na | + (post-op day 1) |
| Melatonin (circadian rhythm) | <https://pubmed.ncbi.nlm.nih.gov/27936113/> (CSF) | - | na | na |

Supplement Table S9 (continued)

| **Biomarker** | **Number of studies** | | | |
| --- | --- | --- | --- | --- |
|  | **PubMed Reference** | **Association with POD yes/no** | | |
|  |  | **Pre-operative** | **Intra-operative** | **Post-operative** |
| **Genetic markers** |  |  |  |  |
| Genome-wide association study (GWAS) | <https://pubmed.ncbi.nlm.nih.gov/30678657/> | 2 SNPs on chromosomes 2 (rs13008718) and on chromosome 14 (rs188623516) as risk loci for POD |  |  |

na = not assessed; $ = vascular endothelial growth factor; WBC = white blood cell count.

Supplement Table S9 Biomarkers of postoperative delirium

**Supplement Chapter 4: Preventive Measures II: non-pharmacological interventions**

Authors: Gabriella Bettelli, Paola Aceto, Riccardo Audisio, Antonio Cherubini, Bruno Neuner, Maria Schubert, Fatima Yürek

Detailed flow-chart and screening process:

The titles and abstracts of 250 potentially relevant studies were screened independently by two members of the group to identify RCTs and systematic reviews and meta-analyses, see Supplement Figure S6 below. These 250 studies were 124 plus 55 studies assigned to the Preventative measures-2 working group plus 71 out of (205 + 57) studies assigned to multiple working groups, see Supplement Figure S1. Of the 250 studies, 185 (74%) were excluded because they were no RCTs or systematic reviews or did not deal with POD). Of the 65 remaining references, 12 were systematic reviews ± meta-analyses, 40 were RCTs and 13 were studies using a pre-post design. The 12 systematic reviews ± meta-analyses summarized a total of 86 RCTs. Thus, all together 126 (40+86) potentially relevant single RCTs were screened for the above-mentioned POD inclusion criteria. As shown in Supplement Figure S6, from the remaining 126 studies 107 (84.9%) were excluded because of duplicate publication, basic exclusion criteria or they did not meet the POD definition.

The remaining 19 (15.1%) relevant studies were extracted using the following criteria (see Supplement Tables S10-S12):

Number of study participants, age distribution, type of surgery, definition of inclusion and exclusion criteria, description of the intervention, description of the control condition, absolute and relative number of outcomes (number of patients with POD in each study group).

Supplement Figure S6: Flow chart of the Preventive measures II (PM-II) working group literature search procedure


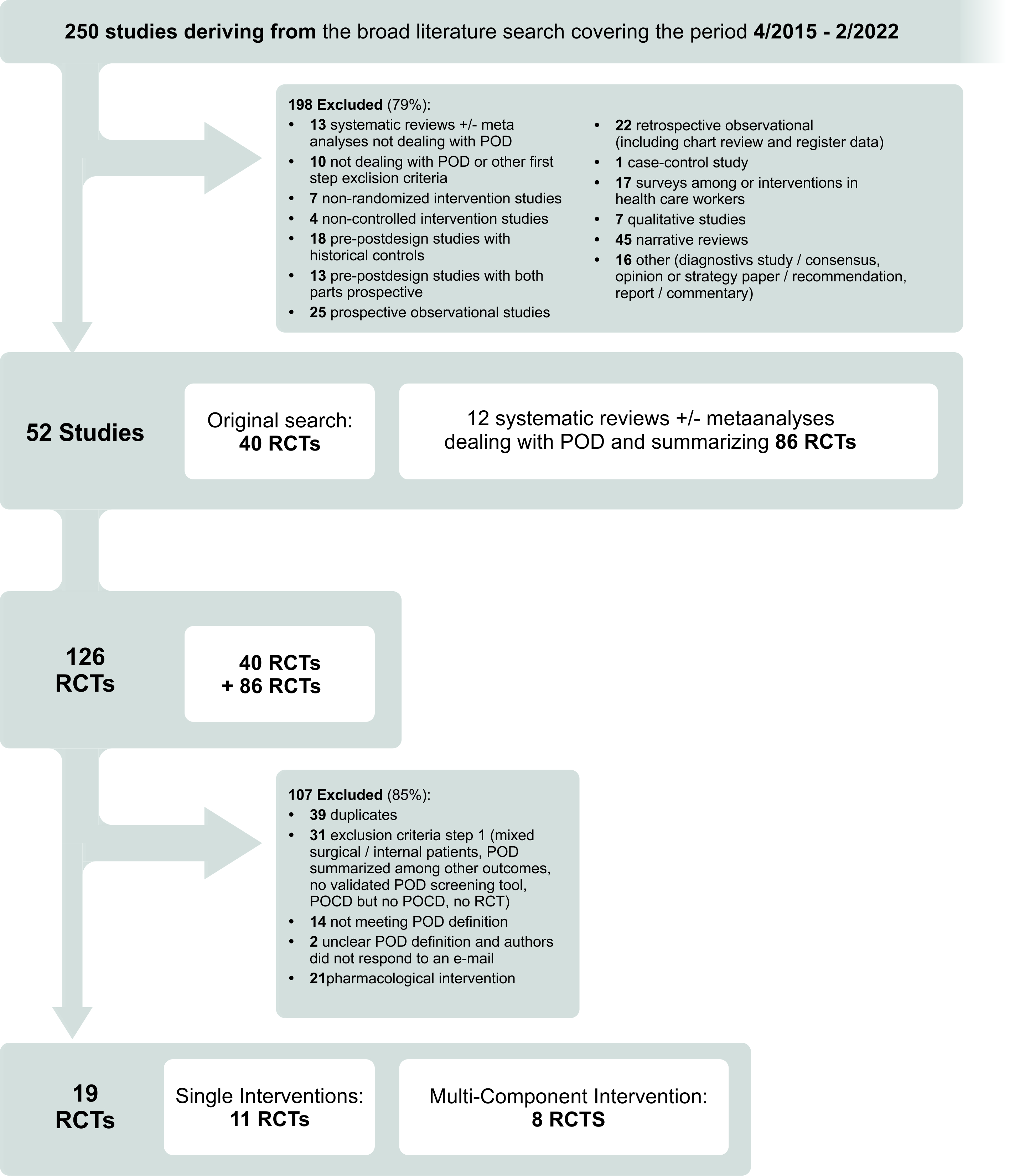


All eight RCTs on multi-component interventions included patients aged >65 years with two studies including patients > 70 years^2,3^. The unweighted mean age of all patients in the eight RCTs was 76.5 years (see Supplement Table S10). Two studies evaluated overall 1141 cardiac patients: one study included only cardiac patients^4^, the second study with patients in multiple surgical disciplines presented stratified results for the subgroup of cardiac patients^2^. Two studies^5,6^ with overall n=445 patients were on hip fracture patients only. The other studies were on different single (oral cancer surgery^7^, aortic repair or lower-limb arterial surgery^8^) or mixed surgical patients groups^3,9^. With regard to inclusion and exclusion criteria, 7/8 (88%) studies^2-8^ included consecutive patients while one single study included only patients with frailty^9^. Four studies^2,3,5,6^ (50%) excluded explicitly terminally ill patients, three studies^3,4,7^ (38%) excluded patients with known psychiatric disorders (including alcohol use disorders) and 3/5 (38%) of the studies excluded patients with a MMSE examination below a predefined threshold or known dementia^3,4,7^.

| Author | Year | n | Type of surgery | Age inclusion criteria | Observed mean age |
| --- | --- | --- | --- | --- | --- |
| Deeken | 2022 | 1470 | elective orthopedic, general, or cardiac surgery | ≥ 70 yrs | ~ 78 |
| Guo | 2016 | 160 | oral cancer surgery | ≥ 65 yrs | 73.5 |
| Hempenius | 2013 | 260 | elective surgery for a solid tumor | ≥ 65 yrs | 77.54 |
| Marcantonio | 2001 | 126 | hip fracture surgery | ≥ 65 yrs | 79 |
| Olotu | 2022 | 609 | cardiovascular surgery | ≥ 60 yrs | 71.8 |
| Partridge | 2017 | 176 | elective aortic aneurysm repair, lower-limb arterial surgery | ≥ 65 yrs | 75.5 |
| Vidán | 2005 | 319 | hip fracture surgery | ≥ 65 yrs | 81.87 |
| Wang | 2020 | 281 | gastric, colorectal, pancreatic, biliary, thoracic, and thyroid surgery | ≥ 70 yrs | 74.7 |

Supplement Table S10: Study parameter of the included RCTs on multicomponent interventions, n = 8 RCTs

| Author | Multicomponent interventions |
| --- | --- |
| Deeken | Intervention "AKTIVER":  7 best-practice delirium prevention modules: cognitive, motor, and sensory stimulation; meal companionship; diagnostic test and operating room accompaniment; stress relaxation; and sleep promotion. [program described in detail in Table 1 of the original publication]  “The independent delirium study prevention team observed patients throughout hospitalization and provided the intervention modules several times a day as needed” |
| Guo | setting with educated staff / pre-op visits to the ICU / medical equipment introduced / reorientation strategies / sleep hygiene / eyeshade and earplugs / catheter removal / music / early nasal feeding) |
| Hempenius | multicomponent intervention with pre-op assessment by a geriatric team, which supervised an individual treatment plan, with specific attention to patient-related risk factors for delirium |
| Marcantonio | proactive geriatrics consultation within 24h after surgery: daily visits by a geriatrician with targeted recommendations based on a structured protocol (oxygen delivery, fluid + electrolyte balance, pain treatment, medication optimization, catheter removal, optimization of nutritional intake, early mobilization, complication management, environment stimuli, eventual treatment of delirium) |
| Olotu | delirium prevention bundle including reorientation measures, sleeping aids and early mobilization, early catheter removal, early nutrition commencement, from post-op day 1-7 |
| Partridge | CGA and optimization in an outpatient clinic setting (cognitive impairment: tailored information/discussion and referral to a local memory assessment service. Anemia: Vitamin B12 substitution or iron orally or i.v. Cardiac assessment: coronary intervention where indicated. Impaired functional independence: referral to physiotherapist and occupational therapist. Nutrition: optimization strategies when necessary. Mood disorder: GP or psychiatric service when necessary. Plus team-based optimization of the antiplatelet management for complex aneurysm repair |
| Vidán | Geriatric evaluation (medical and psychosocial problems, functional capability to elaborate a therapeutic plan. Daily visits). Rehabilitation specialist (physical therapy). Social worker (social environment network, improve the social support when necessary). |
| Wang | tailored HELP protocol daily from postoperative day 1 to postoperative day 7 or discharge with 1) assessment of risk factors within 24h after admission, 2) three universal protocols targeting orientation, therapeutic activities, and early mobilization, 3) eight targeted protocols (should be in supplement 2, not accessible) |

Supplement Table S11: Type of multi-component intervention in the intervention group, n = 8 RCTs

| **PICO** | | **P patients undergoing surgery**  **I multi-component or single non-pharmacological intervention/s**  **C usual care**  **O postoperative delirium (according to the ‚new‘ criteria)** | | | | | | | | | | |
| --- | --- | --- | --- | --- | --- | --- | --- | --- | --- | --- | --- | --- |
| **Study ID** | | **Risk of bias assessment (RoB 2)** | | | | | | **No of outcomes** | | **Effects** | | **Quality**  ⨁⨁  ◯◯ |
| **No** | **Author** | **Rando-misation** | **Intended intervention** | **Missings** | **Outcome** | **Selection of results** | **Overall bias** | **Intervention** | **Control** | **Relative (95% CI)** | **Absolute (95% CI)** |  |
| 1 | Deeken 2022 | low | low | low | low | some concerns | low | 147/740 (19.9%) | 171/730 (23.4%) | **RR 0.85**  (0.70 to 1.03) |  |  |
| 2 | Guo 2016 | low | low | high^1^ | low | some concerns | high | 10/81 (12.3%) | 25/79 (31.6%) | **RR 0.39**  (0.20 to 0.76) |  |  |
| 3 | Hempenius 2013 | low | low | high^2^ | low | low | high | 12/127 (9.4%) | 19/133 (14.3%) | **RR 0.66**  (0.33 to 1.31) |  |  |
| 4 | Marcantonio 2001 | low | low | low | low | low | low | 20/62 (32.3%) | 32/64 (50.0%) | **RR 0.65**  (0.42 to 1.00) |  |  |
| 5 | Olotu 2022 | low | low | high^3^ | some concerns | low | high | 37/284 (13.0%) | 47/274 (17.2%) | **RR 0.76**  (0.51 to 1.13) |  |  |
| 6 | Partridge 2017 | some concerns | low | high^4^ | low | some concerns | high | 9/85 (10.6%) | 22/91 (24.2%) | **RR 0.44**  (0.21 to 0.90) |  |  |
| 7 | Vidán 2005 | low | low | low | some concerns | some concerns | some concerns | 53/155 (34.2%) | 67/164 (40.9%) | **RR 0.84**  (0.63 to 1.11) |  |  |
| 8 | Wang 2020 | low | some concerns | low | low | low | low | 4/152 (2.6%) | 25/129 (19.4%) | **RR 0.14**   - 1. to 0.38) |  |  |

1: High risk of bias due to concerns regarding missing outcome data. Of 182 patients randomized, 22 (12.1%) were not analyzed. The observed number of events (25+10 = 35) was not much greater than the number of participants with missing outcome data.

2: High risk of bias due to concerns regarding the number of participants who were allocated to the study arms and finally analyzed: n=148 allocated to intervention and n=127 (86%) analyzed, n= 149 allocated to control and n=133 (89%) analysed.

3: High risk of bias due to concerns regarding missing outcome data. Of 609 patients randomized, 51 (8.4%) dropped out before allocation. The observed number of events (37+47 = 84) was not much greater than the number of participants with missing outcome data.

4: High risk of bias due to concerns regarding missing outcome data. In the intervention group (n=105), no primary outcome data were evaluated in13 (12.4%) of patients and in the control group 18 out of 104 (17.3%) were missing. There were no concerns regarding treatment allocation (in both groups < 5%).

Supplement Table S12: Risk-of-bias assessment following the GRADE algorithm (RoB-2), n=8 RCTs


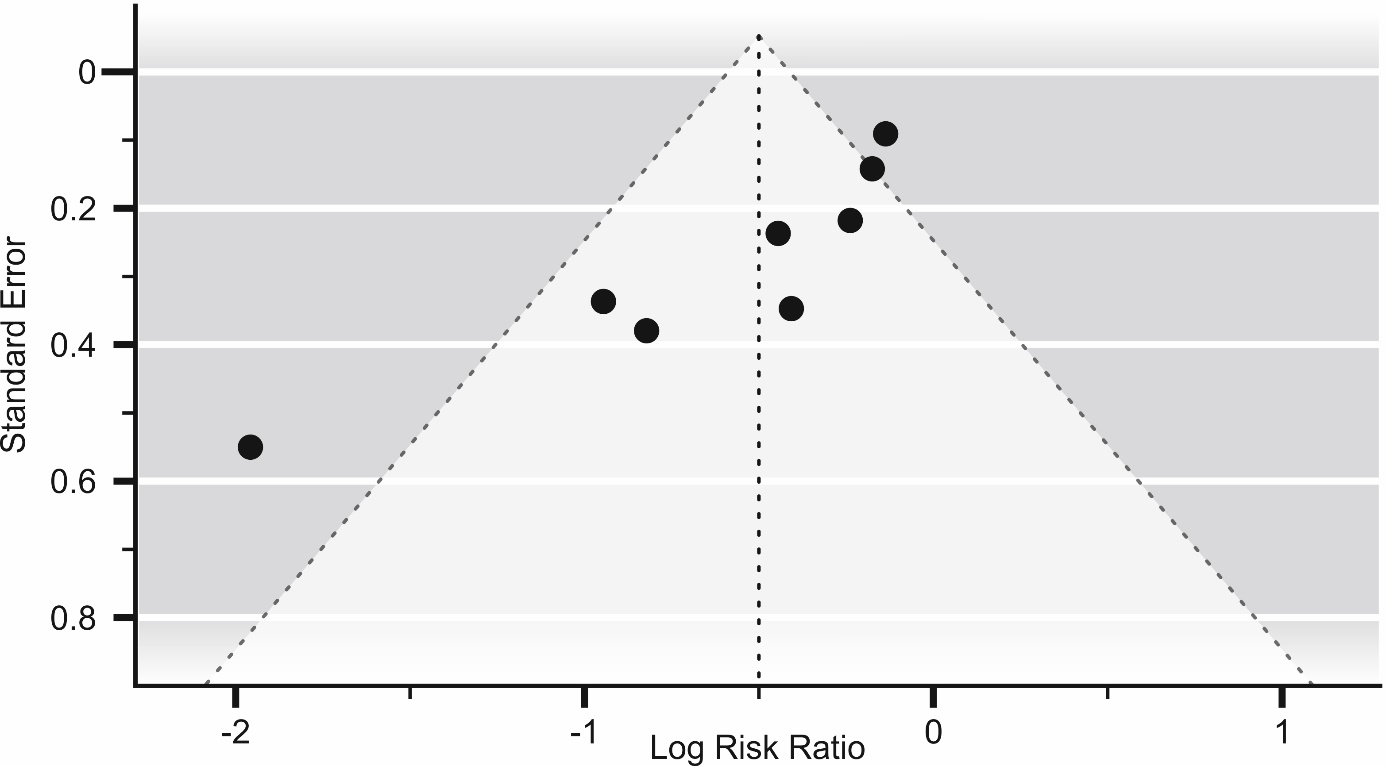


Supplement Figure S7: Funnel plot for POD outcome in multicomponent interventions versus usual care, n=8 studies


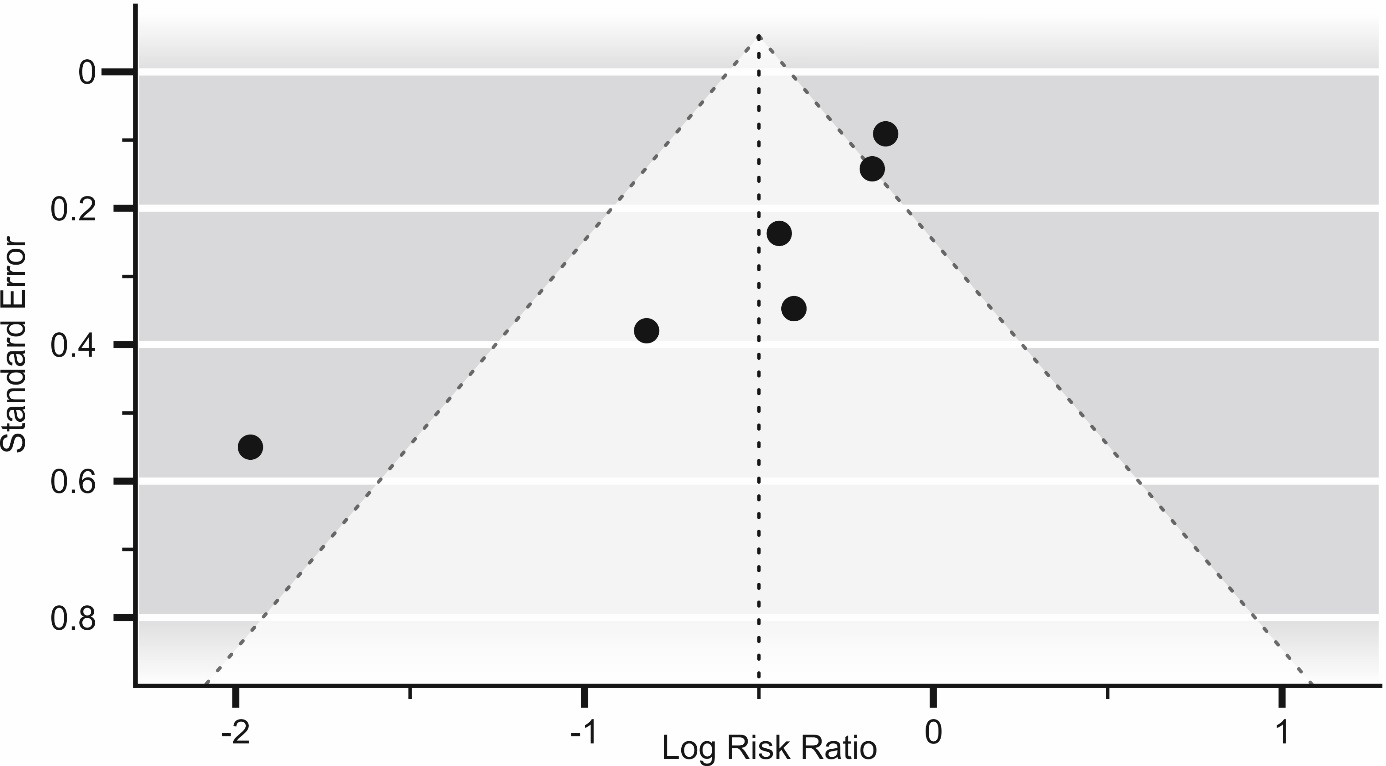


Supplement Figure S8: Funnel plot for POD outcome in multicomponent interventions versus usual care after (comprehensive) geriatric assessment plus tailored interventions, n=6 studies

| Author | Year | n | Type of surgery | Age inclusion criteria | Observed mean age |
| --- | --- | --- | --- | --- | --- |
| Fahimi | 2019 | 110 | CABG | ?? | 57 |
| Fazlollah | 2021 | 60 | CABG | 35-70 yrs | 64 |
| Giraud | 2016 | 223 | elective or urgent cardiac surgery | ≥ 70 yrs | 77.2 |
| HIP Attach invesigators | 2020 | 2970 | hip fracture surgery | ≥ 45 yrs | 79 |
| Jia | 2013 | 240 | Open colorectal surgery in colorectal carcinoma | ≥ 70 yrs | 75 |
| Kanova | 2021 | 52 | Smokers (≥ 10 cigarettes/day) undergoing major surgery (urology, traumatology, orthopaedics or head and neck surgery (including dental surgery) with a subsequent stay in the ICU of >24 hours | ≥ 18 yrs | 59 |
| Kudoh | 2002 | 80 | Orthopedic surgery under general anesthesia with major depression and receiving antidepressants | 35-63 | 50 |
| McCaffrey | 2009 | 22 | Hip or knee surgery | ?? | 75 |
| O’Gara | 2020 | 45 | Cardiac surgery | 60-90 | 70 |
| Vlisides | 2019 | 61 | Major gastrointestinal / urologic / spine or hepatobiliary surgery | ≥ 60 yrs | 67 |
| Xue | 2020 | 133 | Cardiac surgery | ≥ 18 yrs | 58 |

Supplement Table S13: Study parameter of the RCTs on single interventions, n = 11 RCTs

| Author | Single intervention |
| --- | --- |
| Fahimi | Patients of the intervention group were provided with a multimedia CD containing three short educational videos of 4 to 6 minutes 5 to 7 days before the surgery. In the first video, a cardiologist, who fully mastered the CABG procedure, provides information about the disease process and procedures for CABG and the required equipment, such as cardiac monitoring, different catheters, chest tubes, and artificial respiration device. In the second video, a nurse, with at least 5 years of work experience in the heart surgical intensive care unit and full mastery of respiratory exercises and other relevant issues, describes postoperative measures and special care provided in the department of cardiac surgery, patient visitation schedule and procedure, respiratory exercises, exercise for the foot that undergoes surgery and possible complications, and bed leave time. In the third video, a person who has already undergone CABG shares his or her pre- and postoperative experiences with the patient. |
| Fazlollah | One hour after the endotracheal tube was removed, the intervention was done. Prior to starting the foot reflexology massage, the hands were lubricated with baby oil, which has no therapeutic effect. In the first step, the foot was taken one minute in the hand until the oil was distributed to the entire foot. Then, relaxation techniques were performed for two minutes for each foot. The brain’s reflection points, pituitary, and hypothalamic in both legs were massaged for 10 min in the third step. The pointing and thumb fingers were used to press on certain parts of the soles of the feet. Finally, two minutes of relaxation techniques were performed on both legs. The overall massage time was twenty minutes. This procedure was repeated once a day for two consecutive days. |
| Giraud | The intervention commenced from the time all anesthetic agents were switched oﬀ and the patient was awake following surgery. It was administered by patient’s nursing and physiotherapy teams and consisted in the use, and coaching in the use, of two types of mirrors to support mental status and attention, physical mobilization, and multisensory feedback and integration. The mirrors included: (i) a standard 23 × 41 cm unbreakable personal mirror of the type used in clinical/therapeutic settings where viewing of the face is desired (e.g., speech therapy) and (ii) a standard 160 × 50 cm mobile posture mirror of the type used in physical/occupational therapy to provide visual feedback supporting proprioception (e.g., rehabilitation following stroke). |
| HIP Attach investigators | Patients randomised to accelerated surgery underwent medical clearance by physicians who were available to rapidly evaluate these patients. After obtaining medical clearance, these patients moved into the next orthopaedic elective or trauma operating room slot (i.e., they were prioritised over elective cases and other non-emergent trauma cases). /…/ All patients in the accelerated-surgery and standard-care groups underwent medical assessment and clearance before surgery. The difference between the groups was that a physician was available to undertake rapid medical assessment of patients in the accelerated-surgery group, /…/ |
| Jia | Preoperative preparation: oral purgatives, no mechanical enema, normal meal until 6 h before surgery, normal carbohydrate drink until 2h before surgery, no nasogastric tube insertion, no antibiotics  Anaesthesia: Thoracic epidural  Pain control: Ropivacaine 2mg / ml, via PCEA, for 48 h, opium-derived agents were excluded, no routine drainage tube placement  Postoperative management: Diet: water was allowed from 6 h post-operation, liquid diet in the morning and semiliquid diet at noon and evening of the first and second postoperative days, regular diet on POD 3, urinary catheter withdrawal on POD 1–2, out-of-bed mobilization on POD 1 |
| Kanova | 21 mg transdermal nicotine patch within 24h after ICU admission, daily changes, up to 7 days or discharge |
| Kudoh | Continuing of antidepressants (except for the day of surgery) |
| McCaffrey | Upon arrival at the orthopaedic floor from the recovery area, experimental participants listened to a CD of soothing lullaby music, which played continuously on the CD player. Once the participant was awake and alert, he or she could choose from a variety of music provided by the researchers. /…/ During the recovery period, the CD player was set to play at least four times a day for 1 hour. The patient could play the music more often, if desired, by pressing the play button or by asking hospital staff to do so when they entered the room. |
| O’Gara | Cognitive training (CT) consisted of a mobile software application /…/ featuring programs designed to train users in the cognitive domains of memory, attention, problem solving, flexibility, and processing speed. Each program automatically adjusts the difficulty of the subsequent level to maintain a balance between cognitive challenge and enjoyment. Participants in the CT group were instructed to train for 2 separate 15-minute sessions per day, from the day of enrollment until 4 weeks after surgery including the immediate postoperative period. During each session, participants were asked to select ≥1 game from each of the 5 available cognitive domains. |
| Vlisides | This study utilized an adaptive, computer-based cognitive training battery that specifically targets executive function, attention, working memory, and visuospatial processing /…/, which are cognitive domains particularly affected by delirium.11, 12 The specific games chosen were Divided Attention, Double Decision, Card Shark, Juggle Factor, and Eye for Detail /…/. |
| Xue | Individualized education, according to the patients’ characteristics, was conducted for the experimental group by a group of trained educators. /…/ A set of education leaflets were made for a better understanding of the theme-“what’s going on after the surgery”. Different education ways were selected according to the age, gender, education level, and surgery type. The leaflet contents can be separated into three parts. The first part introduced the operation process with colorful illustrations, the second part demonstrated the sights, sounds, and nursing care to be anticipated postoperatively, and the third part gave an introduction to the equipment used in ICU. The education would be launched about three days before the surgery when the patient and family were available when the educator welcome them at first and then distributed the leaflets. Next, the educator would try to make the education through and eliminate the anxiety of the patients. Actual instruments in ICU would be used as a tool for learning, and also a tour to ICU would be made when applicable. The focus would be put on the familiarization of the postoperative process and the request for the cooperation of the patients. |

Supplement Table S14: Type of single intervention in the intervention group, n = 11 RCTs

**Supplement Chapter 5: Neuromonitoring**

Authors: Susanne Koch, Nicola Latronico, Alasdair MacLullich, Simone Piva, Finn Radtke, Robert Sanders, Concezione Tommasino

Literature Search Procedure

Based on the initial literature search with the primary focus on the effects of EEG monitoring on postoperative delirium (POD) in adult patients (> 18 years), 111 publications were retrieved (see Supplement Figure S1, 46 + 24 publications assigned to the Neuromonitoring working group + 41 out of (205 + 57) publications assigned to multiple working groups). The title and abstract were screened independently by two members of the group (SK, SP) to exclude narrative reviews (n=14), studies not dealing with intraoperative EEG monitoring (n=38), and studies with an inadequate study design (n=33; focusing on emergence delirium, POD diagnostics, retrospective analysis, or EEG data modelling studies). Additionally, 4 studies were included: 3 RCT trials^10-12^ that were published before the research period, and 1 recently published RCT^13^ and were online available in December 2021, but did not show up in the overall literature search (Supplement Figure S9 – flow chart).

The panel members finally evaluated and discussed 30 full-text studies: 12 RCT trials^10-21^, 10 observational, conducting raw EEG data analysis, mainly focusing on burst suppression activity^22-26^ and intraoperative EEG signatures^27-31^, and 8 systematic reviews and meta-analyses^32-39^.


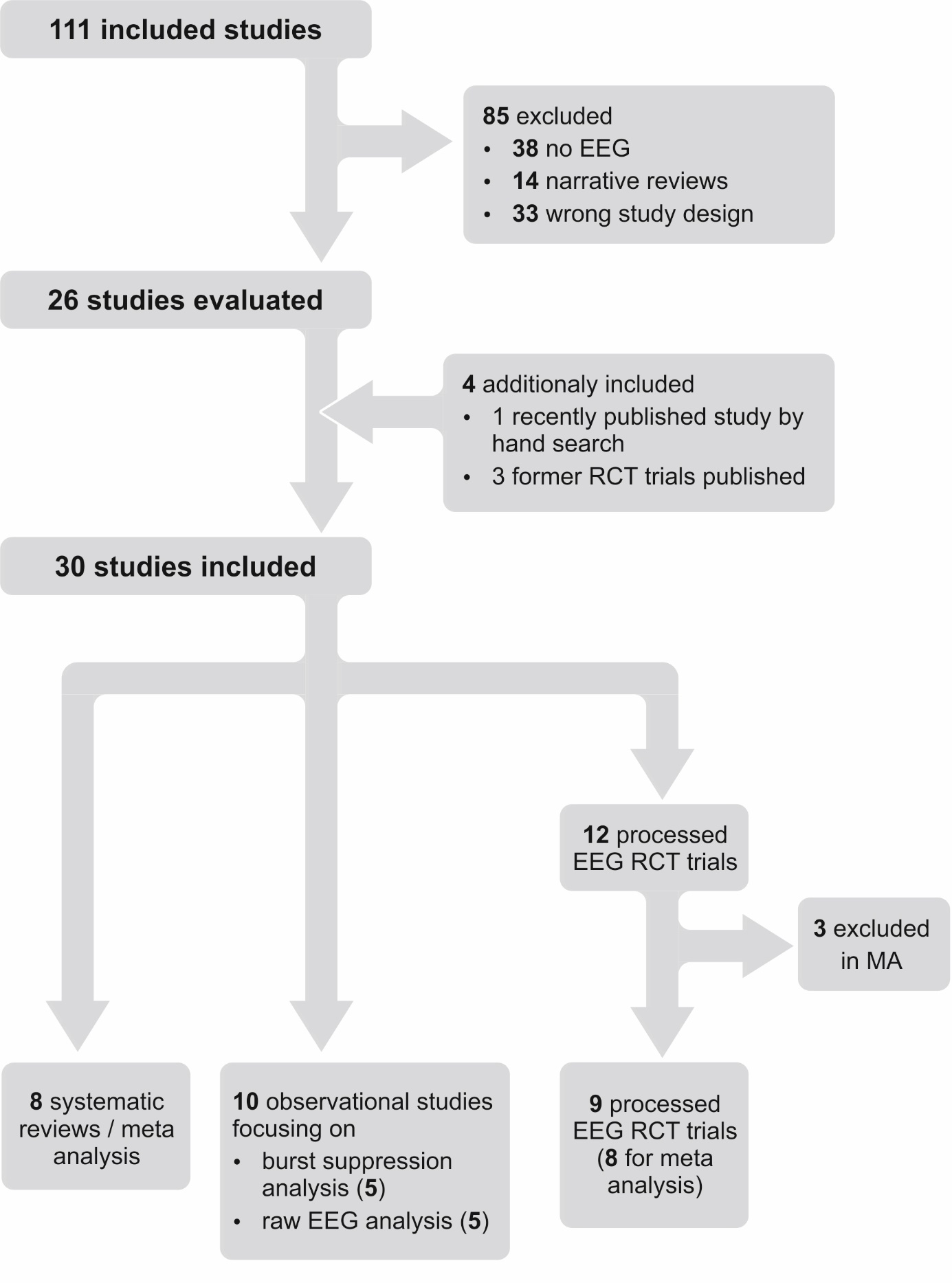


Supplement Figure S9: Flow chart of the literature search procedure in the Neuromonitoring working group.

**
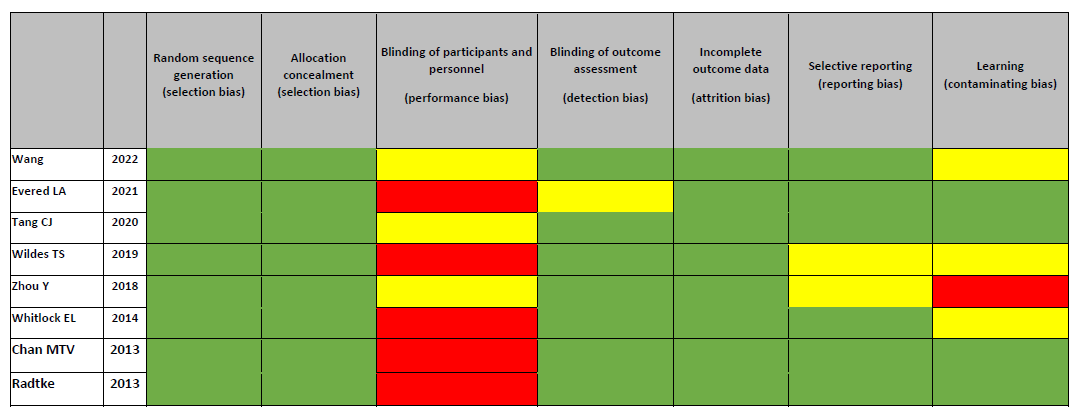
**Supplement Table S15: Risk of bias assessment for the included RCTs in the Meta-analysis

**Supplement Chapter 6: Pharmacological Treatment of POD and POD Outcomes**

Authors: Claudia Spies, Nicola Latronico, Anika Müller, Alasdair MacLullich, Finn Radtke, Lisa Vasiljewa

Literature Search Procedure

The Treatment & Outcome working group conducted their own systematic search (based on the initial literature search) in September 2021 and the update on March 31st 2022, via PubMed using the following MeSH Terms:

(delirium OR confusion OR confusion* OR disorientation OR bewilderment) AND (postoperative OR postoperative period OR postoperative period* OR post surgical OR post-surgical OR anesthesia recovery period OR anesthesia recovery period* OR postanesthesia) AND (treatment OR therapy OR medical treatment)

There were no language restrictions applied to the search.

Randomized-controlled studies and meta-analyses which evaluated post-operative patients >18 years old, who were diagnosed with delirium (see the *definition of postoperative delirium* in the *general approach* paragraph) during hospitalization and underwent any pharmacologic interventions compared to no intervention, placebo, or other pharmacologic treatment were considered. Outcomes of interest were delirium severity, delirium length, hospital- and ICU lengths of stay, and mortality.

After initially getting 4768 results we excluded all the studies published before 01.03.2015 (the date of the last update of the ESAIC POD Guideline). In addition to the inclusion and exclusion criteria from the general methods section, the following studies were also excluded: cohort studies, prospective observational studies, and studies on the prevention of POD.

Ultimately, 22 full-text articles (14 in the primary inclusion, 6 individual studies extracted from the meta-analyses, and 2 studies from the broad search) were included in the final analysis, so that 5 Meta-analysis and 17 RCTs were analysed in evidence tables.

The search and the study selection were performed by two authors independently using the Rayyan tool for systematic reviews in the blind mode. A flow chart of the study selection process is shown here (see Supplement Figure S10):


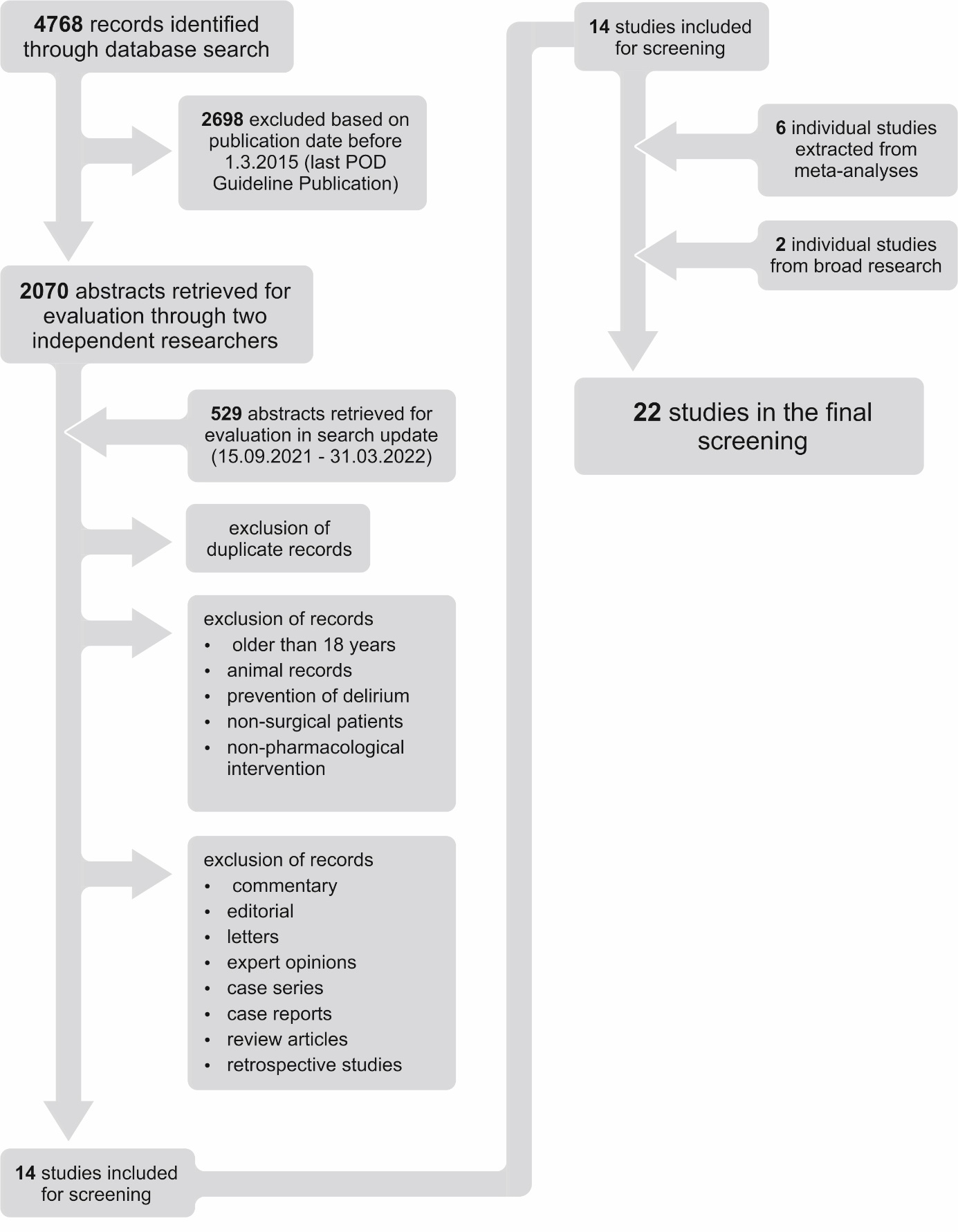


Supplement Figure S10: Flow chart of the literature search procedure in the Treatment & Outcome working group.

| 1 | Yapici 2011 | Alpha 2-Agonists | RCT | First line, initial search |
| --- | --- | --- | --- | --- |
| 2 | Djaiani 2016 | Alpha 2-Agonists | RCT | First line, initial search |
| 3 | Su 2016 | Alpha 2-Agonists | RCT | Second line, from SR |
| 4 | Pieri 2019 | Alpha 2-Agonists | SR+MA | First line, initial search |
| 5 | Shokri 2019 | Alpha 2-Agonists | RCT | First line, initial search |
| 6 | Subramaniam 2019 | Alpha 2-Agonists | RCT | First line, initial search |
| 7 | Likhvantsev 2020 | Alpha 2-Agonists | RCT | First line, initial search |
| 8 | Momeni 2020 | Alpha 2-Agonists | RCT | First line, initial search |
| 9 | Kalisvaart 2005 | Antipsychotics | RCT | Second line, from SR |
| 10 | Hakim 2012 | Antipsychotics | RCT | Second line, from SR |
| 11 | Wang 2012 | Antipsychotics | RCT | Second line, from SR |
| 12 | Fukata 2014 | Antipsychotics | RCT | Second line, from SR |
| 13 | Neufeld 2016 | Antipsychotics | SR+MA | First line, initial search |
| 14 | Fukata 2017 | Antipsychotics | RCT | Second line, from SR |
| 15 | Shen 2018 | Antipsychotics | SR+MA | First line, initial search |
| 16 | Oh 2021 | Melatonin | RCT | First line, initial search |
| 17 | Shi 2021 | Melatonin | RCT | First line, initial search |
| 18 | Zadeh 2021 | Melatonin | RCT | First line, initial search |
| 19 | Gupta 2021 | Anticonvulsants | SR+MA | First line, initial search |
| 20 | Xu 2021 | Rosuvastatin | RCT | First line, initial search |
|  |  |  |  |  |
| from broad search: | |  |  |  |
| 21 | Leigh 2019 | pharmacological treatment of POD in cardiac surgery | SR+MA | found in broad search |
| 22 | Atalan 2013 | Antipsychotics | RCT | Second line, from SR |

Supplement Table S16: Studies on POD Treatment included in the final screening

The working group members decided against performing a meta-analysis of the studies due to the strong heterogeneity of the evidence, especially since study populations, interventions and control conditions differed substantially between studies. Heterogeneity tests showed up to I² >95%.

Randomized controlled trials were graded using the Risk of Bias tool (RoB-2) from the Cochrane Collaboration. Based on the analyzed evidence and its quality four recommendation drafts had been formulated by the authors.

Results from the evidence tables were presented in the consensus conferences to the guideline group regarding benefits and harms. Moreover, the comprehensive evidence to decision criteria from MAGICapp© Values and Preferences, Resources, Equity, Acceptability, and Feasibility were discussed for each recommendation. No systematic literature search was performed to get information on these criteria.

Following chapter 15 of the GRADE guidelines and considering four domains deriving from six determinants, recommendations were either considered ‘strong’ or ‘weak’.

The strength of recommendation was agreed on during the final meeting of the Task Force and the Advisory Board at the ESAIC Euroanaesthesia 2022 congress in Milano, Italy on the 5^th^ of 2022.


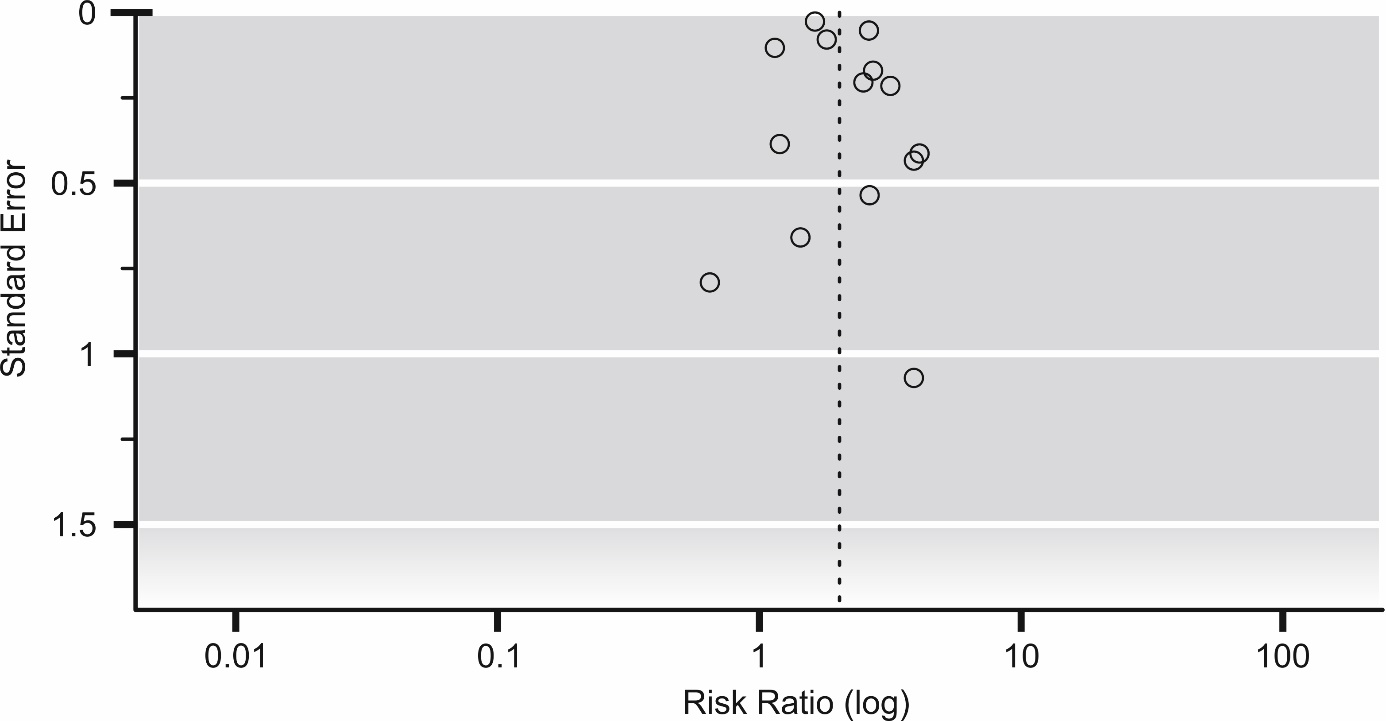


Supplement Figure S11: Funnel plot for mortality in patients with post-operative delirium after non-cardiac surgery vs. patients with no POD


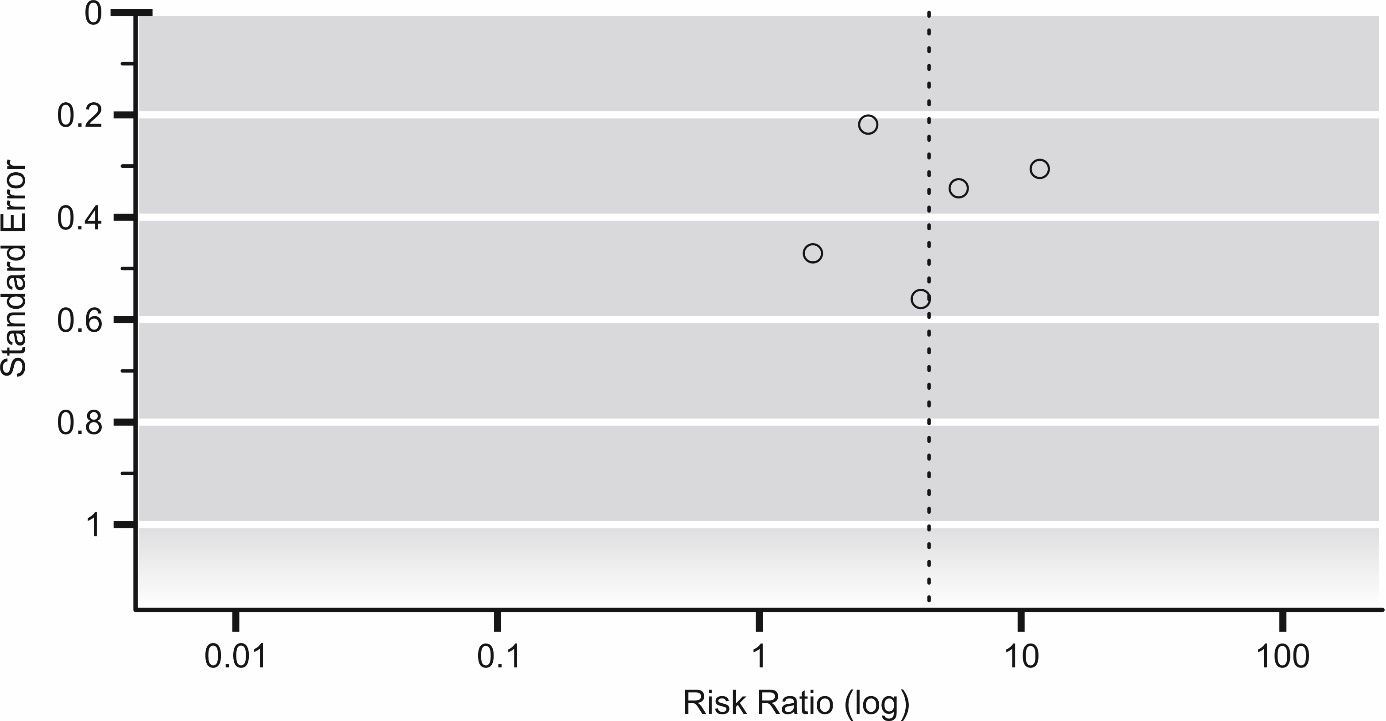


Supplement Figure S12: Funnel plot for mortality in patients with post-operative delirium after cardiac surgery vs. patients with no POD

**
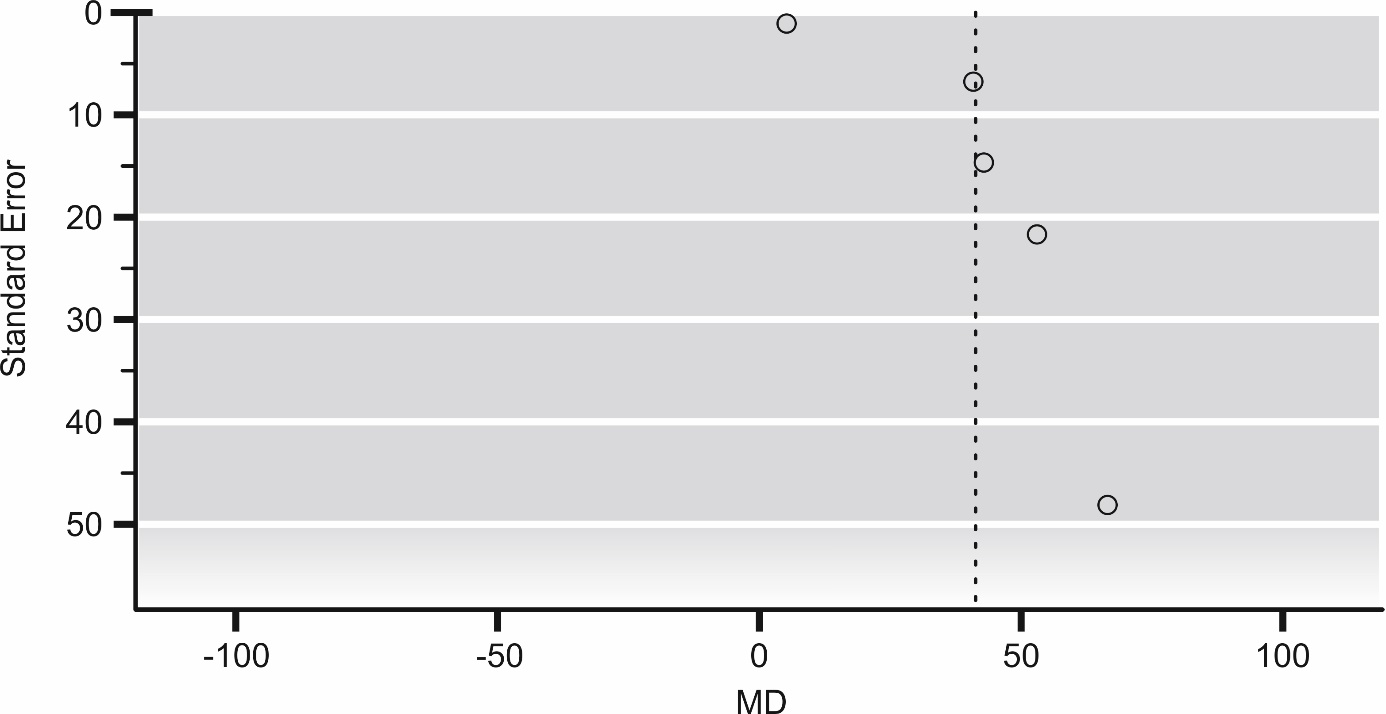
**

Supplement Figure S13: Funnel plot for ICU length of stay in patients with post-operative delirium after cardiac surgery vs. patients with no POD


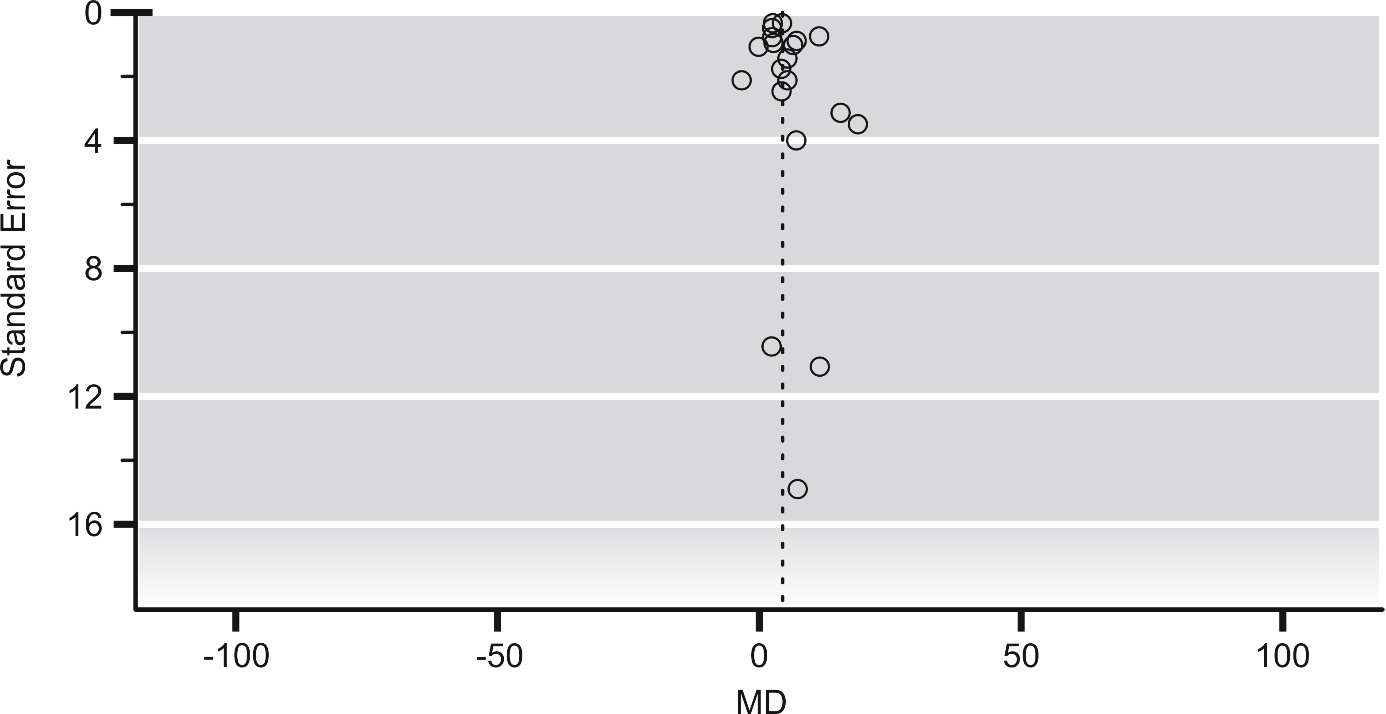
Supplement Figure S14: Funnel plot for hospital length of stay in patients with post-operative delirium after non-cardiac surgery vs. patients with no POD


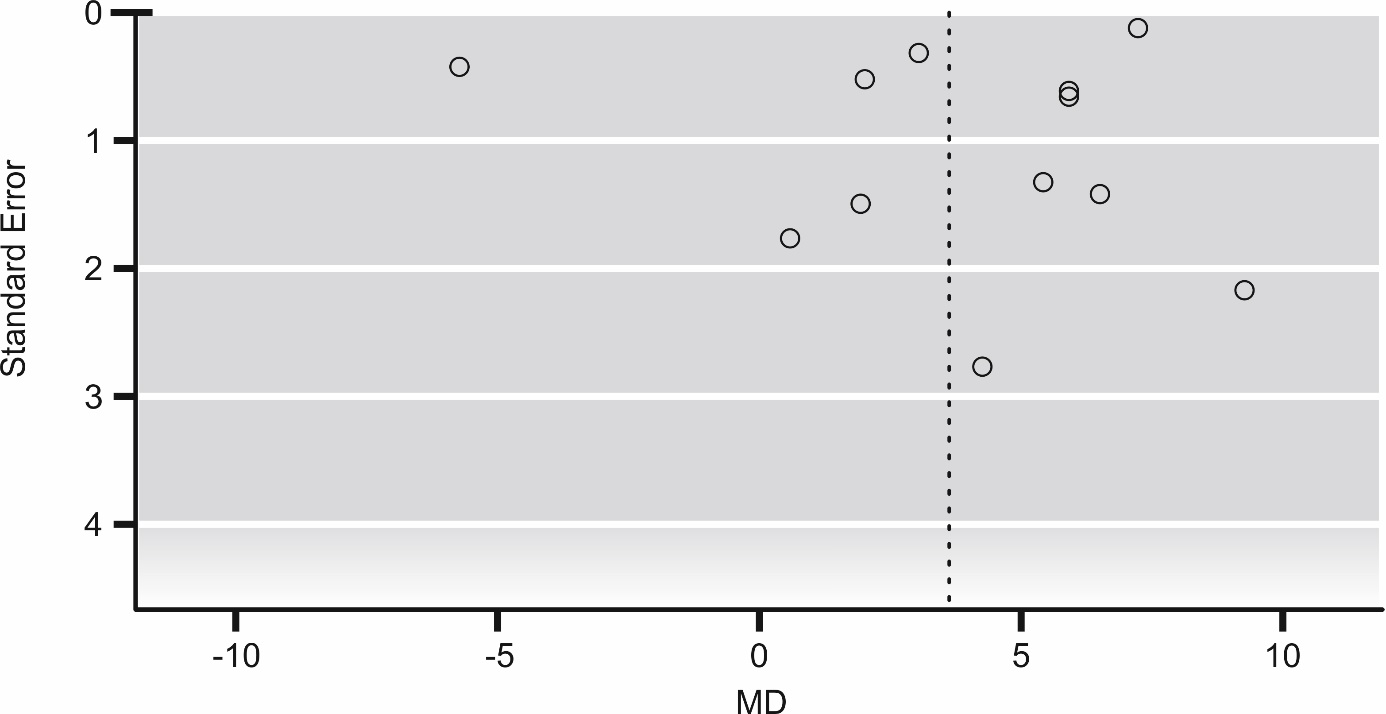


Supplement Figure S15: Funnel plot for hospital length of stay in patients with post-operative delirium after cardiac surgery vs. patients with no POD

 
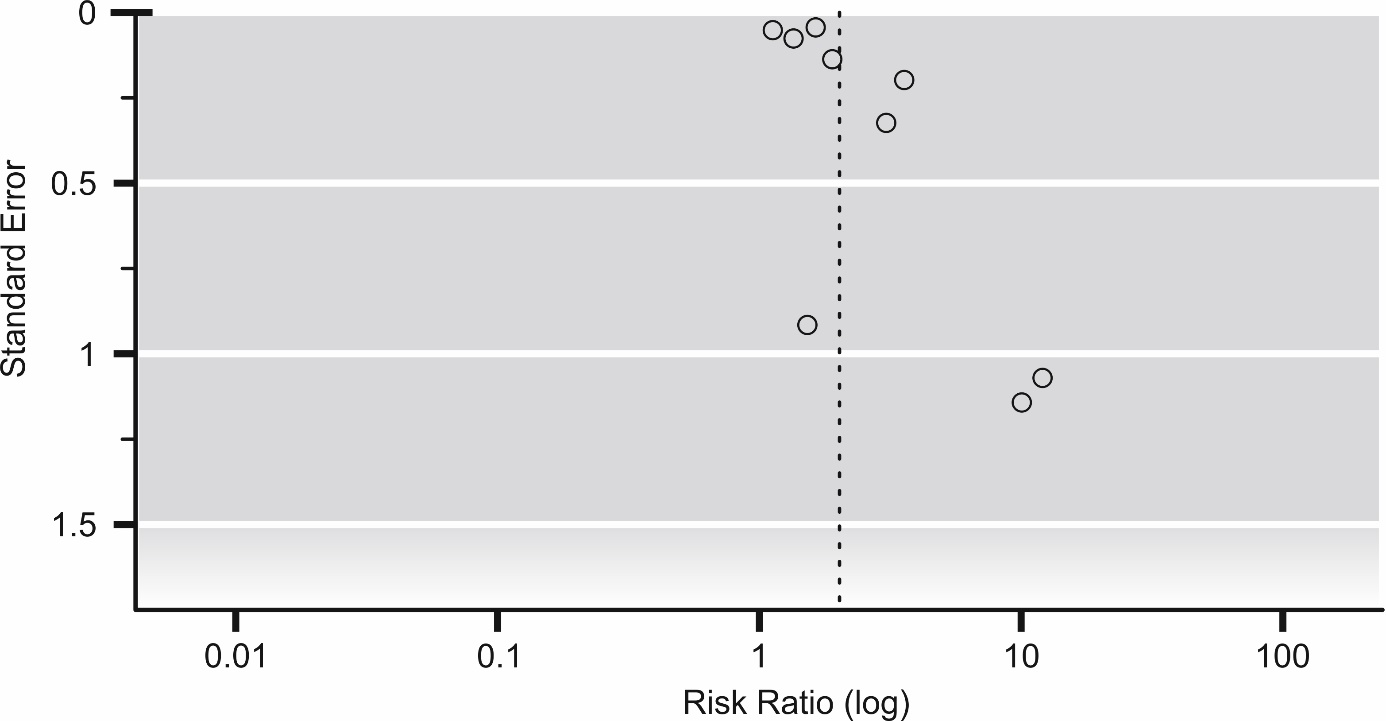


Supplement Figure S16: Funnel plot for need for nursing care in patients with post-operative delirium after non-cardiac surgery vs. patients with no POD


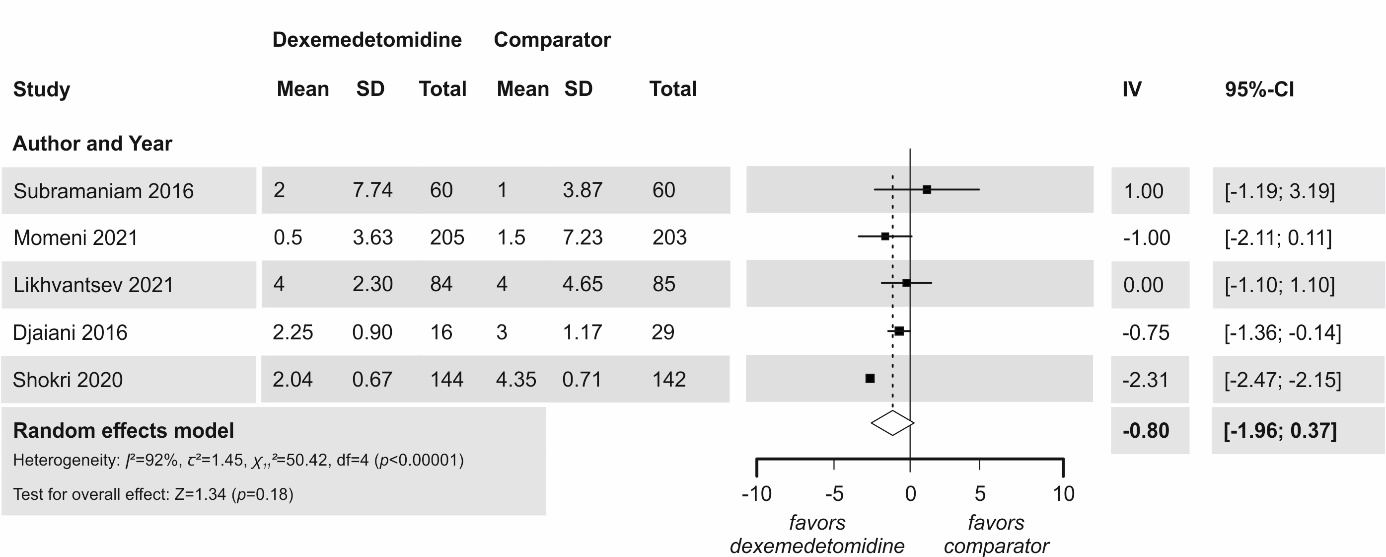
Supplement Figure S17: Forest plot for POD duration after dexmedetomidine administration compared to placebo/other drug

References Supplement:

1. Jones RN, Cizginer S, Pavlech L, et al. Assessment of Instruments for Measurement of Delirium Severity: A Systematic Review. JAMA Intern Med 2019;179(2):231-239. DOI: 10.1001/jamainternmed.2018.6975.

2. Deeken F, Sanchez A, Rapp MA, et al. Outcomes of a Delirium Prevention Program in Older Persons After Elective Surgery: A Stepped-Wedge Cluster Randomized Clinical Trial. JAMA Surg 2022;157(2):e216370. DOI: 10.1001/jamasurg.2021.6370.

3. Wang YY, Yue JR, Xie DM, et al. Effect of the Tailored, Family-Involved Hospital Elder Life Program on Postoperative Delirium and Function in Older Adults: A Randomized Clinical Trial. JAMA Intern Med 2020;180(1):17-25. DOI: 10.1001/jamainternmed.2019.4446.

4. Olotu C, Ascone L, Wiede J, et al. The effect of delirium preventive measures on the occurrence of postoperative cognitive dysfunction in older adults undergoing cardiovascular surgery. The DelPOCD randomised controlled trial. J Clin Anesth 2022;78:110686. DOI: 10.1016/j.jclinane.2022.110686.

5. Marcantonio ER, Flacker JM, Wright RJ, Resnick NM. Reducing delirium after hip fracture: a randomized trial. J Am Geriatr Soc 2001;49(5):516-22. DOI: 10.1046/j.1532-5415.2001.49108.x.

6. Vidan M, Serra JA, Moreno C, Riquelme G, Ortiz J. Efficacy of a comprehensive geriatric intervention in older patients hospitalized for hip fracture: a randomized, controlled trial. J Am Geriatr Soc 2005;53(9):1476-82. DOI: 10.1111/j.1532-5415.2005.53466.x.

7. Guo Y, Fan Y. A Preoperative, Nurse-Led Intervention Program Reduces Acute Postoperative Delirium. J Neurosci Nurs 2016;48(4):229-35. DOI: 10.1097/JNN.0000000000000220.

8. Partridge JS, Harari D, Martin FC, et al. Randomized clinical trial of comprehensive geriatric assessment and optimization in vascular surgery. Br J Surg 2017;104(6):679-687. DOI: 10.1002/bjs.10459.

9. Hempenius L, Slaets JP, van Asselt D, de Bock GH, Wiggers T, van Leeuwen BL. Outcomes of a Geriatric Liaison Intervention to Prevent the Development of Postoperative Delirium in Frail Elderly Cancer Patients: Report on a Multicentre, Randomized, Controlled Trial. PLoS One 2013;8(6):e64834. DOI: 10.1371/journal.pone.0064834.

10. Radtke FM, Franck M, Lendner J, Kruger S, Wernecke KD, Spies CD. Monitoring depth of anaesthesia in a randomized trial decreases the rate of postoperative delirium but not postoperative cognitive dysfunction. Br J Anaesth 2013;110 Suppl 1:i98-105. DOI: 10.1093/bja/aet055.

11. Chan MT, Cheng BC, Lee TM, Gin T, Group CT. BIS-guided anesthesia decreases postoperative delirium and cognitive decline. J Neurosurg Anesthesiol 2013;25(1):33-42. DOI: 10.1097/ANA.0b013e3182712fba.

12. Whitlock EL, Torres BA, Lin N, et al. Postoperative delirium in a substudy of cardiothoracic surgical patients in the BAG-RECALL clinical trial. Anesth Analg 2014;118(4):809-17. DOI: 10.1213/ANE.0000000000000028.

13. Wang E, Wang L, Ye C, et al. Effect of Electroencephalography Spectral Edge Frequency (SEF) and Patient State Index (PSI)-Guided Propofol-Remifentanil Anesthesia on Delirium After Laparoscopic Surgery: The eMODIPOD Randomized Controlled Trial. J Neurosurg Anesthesiol 2022;34(2):183-192. DOI: 10.1097/ANA.0000000000000823.

14. Zhou Y, Li Y, Wang K. Bispectral Index Monitoring During Anesthesia Promotes Early Postoperative Recovery of Cognitive Function and Reduces Acute Delirium in Elderly Patients with Colon Carcinoma: A Prospective Controlled Study using the Attention Network Test. Med Sci Monit 2018;24:7785-7793. DOI: 10.12659/MSM.910124.

15. Wildes TS, Mickle AM, Ben Abdallah A, et al. Effect of Electroencephalography-Guided Anesthetic Administration on Postoperative Delirium Among Older Adults Undergoing Major Surgery: The ENGAGES Randomized Clinical Trial. JAMA 2019;321(5):473-483. DOI: 10.1001/jama.2018.22005.

16. Kunst G, Gauge N, Salaunkey K, et al. Intraoperative Optimization of Both Depth of Anesthesia and Cerebral Oxygenation in Elderly Patients Undergoing Coronary Artery Bypass Graft Surgery-A Randomized Controlled Pilot Trial. J Cardiothorac Vasc Anesth 2020;34(5):1172-1181. DOI: 10.1053/j.jvca.2019.10.054.

17. Sponholz C, Schuwirth C, Koenig L, et al. Intraoperative reduction of vasopressors using processed electroencephalographic monitoring in patients undergoing elective cardiac surgery: a randomized clinical trial. J Clin Monit Comput 2020;34(1):71-80. DOI: 10.1007/s10877-019-00284-1.

18. Tang CJ, Jin Z, Sands LP, et al. ADAPT-2: A Randomized Clinical Trial to Reduce Intraoperative EEG Suppression in Older Surgical Patients Undergoing Major Noncardiac Surgery. Anesth Analg 2020;131(4):1228-1236. DOI: 10.1213/ANE.0000000000004713.

19. Cotae AM, Tiglis M, Cobilinschi C, Baetu AE, Iacob DM, Grintescu IM. The Impact of Monitoring Depth of Anesthesia and Nociception on Postoperative Cognitive Function in Adult Multiple Trauma Patients. Medicina (Kaunas) 2021;57(5). DOI: 10.3390/medicina57050408.

20. Evered LA, Chan MTV, Han R, et al. Anaesthetic depth and delirium after major surgery: a randomised clinical trial. Br J Anaesth 2021;127(5):704-712. DOI: 10.1016/j.bja.2021.07.021.

21. Xu N, Li LX, Wang TL, et al. Processed Multiparameter Electroencephalogram-Guided General Anesthesia Management Can Reduce Postoperative Delirium Following Carotid Endarterectomy: A Randomized Clinical Trial. Front Neurol 2021;12:666814. DOI: 10.3389/fneur.2021.666814.

22. Soehle M, Dittmann A, Ellerkmann RK, Baumgarten G, Putensen C, Guenther U. Intraoperative burst suppression is associated with postoperative delirium following cardiac surgery: a prospective, observational study. BMC Anesthesiol 2015;15:61. DOI: 10.1186/s12871-015-0051-7.

23. Fritz BA, Kalarickal PL, Maybrier HR, et al. Intraoperative Electroencephalogram Suppression Predicts Postoperative Delirium. Anesth Analg 2016;122(1):234-42. DOI: 10.1213/ANE.0000000000000989.

24. Fritz BA, Maybrier HR, Avidan MS. Intraoperative electroencephalogram suppression at lower volatile anaesthetic concentrations predicts postoperative delirium occurring in the intensive care unit. Br J Anaesth 2018;121(1):241-248. DOI: 10.1016/j.bja.2017.10.024.

25. Fritz BA, King CR, Ben Abdallah A, et al. Preoperative Cognitive Abnormality, Intraoperative Electroencephalogram Suppression, and Postoperative Delirium: A Mediation Analysis. Anesthesiology 2020;132(6):1458-1468. DOI: 10.1097/ALN.0000000000003181.

26. Pedemonte JC, Plummer GS, Chamadia S, et al. Electroencephalogram Burst-suppression during Cardiopulmonary Bypass in Elderly Patients Mediates Postoperative Delirium. Anesthesiology 2020;133(2):280-292. DOI: 10.1097/ALN.0000000000003328.

27. Gutierrez R, Egana JI, Saez I, et al. Intraoperative Low Alpha Power in the Electroencephalogram Is Associated With Postoperative Subsyndromal Delirium. Front Syst Neurosci 2019;13:56. DOI: 10.3389/fnsys.2019.00056.

28. Tanabe S, Mohanty R, Lindroth H, et al. Cohort study into the neural correlates of postoperative delirium: the role of connectivity and slow-wave activity. Br J Anaesth 2020;125(1):55-66. DOI: 10.1016/j.bja.2020.02.027.

29. Acker L, Ha C, Zhou J, et al. Electroencephalogram-Based Complexity Measures as Predictors of Post-operative Neurocognitive Dysfunction. Front Syst Neurosci 2021;15:718769. DOI: 10.3389/fnsys.2021.718769.

30. Koch S, Windmann V, Chakravarty S, et al. Perioperative Electroencephalogram Spectral Dynamics Related to Postoperative Delirium in Older Patients. Anesth Analg 2021;133(6):1598-1607. DOI: 10.1213/ANE.0000000000005668.

31. Cooter Wright M, Bunning T, Eleswarpu SS, et al. A Processed Electroencephalogram-Based Brain Anesthetic Resistance Index Is Associated With Postoperative Delirium in Older Adults: A Dual Center Study. Anesth Analg 2022;134(1):149-158. DOI: 10.1213/ANE.0000000000005660.

32. Mackenzie K, Williams C. Universal, school-based interventions to promote mental and emotional well-being: what is being done in the UK and does it work? A systematic review. BMJ Open 2018;8(9):e022560. DOI: 10.1136/bmjopen-2018-022560.

33. Punjasawadwong Y, Chau-In W, Laopaiboon M, Punjasawadwong S, Pin-On P. Processed electroencephalogram and evoked potential techniques for amelioration of postoperative delirium and cognitive dysfunction following non-cardiac and non-neurosurgical procedures in adults. Cochrane Database Syst Rev 2018;5:CD011283. DOI: 10.1002/14651858.CD011283.pub2.

34. Bocskai T, Kovacs M, Szakacs Z, et al. Is the bispectral index monitoring protective against postoperative cognitive decline? A systematic review with meta-analysis. PLoS One 2020;15(2):e0229018. DOI: 10.1371/journal.pone.0229018.

35. Chan MTV, Hedrick TL, Egan TD, et al. American Society for Enhanced Recovery and Perioperative Quality Initiative Joint Consensus Statement on the Role of Neuromonitoring in Perioperative Outcomes: Electroencephalography. Anesth Analg 2020;130(5):1278-1291. DOI: 10.1213/ANE.0000000000004502.

36. Miao M, Xu Y, Sun M, Chang E, Cong X, Zhang J. BIS index monitoring and perioperative neurocognitive disorders in older adults: a systematic review and meta-analysis. Aging Clin Exp Res 2020;32(12):2449-2458. DOI: 10.1007/s40520-019-01433-x.

37. Sun Y, Ye F, Wang J, et al. Electroencephalography-Guided Anesthetic Delivery for Preventing Postoperative Delirium in Adults: An Updated Meta-analysis. Anesth Analg 2020;131(3):712-719. DOI: 10.1213/ANE.0000000000004746.

38. Shan W, Chen B, Huang L, Zhou Y. The Effects of Bispectral Index-Guided Anesthesia on Postoperative Delirium in Elderly Patients: A Systematic Review and Meta-Analysis. World Neurosurg 2021;147:e57-e62. DOI: 10.1016/j.wneu.2020.11.110.

39. Sumner M, Deng C, Evered L, et al. Processed electroencephalography-guided general anaesthesia to reduce postoperative delirium: a systematic review and meta-analysis. Br J Anaesth 2022. DOI: 10.1016/j.bja.2022.01.006.
